# Systematic review and meta-analysis of root morphology and canal configuration of permanent premolars using cone-beam computed tomography

**DOI:** 10.1186/s12903-024-04419-y

**Published:** 2024-06-04

**Authors:** Mengchen Xu, Huiying Ren, Congrui Liu, Xinyu Zhao, Xiaoyan Li

**Affiliations:** 1 Department of Endodontics, School and Hospital of Stomatology, Cheeloo College of Medicine, Shandong University & Shandong Key Laboratory of Oral Tissue Regeneration & Shandong Engineering Research Center of Dental Materials and Oral Tissue Regeneration & Shandong Provincial Clinical Research Center for Oral Diseases, Shandong University, Jinan, Shandong 250012 China; 2 Science and Technology Innovation Committee of Shenzhen Municipality, Shenzhen Research Institute of Shandong University, A301 Virtual University Park in South District of Shenzhen, Shenzhen, Guangdong 518000 China; 3 Department of stomatology, Jinan Hospital, Jinan, Shandong 250013 China

**Keywords:** Root morphology, Canal configuration, Premolar, Systematic review, Meta-analysis

## Abstract

**Introduction:**

The efficacy of root canal treatment is greatly impacted by a thorough understanding of root canal anatomy. This systematic review and meta-analysis aim to thoroughly investigate the root morphology and canal configuration (RMCC) of permanent premolars (PMs).

**Methodology:**

A comprehensive analysis was conducted following the PRISMA guidelines. Literature exploration was carried out across four electronic databases (PubMed, Embase, Cochrane, and Web of Science). The risk of bias assessment was conducted for the included studies utilizing the Anatomical Quality Assessment (AQUA) tool. Data analysis was performed utilizing SPSS and RevMAN5.3.3. The meta-analysis was applied with a 95% confidence interval to calculate odds ratios (OR).

**Results:**

Among the 82 selected studies, 59 studies exhibited potential bias in domain one (objective(s) and subject characteristics), followed by domain three (methodology characterization). The majority of maxillary PM1s had either single root (46.7%) or double roots (51.9%), while three-rooted variants were uncommon (1.4%). Conversely, most other PMs exhibited a single root. In terms of canal configuration, maxillary PM1s predominantly featured double distinct canals (87.2%), with the majority of maxillary PM2s displaying either a single canal (51.4%) or double canals (48.3%). Mandibular PMs were primarily characterized by single canals, accounting for 78.3% of mandibular PM1s and 90.3% of mandibular PM2s. Subgroup analyses revealed higher incidences of single-rooted and single-canalled PMs among Asians compared to Caucasians. Additionally, women exhibited a higher incidence of single-rooted PMs, while men showed a greater frequency of double-rooted PMs.

**Conclusions:**

The comprehensive analysis indicated that maxillary PM1s predominantly possess double roots and double canals, whereas maxillary PM2s and mandibular PMs were primarily characterized by single-rooted with a single canal. Notably, single root and single canal were more prevalent among women and Asian samples.

**Supplementary Information:**

The online version contains supplementary material available at 10.1186/s12903-024-04419-y.

## Introduction

The effectiveness of endodontic treatment hinges significantly on a precise understanding of tooth morphology. Even subtle anatomical variations in the root canal system present challenges for dentists and endodontists, elevating the risk of treatment failure. Studies have shown a varied success rate of root canal treatment (RCT, refer to supplementary Table 1 for abbreviations), ranging from 75.3–96% [1, 2]. A retrospective cohort study enrolled 1262 patients revealed that untreated additional canals contribute the most to endodontic failure within five years post-initial treatment [3]. Natural anatomical complexities in the middle and apical third of the canal often necessitate surgical retreatment in certain cases [4]. The root morphology and canal configuration (RMCC) system, considering factors like the number, curvature level, and direction of the root canal, substantially influences the difficulty of treatment [5]. Consequently, a thorough understanding of RMCC is essential for both nonsurgical and surgical endodontic procedures.

Root canal morphology research has evolved significantly, shifting from two-dimensional analyses to more comprehensive three-dimensional techniques. This evolution involves a transition from localized investigations to a holistic approach and from destructive specimen manipulation to non-destructive methodologies. While traditional techniques like slicing, grinding, and the transparent tooth method remain relevant in general scientific inquiry and pedagogical contexts [6, 7], non-destructive digital imaging systems, such as cone-beam computed tomography (CBCT) and micro-computed tomography, have deepened our understanding of complex RMCCs and led to increased reports on intricate root canals [8, 9]. Although previous studies have reviewed the literature on premolar RMCCs and confirmed their extreme complexity [10, 11], data need updating based on new studies with precise three-dimensional images. This update is crucial for aligning anatomical data with contemporary diagnostic methods effectively.

In recent years, an increasing number of studies have revealed the intricate nature of PM root canal systems, showcasing a broad spectrum of anatomical variations like intricate canal shapes, multiple roots, and root surface sulci [12, 13]. Such intricate variations pose considerable challenges in attaining optimal cleaning and shaping, contributing to the substantial failure rate in premolar RCTs. The condition of RMCC may be influenced by various factors, including age, race, and gender [14, 15]. Martins et al. [16] noted alterations in root canal morphologies over an individual’s lifespan, with distinct canal types being impacted by the gradual accumulation of secondary dentin. Torres et al. [17] documented discrepancies in the RMCC of mandibular molars between Belgium and Chile, with variations also noted between Asians and Caucasians. Additionally, root canal characteristics vary based on tooth position, as evidenced by a relatively higher occurrence of C-shaped canals in mandibular second molars compared to mandibular first molars. Although certain scholars have explored gender-related variables, their viewpoints diverge, highlighting the need for additional investigations. Therefore, the paper aims to conduct a comprehensive evaluation and systematic review of the existing literature on root anatomy and canal architecture of permanent PMs, elucidating the influencing factors and providing further support for clinical treatment.

## Methodology

### Research design

The systematic review, accompanied by a meta-analysis, was duly registered in the International Prospective Register of Ongoing Systematic Reviews (PROSPERO) under the registration number CRD42024500006, ensuring adherence to established research protocols and transparency in reporting. The review process was meticulously conducted following the guidelines laid out in the Preferred Reporting Items for Systematic Review and Meta-analysis (PRISMA).

### Literature search strategy

A thorough investigation was conducted by systematically searching across four electronic databases (PubMed, Embase, Cochrane, and Web of Science). A standardized and thorough search strategy was implemented, utilizing the Medical Subject Heading (MeSH) terms provided in Table 1 and supplementary Table 2. Moreover, supplementary studies were incorporated through cross-referencing and manual search of full-text article bibliographies. Randomized controlled trials, cross-sectional, comparative, validation and evaluation studies focusing on RMCC of human premolars across various populations were included. The timeframe for publications encompassed the entire duration since the establishment of these databases until the present, ensuring a comprehensive coverage of the available literature. Two investigators (Xu. and Ren.) independently conducted a review of the extracted research based on the following inclusion criteria: original full-length articles published in English, which reported on one or more of the study variables pertaining to permanent premolars, including the number of roots, number of canals, Vertucci’s classification system and C-shaped canals. Studies, such as case reports, reviews, and editorials, were all excluded from the analysis. Studies that did not evaluate RMCC by Vertucci classification or did not take intercanal communications into account were excluded from this meta-analysis. Two investigators (Xu. and Ren.) independently evaluated the titles and abstracts, subsequently conducting a meticulous examination of the full-text articles. Any discrepancies were resolved through deliberation to achieve a consensus, or by consulting a third investigator for input.

**Table 1:** Search strategy for PubMed

Search	Query
#1	“bicuspid” [Mesh]
#2	“bicuspid* or premolar*” [All fields]
#3	#1 OR #2
#4	“dental pulp cavity” [Mesh]
#5	“cavit* or chamber* or canal*” [All fields]
#6	#4 OR #5
#7	“cone-beam computed tomography” [Mesh]
#8	“CT or cone beam computer assisted” [All fields]
#9	#7 OR #8
#10	#3 AND #6 AND #9

### Quality assessment

Two independent reviewers (Xu. and Ren.) employed the AQUA tool [18], tailored for anatomical studies, to assess the quality of the included research. The AQUA tool comprises five domains: objective(s) and subject characteristics, study design, methodology characterization, descriptive anatomy, and reporting of results. Each domain features signaling questions to assist in bias risk assessment, with responses categorized as “Yes,” “No,” or “Unclear.” A “Yes” response across all signaling questions within a domain indicates a “Low” bias risk. Conversely, any “No” or “Unclear” responses imply potential bias risk.

### Data extraction

The following parameters were gathered from the covered studies: “first author, year, region, research tool, investigated variables, number, gender and age of subjects, as well as the type and number of teeth”. The primary outcomes encompassed the number of roots, the number of canals, and root canal morphology. To facilitate data organization and analysis, the gathered information was tabulated in a spreadsheet file, with categorization based on the type of teeth being investigated. The occurrence and percentage of each variable, along with the total count for each classification, were methodically investigated and documented.

### Recording and root canal classification

**Fig. 1:**
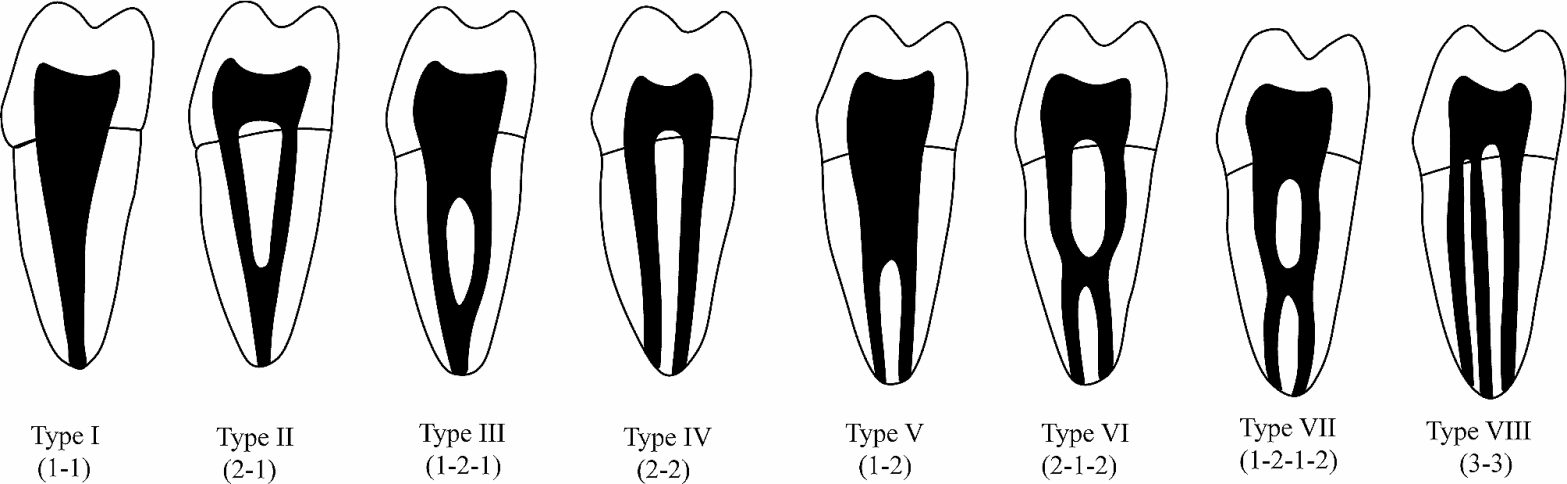
The illustration of root canal configurations based on the Vertucci method

The data to be documented includes (1) the number of roots, (2) the number of canals, and (3) the classification of root canal configurations based on the Vertucci method (Fig. 1) [19].

### Statistical analysis

The frequencies of varying root numbers, canal numbers, and root canal configuration were regarded as the main outcome variables. Statistical comparisons were conducted utilizing SPSS and RevMAN5.3.3 software to assess differences across different races and genders. Data will be grouped by participant ethnicity, with over 50% Caucasian participants categorized as Caucasian group and over 50% Asian participants categorized as Asian group [20, 21]. The meta-analysis outcomes were visually presented in forest plots, presenting the odds ratio (OR) with a 95% confidence interval (CI). *I*^*2*^ was used to measure the heterogeneity and the random or fixed effects model of meta-analysis was performed based on the magnitude of heterogeneity (*I*^*2*^ < 35%: fixed-effect, *I*^*2*^ > 35%: random-effect) [22]. *P* ≤ .05 was considered statistically significant.

## Results

### Literature selection

**Fig. 2:**
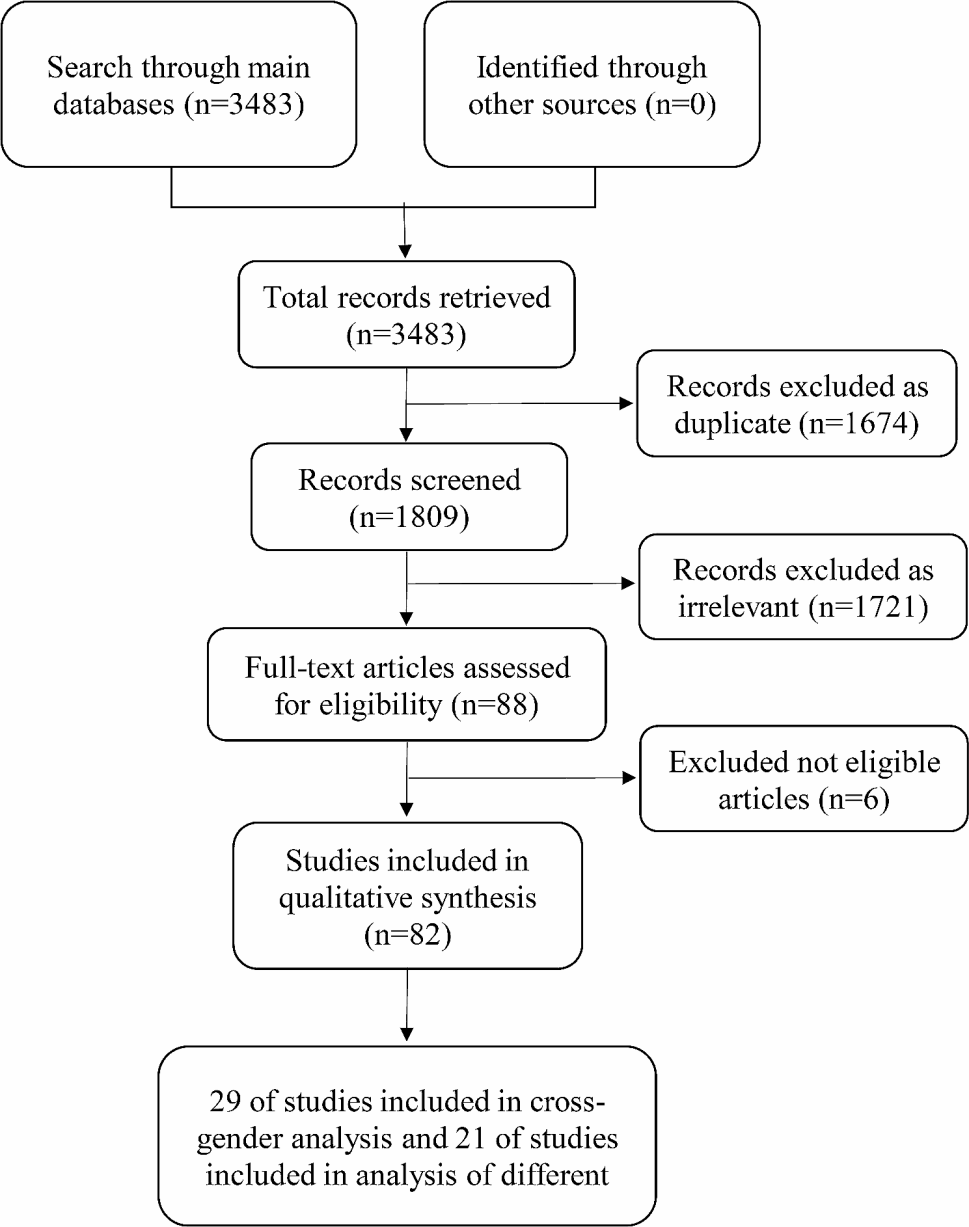
Flowchart of the literature screening process

A comprehensive search across four electronic databases (PubMed, Embase, Cochrane, and Web of Science) yielded a total of 3,483 pieces of literature. During the initial screening stage, 1,674 studies were identified as duplicates and subsequently eliminated. Additionally, 1,721 studies were excluded based on their titles and abstracts (e.g., abstracts, case reports, editorials). Six studies were excluded due to insufficient information pertaining to the principal variables of the study. Ultimately, a total of 82 studies ( [23–104]) were included in the qualitative analysis. The comprehensive procedure of literature screening was depicted in Fig. 2.

### Characteristics of the included studies

The present analysis incorporates a sample size of 82 studies, among which, 29 specifically focused on gender categorization and 21 studies examined dental position classification. The final compilation of literature encompasses publications from 2012 to 2023 and incorporates data from diverse countries, including but not limited to China, Germany, Israel, South Africa, Portugal, Egypt, India, Saudi Arabia, Turkey, Spain, and Poland.

### Risk of bias assessment

**Fig. 3:**
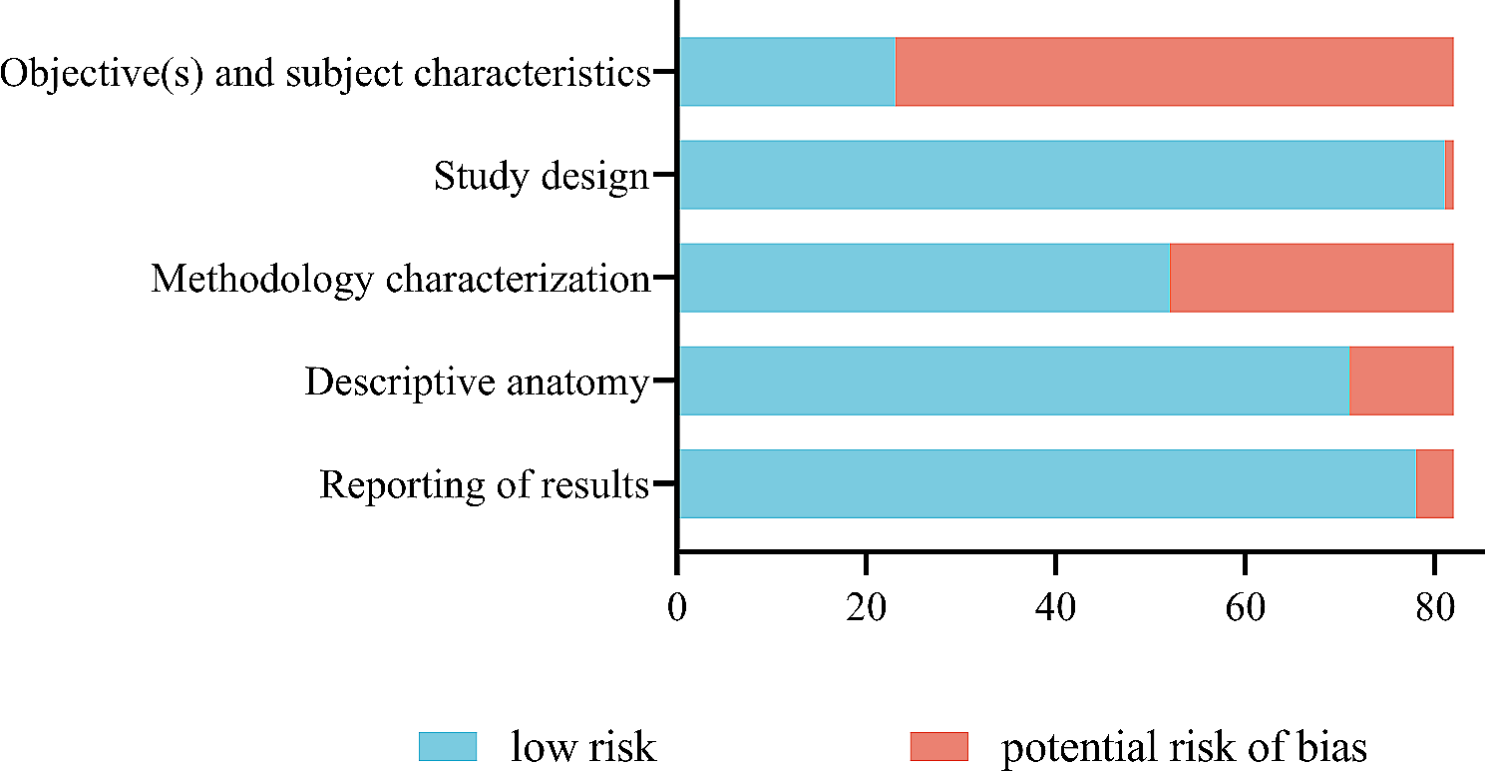
Bias risk chart: evaluators’ assessments of each domain of bias (AQUA tool)

The risk of bias assessment outcomes for the included studies are displayed in Fig. 3. Among the selected studies, a majority (59 out of 82) exhibited potential bias in domain one (objective(s) and subject characteristics), primarily due to insufficient elaboration or clarification regarding sample size calculation methods. Additionally, some studies indicated potential bias in domain three (methodology characterization), specifically concerning the absence of details regarding the medical specialty and experience of the researchers, as well as measures to mitigate inter and intra-observer variability. Domains two (study design), four (descriptive anatomy), and five (reporting of results) were generally found to be unbiased. However, some studies within these domains were deemed biased due to unclear or incomprehensible study methods, figures, or reported outcomes.

### Number of roots and canals

The distribution of maxillary PM1s was nearly evenly divided between single-rooted and double-rooted teeth, with each category comprising approximately 50% of the sample. Notably, 87.2% of maxillary PM1s exhibited double canals. In contrast, the dominant characteristic for other PMs was single root and single canal. Single-rooted teeth were found to be more prevalent in mandibular PMs than in maxillary PMs. Furthermore, PM2s demonstrated a higher occurrence of single roots in comparison to PM1s. Further information can be found in supplementary Table 3.

#### Maxillary PM1s

Among the 18,536 maxillary PM1s examined in terms of the number of roots, 46.7% (*N* = 8,660) were classified as single root, 51.9% (*N* = 9,621) as double roots and 1.4% (*N* = 254) as three roots. Regarding canal count, 87.2% (*N* = 7,799) of 8,945 maxillary PM1s exhibited double canals, 10.7% (*N* = 955) displayed single canal and only 2.1% (*N* = 189) demonstrated the presence of three canals.

#### Maxillary PM2s

The study analyzed 16,371 maxillary PM2s, revealing that 84.3% (*N* = 13,803) had a single root, 15.4% (*N* = 2,525) possessed double roots and a mere 0.3% (*N* = 43) had three roots. Regarding canal count, 51.4% (*N* = 5,399) exhibited single canal, 48.3% (*N* = 5,073) displayed double canals and only 0.2% (*N* = 26) had three canals.

#### Mandibular PM1s

This research scrutinized 18,655 mandibular PM1s in total. Most of these teeth exhibited a solitary root (94.4%, *N* = 17,604) and a lone canal (78.3%, *N* = 8,899). The statistical evaluation showed the presence of 1,024 teeth with dual roots, accounting for 5.5% of the total sample. Merely a handful of research works documented 24 instances of tri-rooted teeth, representing just 0.1% of the total. It was rare to find several canals, evidenced by 2,401 teeth possessing two (21.1%) and merely 61 teeth having three (0.5%).

#### Mandibular PM2s

The study analyzed a total of 17,939 mandibular PM2s, of which the majority exhibited a single root (97.7%, *N* = 17,519) and a single canal (90.3%, *N* = 8,392). A minor segment of the sample was made up of 401 double-rooted teeth, accounting for 2.2% of the overall count. Merely five research works, namely Gündüz, H. et al. [23], Buchanan, G.D., et al. [24], Hasheminia, S.M., et al. [25], Alfawaz, H., et al. [26] and Burklein, S., et al. [27], reported a total of 19 cases of three roots, accounting for 0.1%. Similarly, the occurrence of multiple canals was infrequent, with 872 teeth exhibiting double canals, representing 9.4% of the total sample and 19 teeth displaying three canals, accounting for 0.2% of the sample.

### Root canal configurations

In the analysis of maxillary PM1s, Vertucci type IV showed the most significant occurrence rate at 54.8%, followed by Vertucci type II and Vertucci type I. Within maxillary PM2s, the proportion of Vertucci type I increased to 49.0%, establishing itself as the most prevalent configuration. Similarly, in mandibular PMs, Vertucci type I continued to be the most prevalent configuration. In-depth details were available in supplementary Table 4.

#### Maxillary PM1s

Upon analyzing the root canal morphology of 18,627 teeth, the prevalence of Vertucci types was observed as follows: Type I constituted 16.2% (*N* = 3,023), Type II constituted 15.9% (*N* = 2.957), Type III constituted 3.8% (*N* = 706), Type IV had the highest proportion at 554.8% (*N* = 10,200), Type V constituted 4.7% (*N* = 883), Type VI constituted 2.0% (*N* = 373), Type VII constituted 0.6% (*N* = 106), Type VIII constituted 1.2% (*N* = 218) and 160 teeth with various other canal shapes.

#### Maxillary PM2s

Research on 15,806 maxillary PM2s’ root canals revealed that Vertucci type I accounted for 49.0% (*N* = 7,738), type II occupied 16.2% (*N* = 2,564), type IV occupied 16.9% (*N* = 2,676), type III occupied 7.2% (*N* = 1,144), type V occupied 6.6% (*N* = 1,045) and type VI accounted for 2.2% (*N* = 340). The occurrences of the other categories, specifically type VII (1.0%, *N* = 155), type VIII (0.4%, *N* = 65) and others (0.5%, *N* = 79), were minimal.

#### Mandibular PM1s

The root canal morphology of 23,198 mandibular PM1s was analyzed, among which Vertucci type I accounted for 76.3% (*N* = 17,694), followed by type V at 13.3% (*N* = 3,091), which together accounted for almost 90% of the root canal morphology of mandibular PM1s. The remaining types, in descending order of prevalence, were type III (3.9%, *N* = 900), type IV (2.3%, *N* = 537), type II (1.8%, *N* = 410), type VI (0.5%, *N* = 105), type VIII (0.2%, *N* = 49) and type VII (0.2%, *N* = 36). Furthermore, the study uncovered 152 instances of C-shaped canals and an extra 218 canals with a range of other morphological features.

#### Mandibular PM2s

Analyses of the root canal morphology in a set of 21,317 teeth unveiled the commonness of Vertucci types I (92.5%, *N* = 19,711), V (3.9%, *N* = 824), II (1.3%, *N* = 268), III (0.8%, *N* = 178), IV (0.5%, *N* = 101) and VI (0.3%, *N* = 54). It is worth noting that a mere 28 teeth exhibited Vertucci type VIII, 10 teeth displayed Vertucci type VII and 32 teeth exhibited a C-shaped canal, collectively making up no more than 1% of the entire sample.

### Interracial analysis

Examining racial disparities shows a higher occurrence rate of single root and canal events in Asians than in Caucasians. Correspondingly, Asians exhibit a lower frequency of double roots and double root canals. Within the group of maxillary PM1s, Vertucci IV emerged as the predominant type, demonstrating a greater prevalence in Caucasians (57.5%) as opposed to Asians (46.8%). Regarding maxillary PM2s and mandibular PMs, Vertucci I was the most prevalent, particularly among Asians. The root and canal analysis of Caucasians and Asians can be found in supplementary Tables 5 and supplementary Table 6, respectively, while the analysis of interracial canal morphology is presented in supplementary Tables 7 and supplementary Table 8.

#### Maxillary PM1s

Within the Caucasian cohort, 38.4% (*N* = 4,725) were attributed to single root, 60.0% (*N* = 7,390) to double roots, 1.6% (*N* = 201) to three roots, whereas among Asians, 68.2% (*N* = 3,462) to single roots, 31.2% (*N* = 1,584) to double roots and a mere 0.6% (*N* = 30) to three roots. Remarkably, most people in Caucasian and Asian groups showed the presence of double canals, with Caucasians at 87.3% and Asians at 86.4% prevalence. Regarding the structure of canals, the Caucasian cohort exhibited a greater occurrence of type IV (57.5% in Caucasians and 46.8% in Asians), type I (16.5% in Caucasians and 15.7% in Asians), and type VIII (1.3% in Caucasians and 0.6% in Asians) in contrast to the Asian group. Correspondingly, the prevalences of type II (14.2% in Caucasians and 20.7% in Asians), type III (2.9% in Caucasians and 5.8% in Asians), type V (4.1% in Caucasians and 6.7% in Asians) and type VI (1.9% in Caucasians and 2.3% in Asians) were reduced in the Caucasian cohort.

#### Maxillary PM2s

In the Caucasian population, the prevalence of single-rooted teeth was found to be 80.9% (*N* = 9,246), while double-rooted teeth accounted for 18.7% (*N* = 2,137) and three-rooted teeth accounted for 0.3% (*N* = 39). In the Asian population, single-rooted teeth constituted 93.2% (*N* = 3,780), double-rooted teeth accounted for 6.8% (*N* = 274). Fascinatingly, the distribution of single and double canals was almost identical in both groups, where the Caucasian group had 50.5% with a single canal and 49.2% with double canals, while the Asian group had 54.4% with a single canal and 47.5% with double canals. Regarding canal morphology, the Asian group exhibited a higher prevalence of type I (48.8% in Caucasians and 50.3% in Asians), type II (14.4% in Caucasians and 21.6% in Asians) and type III (6.1% in Caucasians and 10.6% in Asians) compared to the Caucasian group. Conversely, the Caucasian group demonstrated higher prevalences of type IV, V, VI, VII, and VIII.

#### Mandibular PM1s

Relative to Caucasians, individuals of Asian descent exhibit higher frequencies of single root and single canal (92.7% single root and 78.7% single canal in Caucasians, 96.9% single root and 75.7% single canal in Asians). Specifically, type I (78,6% in Asians and 75.9% in Caucasians) and type V (13.8% in Asians and 12.8% in Caucasians) root canal morphologies were more prevalent among Asians, while the remaining root canal morphologies were more commonly observed in Caucasians.

#### Mandibular PM2s

In comparison to the Caucasian cohort, it has been observed that Asian individuals were more prone to have both a single root and a single canal in mandibular PM2s (97.1% single root and 89.2% single canal in Caucasians, 100.0% single root and 98.8% single canal in Asians). Among the various root canal morphologies, Vertucci I was found to be the most prevalent, with a notably higher occurrence in the Asian population (98.6%) as opposed to Caucasians (90.7%). Other varieties of root canal structures were seldom found, with the majority of these types appearing in under 1% of cases.

### Cross-gender analysis

**Fig. 4:**
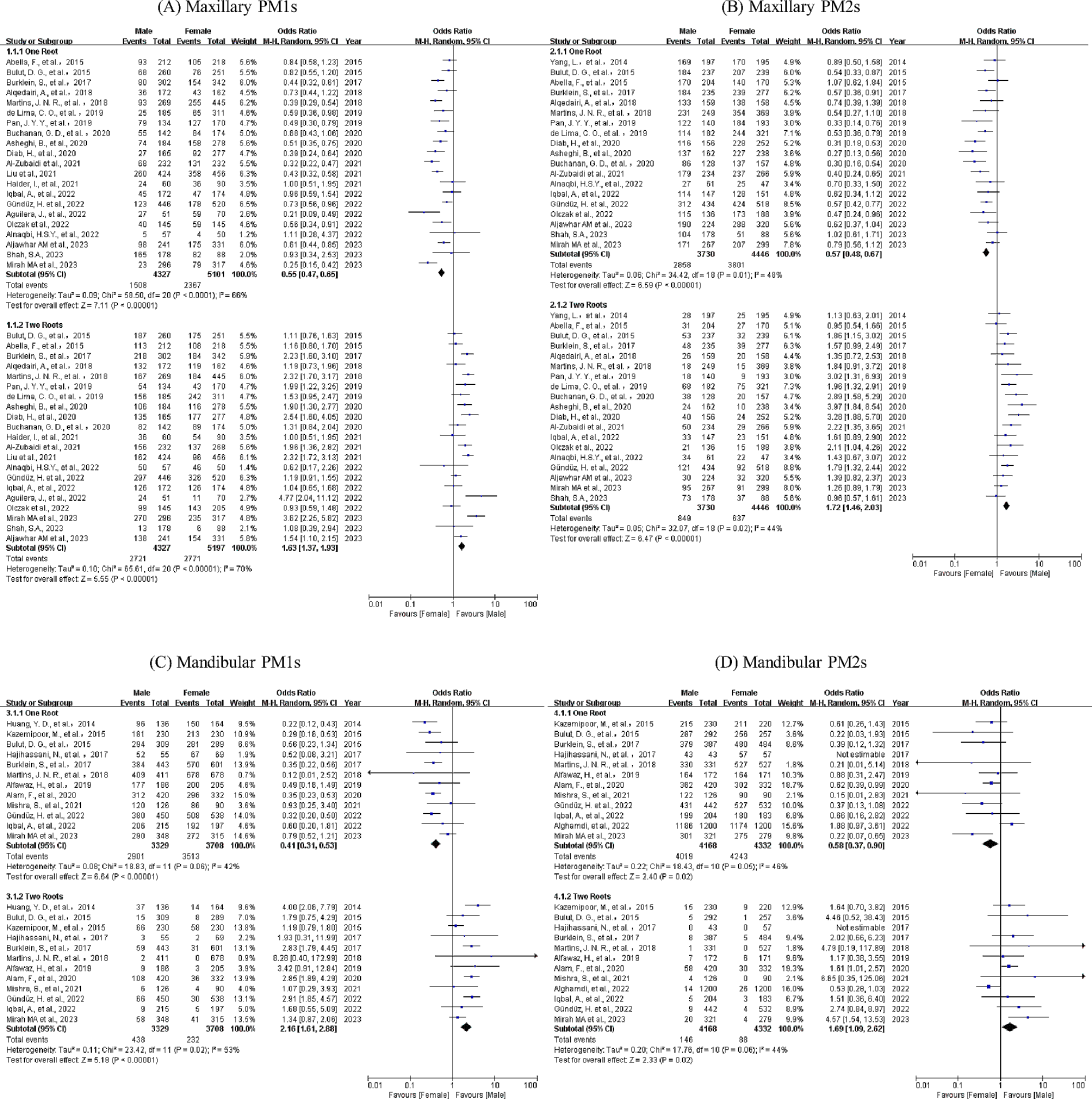
The forest plots comparing root numbers between genders

A total of 29 research projects were carried out to explore the link between gender and the number of roots, as depicted in Fig. 4. Owing to the rare presence of three roots, our study concentrated exclusively on a single root and double roots. Results were displayed using forest plots, showcasing odds ratios and a 95% CI. Statistical importance was ascertained through the *P* value, whereas diversity was evaluated via the *I*^*2*^ statistic.

Females showed a notable preference for the peto odds ratio of a single root (maxillary PM1s 95% CI: [0.47, 0.65], maxillary PM2s 95% CI: [0.48, 0.67], mandibular PM1s 95% CI: [0.31, 0.53] and mandibular PM2s 95% CI: [0.37, 0.90]), while for double roots, the preference was for males (maxillary PM1s 95% CI: [1.37, 1.93], maxillary PM2s 95% CI: [1.46, 2.03], mandibular PM1s 95% CI: [1.61, 2.88] and mandibular PM2s 95% CI: [1.09, 2.62]). Notable disparities were detected in the relationship between gender and root number (*P < .05*).

### Bilateral symmetry

**Fig. 5:**
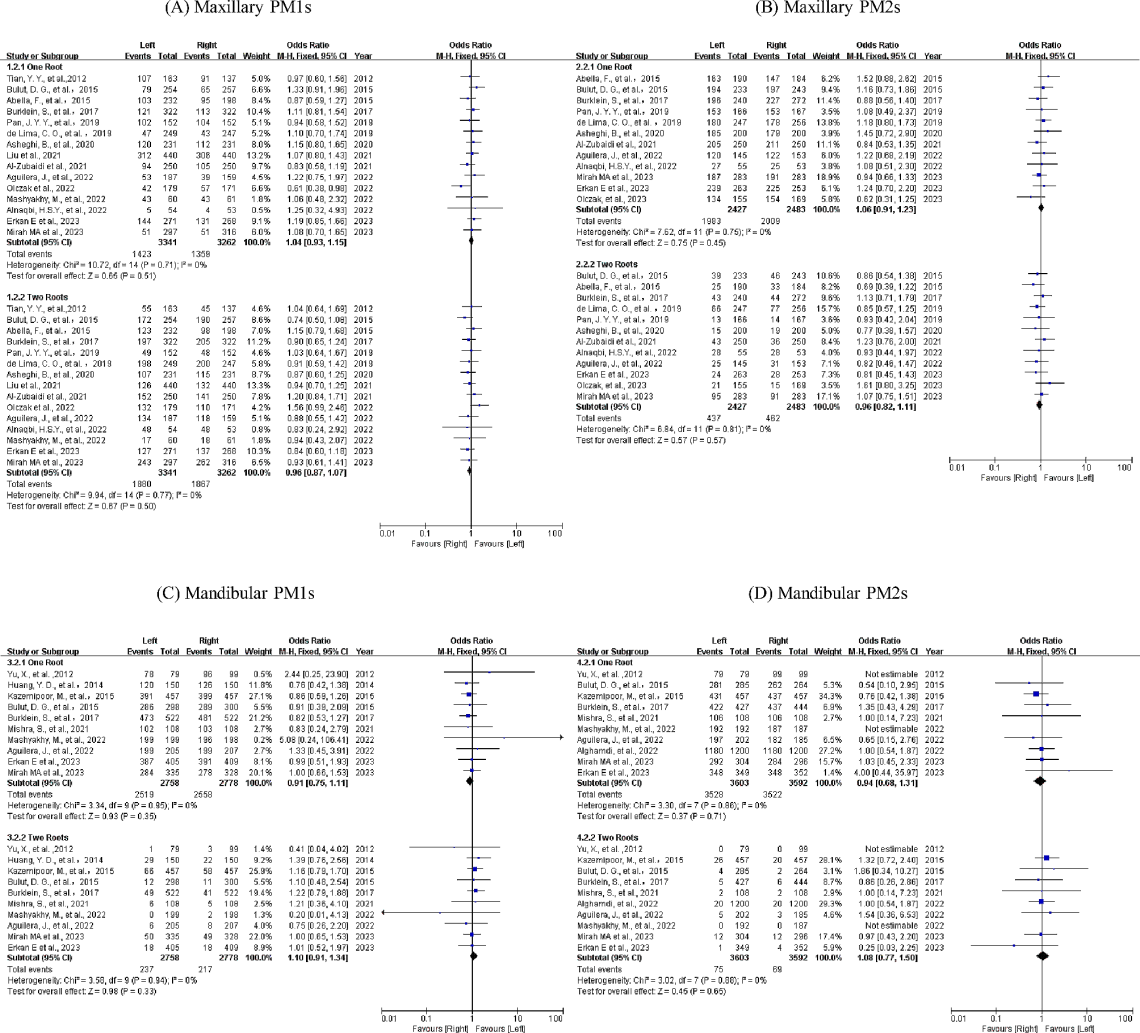
The forest plots comparing root numbers between tooth positions

The meta-analysis incorporated 21 studies to investigate the relationship between the location of teeth and the number of roots (Fig. 5). For every case, the 95% CI of the peto odds ratio encompassed the value of 1, indicating the absence of a statistically significant distinction between tooth position and the number of roots *(P > .05)*.

## Discussion

Since 1990, dental research has increasingly utilized CBCT, offering numerous benefits such as high resolution, minimal radiation dose, rapid image capture, three-dimensional reconstruction capabilities, minimal distortion, and superior visualization of dense tissues [105]. While CBCT is regardedas reliable and repeatable, its lower image resolution compared to micro-CT may impede its capacity to detect more intricate anatomical structures [106]. Pires et al. [107] simultaneously scanned mandibular premolars using CBCT (200 μm voxel size) and micro-CT (19.61 μm) and performed Vertucci’s classification. The results showed a high percentage of consistency between the two methods with 85.2%. Neelakantan et al. [108] also demonstrated that CBCT achieves an equivalent level of precision in identifying root canal anatomy as the clear tooth method, which is considered the gold standard. Therefore, we conducted a multicenter CBCT cross-sectional research with meta-analysis.

This study conducted a comprehensive analysis of 82 relevant literature sources spanning from 2012 to 2023. Among the total of 18,536 maxillary PM1s examined, single root accounted for 46.7%, double roots for 51.9%, and three-root cases were infrequent at 1.4%. Similarly, for maxillary PM2s (16,371 instances), mandibular PM1s (18,655 instances), and mandibular PM2s (17,939 instances), single roots were predominant, accounting for over 80% of the cases. Concerning the number of root canals, double canals were more prevalent in maxillary PM1s (87.2%), while most maxillary PM2s displayed either a single canal (51.4%) or double canals (48.3%). Single canal dominated in mandibular PM1s and PM2s (78.3% and 90.3%, respectively).

The formation of C-shaped canals is mainly attributed to the partial merging of the Hertwig epithelial sheath [109]. The presence of irregular areas within the C-shaped canal presents challenges to the effective clearance of infections. Therefore, it is advisable to utilize small instruments for the preparation of the isthmus region of the root canal and to perform thorough irrigation with sodium hypochlorite during RCT [110, 111]. The incidence of C-shaped root canals in the mandibular PM1 is approximately 0.7%, whereas it is 0.2% in mandibular PM2s. Caution should be exercised during RCT to prevent iatrogenic accidents and ensure proper management.

Major racial differences may significantly affect the RMCC system. Numerous investigations have examined racial disparities in root canal morphology, such as those conducted on the German [27], Indian [28], Chinese [29], and South African populations [30]. This study unveiled a higher prevalence of single root and canal among Asians compared to Caucasians across the four types of PMs. Regarding root canal morphology, Vertucci II, III, V, and VI demonstrate higher prevalence among individuals of Asian descent in maxillary PM1s. In maxillary PM2s, Vertucci I, II, and III exhibit higher frequencies among Asians. In mandibular PM1s, Vertucci I and V were more commonly observed in Asians and Vertucci I predominates in mandibular PM2s among the Asian population.

Genes linked to canal structure are situated on the X chromosome [112], prompting research efforts to explore how gender disparities affect the RMCC system. Al-Zubaidi et al. [31] reported a higher incidence of single-root maxillary PM1s in women (56.5% vs. 29.3%), while men exhibited a higher prevalence of maxillary PM1s with double roots (67.2% vs. 51.1%). Differently, Alghamdi et al. [32] observed a greater prevalence of single-root mandibular PM2s in men (98.8% vs. 97.8%). This study comprehensively analyzed 29 research studies and found that females exhibited a greater prevalence of single-root PMs, whereas males demonstrated a higher prevalence of double-root PMs.

Symmetry is a universal phenomenon and investigations into the symmetry of root canals can provide insights into the potential anatomical characteristics of corresponding homonymous teeth. Li, Y.-h., et al. conducted a study involving 1387 maxillary PM1s and 1403 PM2s, revealing that 80.2% of the maxillary PM1s and 81.8% of the PM2s displayed bilateral symmetry in the number of root canals [33]. Furthermore, 72.3% of the maxillary PM1s and 73.2% of the maxillary PM2s showed bilateral symmetry in the number and morphology of root canals [33]. This research demonstrated the universal symmetry of root and canal morphology in PMs.

The results of our systematic review with meta-analysis are consistent with those of several previous studies [10, 11], indicating the conclusions have a certain degree of generalizability. However, the study also has the following limitations. Vertucci’s canal classification primarily emphasizes the main root canal configuration [113], overlooking details in secondary canals, accessory structures, and intricate anatomical variations such as isthmuses and apical deltas [114], which are vital for precise diagnosis and treatment planning. Ethnic groups were classified based on patients’ profiles, potentially introducing bias, although conducting genetic tests on a large number of individuals would have been impractical. Additionally, a majority of the studies (59 out of 82) analyzed in this research exhibited bias in the objective(s) and subject characteristics, with 30 out of 82 studies demonstrating bias in methodology characterization, which diminished the reliability and evidential strength of the meta-analysis. While the study sample is representative and includes diverse regions and populations, it primarily examines nationality, gender, and dental positions, overlooking the impact of age and other potential factors.

In conclusion, clinicians must possess a thorough comprehension of the intricate nature, varied composition, and symmetry of the RMCC system when undertaking RCTs on PMs. Employing imaging techniques like CBCT can provide unique, precise, and reliable visual depictions, aiding in a comprehensive grasp of the anatomical traits of the RMCC system. These visual aids are invaluable resources guiding medical professionals toward successful conclusions of RCTs. Future studies could benefit from conducting multi-center meta-analyses focusing on more specific root canal questions, such as isthmuses and apical deltas, to provide more accurate guidance.

## Conclusions

1. The majority of maxillary PM1s displayed either a single or double roots, with 3-rooted variant being rare. Single root predominated in the remaining premolars.
2. Maxillary PM1s were predominantly associated with double canals, while single and double canals were approximately equal in maxillary PM2s. In contrast, mandibular PMs were most commonly associated with a single canal.
3. Vertucci IV predominantly characterized maxillary PM1s, whereas Vertucci I was more common in various other PM types.
4. Within the quartet of PM categories, Asians exhibited a greater frequency of single-rooted and single-canal compared to Caucasians.
5. The prevalence of single-rooted PMs was more prevalent in females, while double-rooted PMs were more frequently observed in males.
6. The root and canal morphology of PMs exhibited universal symmetry.
7. Acquiring proficiency in comprehending the RMCC system of PMs is imperative for clinicians to optimize the effectiveness of both surgical and nonsurgical dental interventions.

### Electronic supplementary material

Below is the link to the electronic supplementary material.

Supplementary Material 1

## Data Availability

All data generated or analysed during this study are included in this published article [and itssupplementary information files].
