# Supplementary material for: Systematic review and meta-analysis of root morphology and canal configuration of permanent premolars using cone-beam computed tomography
Source: BMC Oral Health. 2024 Jun 4;24:656. doi: 10.1186/s12903-024-04419-y (PMC11149329; doi:10.1186/s12903-024-04419-y)
Supplement: Supplementary file 1 — Supplementary Material 1 [file 12903_2024_4419_MOESM1_ESM.docx]

Supplementary Table 1. Alphabetically sorted English abbreviations.

| Abbreviations | |
| --- | --- |
| CBCT | cone-beam computed tomography |
| CI | confidence interval |
| MeSH | Medical Subject Heading |
| OR | odds ratios |
| PMs | premolars |
| RCT | root canal treatment |
| RMCC | root morphology and canal configuration |

Supplementary Table 2. Search strategies for EMBase, Cochrane and Web of Science.

| Search | Query |
| --- | --- |
| Search strategy for EMBase |  |
| #1 | exp premolar/ or bicuspid/ |
| #2 | (bicuspid).tw. |
| #3 | (bicuspid* or premolar*).tw. |
| #4 | 2 or 3 |
| #5 | exp dental pulp cavity/ |
| #6 | (dental pulp cavity).mp. |
| #7 | (cavit* or chamber* or canal*).mp. |
| #8 | 6 or 7 |
| #9 | exp cone-beam computed tomography/ |
| #10 | (cone-beam computed tomography).mp. |
| #11 | (CT or cone beam computer assisted).mp. |
| #12 | 10 or 11 |
| #13 | 4 and 8 and 12 |
| Search strategy for Cochrane |  |
| #1 | MeSH descriptor: [bicuspid] explode all trees |
| #2 | ‘bicuspid*’:ti,ab,kw OR ‘premolar*’:ti,ab,kw |
| #3 | #1 OR #2 |
| #4 | dental pulp cavity:pt |
| #5 | ' cavit*':ti,ab,kw OR 'chamber*':ti,ab,kw OR ' canal*’:ti,ab,kw |
| #6 | #4 OR #5 |
| #7 | cone-beam computed tomography:pt |
| #8 | ' CT':ti,ab,kw OR ' cone beam computer assisted':ti,ab,kw |
| #9 | #7 OR #8 |
| #10 | #3 AND #6 AND #9 |
| Search strategy for Web of Science | (bicuspid* OR premolar*) AND (dental pulp cavity OR cavit* OR chamber* OR canal*) AND (cone-beam computed tomography OR CT OR cone beam computer assisted) |

Supplementary Table 3. Summary of the number of roots and canals.

| Studies | Year | Population | N1 | 1 Root | 2 Roots | 3 Roots | N2 | 1 Canal | 2 Canals | 3 Canals |
| --- | --- | --- | --- | --- | --- | --- | --- | --- | --- | --- |
| Maxillary PM1s | | | | | | | | | | |
| Erkan, E., et al.[44] | 2023 | Turkish | 539 | 275  (51.0%) | 264  (49.0%) | 0  (0.0%) | 539 | 111  (20.6%) | 428  (79.4%) | 0  (0.0%) |
| Aljawhar, A M et al.[45] | 2023 | Iraqi | 572 | 273  (47.7%) | 292  (51.1%) | 7  (1.2%) |  |  |  |  |
| Mirah, M.A., et al.[46] | 2023 | Saudi | 613 | 102  (16.6%) | 505  (82.4%) | 6  (1.0%) |  |  |  |  |
| Al, Y.R., et al.[47] | 2023 | Saudi | 65 | 24  (36.9%) | 41  (63.1) | 0  (0.0%) |  |  |  |  |
| Khanna, S et al.[48] | 2023 | Gujarati | 137 | 45  (32.8%) | 92  (67.2%) | 0  (0.0%) | 137 | 34  (24.8%) | 103  (75.2%) | 0  (0.0%) |
| Shah, S.A.[49] | 2023 | Pakistan | 266 | 247  (92.9%) | 19  (7.1%) | 0  (0.0%) | 266 | 46  (17.3%) | 219  (82.3%) | 1  (0.4%) |
| Aguilera, J., et al.[50] | 2022 | Chilean | 121 | 86  (71.1%) | 35  (28.9%) | 0  (0.0%) | 121 | 37  (30.6%) | 84  (69.4%) | 0  (0.0%) |
| Iqbal, A., et al.[51] | 2022 | Saudi Arabian | 346 | 92  (26.6%) | 252  (72.8%) | 2  (0.6%) |  |  |  |  |
| Gündüz, H. et al.[23] | 2022 | Turkish | 966 | 301  (31.2%) | 623  (64.5%) | 42  (4.3%) | 966 | 77  (8.0%) | 847  (87.7%) | 42  (4.3%) |
| Diab, H., et al.[52] | 2022 | Qatari | 442 | 119  (26.9%) | 312  (70.6%) | 11  (2.5%) | 442 | 60  (13.6%) | 371  (83.9%) | 11  (2.5%) |
| Kartik SN et al.[53] | 2022 | Indian Dravidian | 400 | 116  (29.0%) | 284  (71.0%) | 0  (0.0%) |  |  |  |  |
| Alnaqbi, H.S.Y., et al.[54] | 2022 | UAE | 54 | 7  (13.0%) | 45  (83.3%) | 2  (3.7%) | 54 | 0  (0.0%) | 52  (96.3%) | 2  (3.7%) |
| Alnaqbi, H.S.Y., et al.[54] | 2022 | South Asian | 53 | 2  (3.8%) | 51  (96.2%) | 0  (0.0%) | 53 | 0  (0.0%) | 53  (100.0%) | 0  (0.0%) |
| Olczak et al.[55] | 2022 | Poland | 350 | 99  (28.3%) | 242  (69.1%) | 9  (2.6%) |  |  |  |  |
| Fournier et al.[56] | 2022 | French | 173 | 93  (53.8%) | 71  (41.0%) | 9  (5.2%) |  |  |  |  |
| Al-Zubaidi et al.[39] | 2021 | Saudi | 500 | 199  (39.8%) | 293  (58.6%) | 8  (1.6%) |  |  |  |  |
| Liu et al.[36] | 2021 | Chinese | 880 | 618  (70.2%) | 258  (29.3%) | 4  (0.45%) |  |  |  |  |
| Mashyakhy[57] | 2021 | Saudi | 351 | 143  (40.7%) | 202  (57.5%) | 6  (1.7%) | 350 | 13  (3.7%) | 327  (93.4%) | 9  (2.6%) |
| Haider, I., et al.[58] | 2021 | Pakistan (Lahore) | 150 | 60  (40.0%) | 90  (60.0%) | 0  (0.0%) | 150 | 2  (1.3%) | 141  (94.0%) | 6  (4.0%) |
| Yoza T et al.[59] | 2021 | Japan | 125 | 101  (80.8%) | 22  (17.6%) | 2  (1.6%) | 125 | 42  (33.6%) | 82  (65.6%) | 1  (0.8%) |
| Monardes, H., et al.[60] | 2021 | Chilean | 306 | 132  (43.1%) | 157  (51.3%) | 17  (5.6%) | 306 | 34  (11.1%) | 255  (83.3%) | 17  (5.6%) |
| Nikkerdar, N., et al.[61] | 2020 | Iranian | 125 | 94  (75.2%) | 31  (24.8%) | 0  (0.0%) |  |  |  |  |
| Asheghi, B., et al.[62] | 2020 | Iranian | 462 | 232  (50.2%) | 222  (48.1%) | 8  (1.7%) |  |  |  |  |
| Buchanan, G. D., et al.[37] | 2020 | South African | 316 | 139  (44.0%) | 171  (54.1%) | 6  (1.9%) |  |  |  |  |
| Kfir, A., et al.[63] | 2020 | Israeli | 400 | 143  (35.8%) | 245  (61.3%) | 12  (3.0%) | 400 | 8  (2.0%) | 380  (95.0%) | 12  (3.0%) |
| Wu, D., et al.[64] | 2020 | Chinese | 1268 | 855  (67.4%) | 406  (32.0%) | 7  (0.6%) |  |  |  |  |
| de Lima, C. O., et al.[65] | 2019 | Brazil | 496 | 90  (18.1%) | 398  (80.2%) | 8  (1.6%) |  |  |  |  |
| Maghfuri, S., et al.[66] | 2019 | Saudi | 100 | 36  (36.0%) | 61  (61.0%) | 3  (3.0%) |  |  |  |  |
| Pan, J. Y. Y., et al.[67] | 2019 | Malaysian | 304 | 206  (67.8%) | 97  (31.9%) | 1  (0.3%) |  |  |  |  |
| Saber, S., et al.[68] | 2019 | Egyptian | 358 | 164  (45.8%) | 190  (53.1%) | 4  (1.1%) |  |  |  |  |
| Alqedairi, A., et al.[69] | 2018 | Saudi | 334 | 79  (23.7%) | 251  (75.1%) | 4  (1.2%) |  |  |  |  |
| Li, Y.H., et al.[41] | 2018 | Chinese | 1387 | 967  (69.7%) | 413  (29.8%) | 7  (0.5%) | 1387 | 163  (11.8%) | 1214  (87.5%) | 10  (1.0%) |
| Martins, J. N. R., et al.[70] | 2018 | Asian | 238 | 198  (83.2%) | 40  (16.8%) | 0  (0.0%) | 238 | 29  (12.2%) | 209  (87.8%) | 0  (0.0%) |
| Martins, J. N. R., et al.[70] | 2018 | White | 714 | 348  (48.7%) | 351  (49.2%) | 15  (2.1%) | 714 | 24  (3.4%) | 654  (91.6%) | 36  (10.3%) |
| Nazeer, M. R., et al.[71] | 2018 | Pakistani | 114 | 36  (31.6%) | 78  (68.4%) | 0  (0.0%) | 114 | 6  (5.3%) | 102  (89.5%) | 6  (16.7%) |
| Razumova, S., et al.[72] | 2018 | Moscow | 460 | 40  (8.7%) | 420  (91.3%) | 0  (0.0%) | 460 | 28  (6.1%) | 432  (93.9%) | 0  (0.0%) |
| Burklein, S., et al.[27] | 2017 | German | 644 | 234  (36.3%) | 402  (62.4%) | 8  (1.2%) | 644 | 62  (9.6%) | 569  (88.4%) | 13  (5.6%) |
| Martins, J. N. R., et al.[73] | 2017 | Caucasian | 690 | 336  (48.7%) | 339  (49.1%) | 15  (2.2%) |  |  |  |  |
| Shi, Z.-Y., et al.[74] | 2017 | Chinese | 521 | 317  (60.8%) | 197  (37.8%) | 7  (1.3%) |  |  |  |  |
| Celikten, B., et al.[75] | 2016 | Turkish Cypriot | 437 | 236  (54.0%) | 196  (44.9%) | 4  (0.9%) |  |  |  |  |
| Abella, F., et al.[76] | 2015 | Spanish | 430 | 198  (46.0%) | 221  (51.4%) | 11  (2.6%) |  |  |  |  |
| Bulut, D. G., et al.[77] | 2015 | Turkish | 511 | 144  (28.2%) | 362  (70.8%) | 5  (1.0%) |  |  |  |  |
| Estrela, C., et al.[78] | 2015 | Brazil | 100 | 32  (32.0%) | 66  (66.0%) | 2  (2.0%) | 100 | 6  (6.0%) | 88  (88.0%) | 6  (6.0%) |
| Felsypremila, G., et al.[35] | 2015 | Indian | 418 | 204  (48.8%) | 214  (51.2%) | 0  (0.0%) |  |  |  |  |
| Ok, E., et al.[79] | 2014 | Turkish |  |  |  |  | 1379 | 173  (12.5%) | 1189  (86.2%) | 17  (1.2%) |
| Tian, Y. Y., et al.[80] | 2012 | Chinese | 300 | 198  (66.0%) | 100  (33.3%) | 2  (0.7%) |  |  |  |  |
| Total |  |  | 18536 | 8660  (46.7%) | 9621  (51.9%) | 254  (1.4%) | 8945 | 955  (10.7%) | 7799  (87.2%) | 189  (2.1%) |
| Maxillary PM2s | | | | | | | | | | |
| Erkan, E., et al.[44] | 2023 | Turkish | 516 | 464  (89.9%) | 52  (10.1%) | 0  (0.0%) | 516 | 354  (68.6%) | 162  (31.4%) | 0  (0.0%) |
| Aljawhar, A M et al.[45] | 2023 | Iraqi | 544 | 478  (87.9%) | 62  (11.4%) | 4  (0.7%) |  |  |  |  |
| Mirah, M.A., et al.[46] | 2023 | Saudi | 566 | 378  (66.8%) | 186  (32.9%) | 2  (0.4%) |  |  |  |  |
| Al, Y.R., et al.[47] | 2023 | Saudi | 56 | 53  （94.6%） | 3  (5.4%) | 0  (0.0%) |  |  |  |  |
| Khanna, S et al.[48] | 2023 | Gujarati | 125 | 72  (57.6%) | 53  (42.4%) | 0  (0.0%) | 125 | 73  (58.4%) | 52  (41.6%) | 0  (0.0%) |
| Shah, S.A.[49] | 2023 | Pakistan | 266 | 155  (58.3%) | 110  (41.4%) | 1  (0.4%) | 266 | 167  (62.8%) | 99  (37.2%) | 0  (0.0%) |
| Chourasia, H.R., et al.[81] | 2023 | Saudi Arabian | 602 | 474  (78.7%) | 125  (20.8%) | 3  (0.5%) | 602 | 243  (40.4%) | 356  (59.1%) | 3  (0.5%) |
| Olczak, et al.[82] | 2023 | Poland | 324 | 288  （88.9%） | 36  （11.1%） | 0  (0.0%) |  |  |  |  |
| Iqbal, A., et al.[51] | 2022 | Saudi Arabian | 298 | 242  (81.2%) | 56  (18.8%) | 0  (0.0%) |  |  |  |  |
| Gündüz, H. et al.[23] | 2022 | Turkish | 952 | 736  (77.3%) | 213  (22.4%) | 3  (0.3%) | 952 | 480  (50.4%) | 469  (49.3%) | 3  (0.3%) |
| Diab, H., et al.[52] | 2022 | Qatari | 408 | 344  (84.3%) | 64  (15.7%) | 0  (0.0%) | 408 | 287  (70.3%) | 121  (29.7%) | 0  (0.0%) |
| Selivany, B.J. et al.[83] | 2022 | Iraqi Kurdistan | 300 | 268  (89.3%) | 32  (10.7%) | 0  (0.0%) | 300 | 201  (67.0%) | 99  (33.0%) | 0  (0.0%) |
| Alnaqbi, H.S.Y., et al.[54] | 2022 | UAE | 56 | 35  (62.5%) | 21  (37.5%) | 0  (0.0%) | 56 | 1  (1.8%) | 55  (98.2%) | 0  (0.0%) |
| Alnaqbi, H.S.Y., et al.[54] | 2022 | South Asian | 52 | 17  (32.7%) | 35  (67.3%) | 0  (0.0%) | 52 | 0  (0.0%) | 52  (100.0%) | 0  (0.0%) |
| Fournier et al.[56] | 2022 | French | 139 | 130  (93.5%) | 7  (5.0%) | 2  (1.4%) |  |  |  |  |
| Mashyakhy[57] | 2021 | Saudi | 359 | 316  (88.0%) | 43  (12.0%) | 0  (0.0%) | 359 | 137  (38.2%) | 219  (61.0%) | 3  (0.8%) |
| Al-Zubaidi et al.[39] | 2021 | Saudi | 500 | 416  (83.2%) | 79  (15.8%) | 5  (1.0%) |  |  |  |  |
| Monardes, H., et al.[60] | 2021 | Chilean | 286 | 266  (93.0%) | 20  (7.0%) | 0  (0.0%) | 286 | 182  (63.6%) | 104  (36.4%) | 0  (0.0%) |
| Yan, Y., et al.[84] | 2021 | western Chinese | 1118 | 1053  (94.2%) | 65  (5.8%) | 0  (0.0%) | 1118 | 616  (55.1%) | 500  (44.7%) | 2  (0.2%) |
| Nikkerdar, N., et al.[61] | 2020 | Iranian | 125 | 119  (95.2%) | 6  (4.8%) | 0  (0.0%) |  |  |  |  |
| Asheghi, B., et al.[62] | 2020 | Iranian | 400 | 364  (91.0%) | 34  (8.5%) | 2  (0.5%) |  |  |  |  |
| Buchanan, G. D., et al.[37] | 2020 | South African | 285 | 223  (78.2%) | 58  (20.4%) | 4  (1.4%) |  |  |  |  |
| de Lima, C. O., et al.[65] | 2019 | Brazil | 503 | 358  (71.2%) | 143  (28.4%) | 2  (0.4%) |  |  |  |  |
| Pan, J. Y. Y., et al.[67] | 2019 | Malaysian | 333 | 306  (91.9%) | 27  (8.1%) | 0  (0.0%) |  |  |  |  |
| Saber, S., et al.[68] | 2019 | Egyptian | 342 | 249  (72.8%) | 89  (26.0%) | 4  (1.2%) |  |  |  |  |
| Alqedairi, A., et al.[69] | 2018 | Saudi | 318 | 271  (85.2%) | 46  (14.5%) | 1  (0.3%) |  |  |  |  |
| Li, Y.H., et al.[41] | 2018 | Chinese | 1403 | 1350  (96.2%) | 53  (3.8%) | 0  (0.0%) | 1403 | 706  (50.3%) | 697  (49.7%) | 0  (0.0%) |
| Martins, J. N. R., et al.[70] | 2018 | Asian | 239 | 237  (99.2%) | 2  (0.8%) | 0  (0.0%) | 239 | 179  (74.9%) | 60  (25.1%) | 0  (0.0%) |
| Martins, J. N. R., et al.[70] | 2018 | White | 618 | 585  (94.7%) | 33  (5.3%) | 0  (0.0%) | 617 | 245  (39.7%) | 369  (59.8%) | 3  (0.5%) |
| Nazeer, M. R., et al.[71] | 2018 | Pakistani | 115 | 97  (84.3%) | 18  (15.7%) | 0  (0.0%) | 115 | 57  (49.6%) | 56  (48.7%) | 2  (1.7%) |
| Razumova, S., et al.[72] | 2018 | Moscow | 423 | 112  (26.5%) | 311  (73.5%) | 0  (0.0%) | 423 | 75  (17.7%) | 348  (82.3%) | 0  (0.0%) |
| Burklein, S., et al.[27] | 2017 | German | 512 | 423  (82.6%) | 87  (17.0%) | 2  (0.4%) | 512 | 221  (43.2%) | 288  (56.3%) | 3  (0.6%) |
| Martins, J. N. R., et al.[73] | 2017 | Caucasian | 591 | 558  (94.4%) | 33  (5.6%) | 0  (0.0%) |  |  |  |  |
| Shi, Z.-Y., et al.[74] | 2017 | Chinese | 517 | 478  (92.5%) | 39  (7.5%) | 0  (0.0%) |  |  |  |  |
| Celikten, B., et al.[75] | 2016 | Turkish Cypriot | 445 | 409  (91.9%) | 34  (7.6%) | 2  (0.4%) |  |  |  |  |
| Abella, F., et al.[76] | 2015 | Spanish | 374 | 310  (82.9%) | 58  (15.5%) | 6  (1.6%) |  |  |  |  |
| Bulut, D. G., et al.[77] | 2015 | Turkish | 476 | 391  (82.1%) | 85  (17.9%) | 0  (0.0%) |  |  |  |  |
| Estrela, C., et al.[78] | 2015 | Brazil | 100 | 83  (83.0%) | 17  (17.0%) | 0  (0.0%) | 100 | 25  (25.0%) | 73  (73.0%) | 2  (2.0%) |
| Felsypremila, G., et al.[35] | 2015 | Indian | 393 | 356  (90.6%) | 37  (9.4%) | 0  (0.0%) | 356 | 196  (55.1%) | 160  (44.9%) | 0  (0.0%) |
| Ok, E., et al.[79] | 2014 | Turkish |  |  |  |  | 1301 | 776  (59.6%) | 521  (40.0%) | 4  (0.3%) |
| Yang, L., et al.[85] | 2014 | Chinese | 392 | 339  (86.5%) | 53  (13.5%) | 0  (0.0%) | 392 | 178  (45.4%) | 213  (54.3%) | 1  (0.3%) |
| Total |  |  | 16371 | 13803  (84.3%) | 2525  (15.4%) | 43  (0.3%) | 10498 | 5399  (51.4%) | 5073  (48.3%) | 26  (0.2%) |
| Mandibular PM1s | | | | | | | | | | |
| Erkan, E., et al.[44] | 2023 | Turkish | 814 | 778  (95.6%) | 36  (4.4%) | 0  (0.0%) | 814 | 708  (87.0%) | 106  (13.0%) | 0  (0.0%) |
| Mirah, M.A., et al.[46] | 2023 | Saudi | 663 | 562  (84.8%) | 99  (14.9%) | 2  (0.3%) |  |  |  |  |
| Al, Y.R., et al.[47] | 2023 | Saudi | 32 | 32  (100%) | 0  (0.0%) | 0  (0.0%) |  |  |  |  |
| Khanna, S et al.[48] | 2023 | Gujarati | 134 | 128  (95.5%) | 6  (4.5%) | 0  (0.0%) | 134 | 116  (86.6%) | 18  (13.4%) | 0  (0.0%) |
| Rae O et al.[86] | 2023 | Melbourne | 1576 | 1558  (98.9%) | 17  (1.1%) | 1  (0.1%) |  |  |  |  |
| Iqbal, A., et al.[51] | 2022 | Saudi Arabian | 412 | 398  (96.6%) | 14  (3.4%) | 0  (0.0%) |  |  |  |  |
| Gündüz, H. et al.[23] | 2022 | Turkish | 988 | 888  (89.9%) | 96  (9.7%) | 4  (0.4%) | 988 | 760  (76.9%) | 224  (22.7%) | 4  (0.4%) |
| Mashyakhy, M., et al.[87] | 2022 | Saudi | 397 | 395  (99.5%) | 2  (0.5%) | 0  (0.0%) | 397 | 276  (69.5%) | 117  (29.5%) | 4  (1.0%) |
| Buchanan, G.D., et al.[24] | 2022 | Black South African | 386 | 378  (97.9%) | 7  (1.8%) | 1  (0.3%) |  |  |  |  |
| Thanaruengrong et al.[88] | 2021 | Thai | 621 | 609  (98.1%) | 10  (1.6%) | 2  (0.3%) |  |  |  |  |
| Algarni, Y. A., et al.[89] | 2021 | Saudi Arabian | 219 | 198  (90.4%) | 18  (8.2%) | 0  (0.0%) | 432 | 308  (71.3%) | 103  (23.8%) | 21  (4.9%) |
| Hasheminia, S.M., et al.[25] | 2021 | Iranian | 389 | 345  (88.7%) | 41  (10.5%) | 3  (0.8%) |  |  |  |  |
| Arayasantiparb, R. et al.[90] | 2021 | Thai | 349 | 329  (94.3%) | 17  (4.9%) | 3  (0.9%) |  |  |  |  |
| Alfonso-Rodriguez, C.A., et al.[91] | 2021 | Colombian |  |  |  |  | 100 | 94  (94.0%) | 6  (6.0%) | 0  (0.0%) |
| Mishra, S., et al.[92] | 2021 | Delhi-NCR | 216 | 205  (94.9%) | 11  (5.1%) | 0  (0.0%) | 216 | 86  (39.8%) | 129  (59.7%) | 1  (0.5%) |
| Alam, F., et al.[93] | 2020 | Saudi | 752 | 608  (80.9%) | 144  (19.1%) | 0  (0.0%) | 752 | 523  (69.5%) | 229  (30.5%) | 0  (0.0%) |
| Alenezi, D.J., et al.[94] | 2020 | Kuwaiti | 245 | 181  (73.9%) | 61  (24.9%) | 3  (1.2%) |  |  |  |  |
| Wu, D., et al.[64] | 2020 | Chinese | 1296 | 1280  (98.8%) | 16  (1.2%) | 0  (0.0%) |  |  |  |  |
| Alfawaz, H., et al.[26] | 2019 | Saudi | 391 | 377  (96.4%) | 12  (3.1%) | 2  (0.5%) |  |  |  |  |
| Corbella, S., et al.[95] | 2019 | Caucasians | 97 | 92  (94.8%) | 5  (5.2%) | 0  (0.0%) |  |  |  |  |
| Jang, Y. E., et al.[96] | 2019 | Korean | 971 | 951  (97.9%) | 20  (2.1%) | 0  (0.0%) |  |  |  |  |
| Pan, J. Y. Y., et al.[67] | 2019 | Malaysian | 359 | 353  (98.3%) | 6  (1.7%) | 0  (0.0%) |  |  |  |  |
| Martins, J. N. R., et al.[70] | 2018 | Asian | 238 | 238  (100.0%) | 0  (0.0%) | 0  (0.0%) | 238 | 206  (86.6%) | 31  (13.0%) | 1  (0.4%) |
| Martins, J. N. R., et al.[70] | 2018 | White | 1089 | 1087  (99.8%) | 2  (0.2%) | 0  (0.0%) | 1089 | 846  (77.7%) | 237  (21.8%) | 6  (0.6%) |
| Pedemonte, E., et al.[97] | 2018 | Belgium | 101 | 101  (100.0%) | 0  (0.0%) | 0  (0.0%) | 101 | 84  (83.2%) | 17  (16.8%) | 0  (0.0%) |
| Pedemonte, E., et al.[97] | 2018 | Chilean | 100 | 94  (94.0%) | 6  (6.0%) | 0  (0.0%) | 100 | 69  (69.0%) | 29  (29.0%) | 2  (2.0%) |
| Razumova, S., et al.[72] | 2018 | Moscow | 490 | 490  (100.0%) | 0  (0.0%) | 0  (0.0%) | 490 | 437  (89.2%) | 53  (10.8%) | 0  (0.0%) |
| Vega-Lizama et al.[98] | 2018 | Yucatecan | 105 | 102  (97.1%) | 3  (2.9%) | 0  (0.0%) | 102 | 54  (52.9%) | 35  (34.3%) | 10  (9.8%) |
| Burklein, S., et al.[27] | 2017 | German | 1044 | 954  (91.4%) | 90  (8.6%) | 0  (0.0%) | 1044 | 813  (77.9%) | 229  (21.9%) | 2  (0.2%) |
| Hajihassani, N., et al.[99] | 2017 | Iranian | 124 | 119  (96.0%) | 5  (4.0%) | 0  (0.0%) |  |  |  |  |
| Martins, J. N. R., et al.[73] | 2017 | Caucasian | 1054 | 1052  (99.8%) | 2  (0.2%) | 0  (0.0%) |  |  |  |  |
| Bulut, D. G., et al.[77] | 2015 | Turkish | 604 | 581  (96.2%) | 23  (3.8%) | 0  (0.0%) |  |  |  |  |
| Estrela, C., et al.[78] | 2015 | Brazil | 100 | 99  (99.0%) | 1  (1.0%) | 0  (0.0%) | 100 | 70  (70.0%) | 29  (29.0%) | 1  (1.0%) |
| Felsypremila, G., et al.[35] | 2015 | Indian | 447 | 438  (98.0%) | 9  (2.0%) | 0  (0.0%) | 438 | 414  (94.5%) | 24  (5.5%) | 0  (0.0%) |
| Kazemipoor, M., et al.[100] | 2015 | Iranian | 914 | 790  (86.4%) | 124  (13.6%) | 0  (0.0%) | 914 | 622  (68.1%) | 292  (31.9%) | 0  (0.0%) |
| Kazemipoor, M., et al.[101] | 2015 | Iranian | 460 | 394  (85.7%) | 66  (14.3%) | 0  (0.0%) | 460 | 294  (63.9%) | 166  (36.1%) | 0  (0.0%) |
| Huang, Y. D., et al.[102] | 2014 | Taiwanese | 300 | 246  (82.0%) | 51  (17.0%) | 3  (1.0%) | 300 | 197  (65.7%) | 100  (33.3%) | 3  (1.0%) |
| Llena, C., et al.[103] | 2014 | Spanish |  |  |  |  | 73 | 57  (78.1%) | 15  (20.5%) | 1  (1.4%) |
| Ok, E., et al.[79] | 2014 | Turkish |  |  |  |  | 1471 | 1375  (93.5%) | 95  (6.5%) | 1  (0.1%) |
| Yang, H., et al.[104] | 2013 | Chinese |  |  |  |  | 435 | 335  (77.0%) | 97  (22.3%) | 3  (0.7%) |
| Yu, X., et al.[105] | 2012 | Chinese | 178 | 174  (97.8%) | 4  (2.2%) | 0  (0.0%) | 176 | 155  (88.1%) | 20  (11.4%) | 1  (0.6%) |
| Total |  |  | 18655 | 17604  (94.4%) | 1024  (5.5%) | 24  (0.1%) | 11364 | 8899  (78.3%) | 2401  (21.1%) | 61  (0.5%) |
| Mandibular PM2s | | | | | | | | | | |
| Erkan, E., et al.[44] | 2023 | Turkish | 701 | 696  (99.3%) | 5  (0.7%) | 0  (0.0%) | 701 | 679  (96.9%) | 22  (3.1%) | 0  (0.0%) |
| Mirah, M.A., et al.[46] | 2023 | Saudi | 600 | 576  (96.0%) | 24  (4.0%) | 0  (0.0%) |  |  |  |  |
| Al, Y.R., et al.[47] | 2023 | Saudi | 59 | 59  (100%) | 0  (0.0%) | 0  (0.0%) |  |  |  |  |
| Khanna, S et al.[48] | 2023 | Gujarati | 134 | 134  (100%) | 0  (0.0%) | 0  (0.0%) | 134 | 131  (97.8%) | 3  (2.2%) | 0  (0.0%) |
| Rae O et al.[86] | 2023 | Melbourne | 1424 | 1407  (98.8%) | 17  (1.2%) | 0  (0.0%) |  |  |  |  |
| Iqbal, A., et al.[51] | 2022 | Saudi Arabian | 387 | 379  (97.9%) | 8  (2.1%) | 0  (0.0%) |  |  |  |  |
| Gündüz, H. et al.[23] | 2022 | Turkish | 974 | 958  (98.4%) | 13  (1.3%) | 3  (0.3%) | 988 | 934  (94.5%) | 35  (3.5%) | 5  (0.5%) |
| Mashyakhy, M., et al.[87] | 2022 | Saudi | 379 | 379  (100.0%) | 0  (0.0%) | 0  (0.0%) | 379 | 367  (96.8%) | 8  (2.1%) | 4  (1.1%) |
| Buchanan, G.D., et al.[24] | 2022 | Black South African | 386 | 372  (96.4%) | 9  (2.3%) | 5  (1.3%) |  |  |  |  |
| Alghamdi, et al.[40] | 2022 | Saudi | 2400 | 2360  (98.3%) | 40  (1.7%) | 0  (0.0%) |  |  |  |  |
| Thanaruengrong et al.[88] | 2021 | Thai | 538 | 537  (99.8%) | 1  (0.2%) | 0  (0.0%) |  |  |  |  |
| Arayasantiparb, R. et al.[90] | 2021 | Thai | 416 | 416  (100.0%) | 0  (0.0%) | 0  (0.0%) |  |  |  |  |
| Hasheminia, S.M., et al.[25] | 2021 | Iranian | 384 | 341  (88.8%) | 35  (9.1%) | 8  (2.1%) |  |  |  |  |
| Mishra, S., et al.[92] | 2021 | Delhi-NCR | 216 | 212  (98.1%) | 4  (1.9%) | 0  (0.0%) | 216 | 126  (58.3%) | 90  (41.7%) | 0  (0.0%) |
| Alam, F., et al.[93] | 2020 | Saudi | 752 | 664  (88.3%) | 88  (11.7%) | 0  (0.0%) | 752 | 540  (71.8%) | 212  (28.2%) | 0  (0.0%) |
| Alenezi, D.J., et al.[94] | 2020 | Kuwaiti | 231 | 183  (79.2%) | 48  (20.8%) | 0  (0.0%) |  |  |  |  |
| Alfawaz, H., et al.[26] | 2019 | Saudi | 343 | 328  (95.6%) | 13  (3.8%) | 2  (0.6%) |  |  |  |  |
| Corbella, S., et al.[95] | 2019 | Caucasians | 88 | 85  (96.6%) | 3  (3.4%) | 0  (0.0%) |  |  |  |  |
| Jang, Y. E., et al.[96] | 2019 | Korean | 997 | 997  (100.0%) | 0  (0.0%) | 0  (0.0%) |  |  |  |  |
| Pan, J. Y. Y., et al.[67] | 2019 | Malaysian | 399 | 399  (100.0%) | 0  (0.0%) | 0  (0.0%) |  |  |  |  |
| Martins, J. N. R., et al.[70] | 2018 | Asian | 236 | 236  (100.0%) | 0  (0.0%) | 0  (0.0%) | 236 | 235  (99.6%) | 1  (0.4%) | 0  (0.0%) |
| Martins, J. N. R., et al.[70] | 2018 | White | 858 | 857  (99.9%) | 1  (0.1%) | 0  (0.0%) | 858 | 821  (95.7%) | 34  (4.0%) | 3  (0.3%) |
| Pedemonte, E., et al.[97] | 2018 | Belgium | 101 | 99  (98.0%) | 2  (2.0%) | 0  (0.0%) | 173 | 92  (53.2%) | 81  (46.8%) | 0  (0.0%) |
| Pedemonte, E., et al.[97] | 2018 | Chilean | 100 | 99  (99.0%) | 1  (1.0%) | 0  (0.0%) | 100 | 95  (95.0%) | 4  (4.0%) | 1  (1.0%) |
| Razumova, S., et al.[72] | 2018 | Moscow | 443 | 442  (99.8%) | 1  (0.2%) | 0  (0.0%) | 443 | 399  (90.1%) | 44  (9.9%) | 0  (0.0%) |
| Burklein, S., et al.[27] | 2017 | German | 871 | 859  (98.6%) | 11  (1.3%) | 1  (0.1%) | 870 | 836  (96.1%) | 31  (3.6%) | 3  (0.3%) |
| Hajihassani, N., et al.[99] | 2017 | Iranian | 100 | 100  (100.0%) | 0  (0.0%) | 0  (0.0%) |  |  |  |  |
| Martins, J. N. R., et al.[73] | 2017 | Caucasian | 833 | 832  (99.9%) | 1  (0.1%) | 0  (0.0%) |  |  |  |  |
| Bulut, D. G., et al.[77] | 2015 | Turkish | 549 | 543  (98.9%) | 6  (1.1%) | 0  (0.0%) |  |  |  |  |
| Estrela, C., et al.[78] | 2015 | Brazil | 100 | 100  (100.0%) | 0  (0.0%) | 0  (0.0%) | 100 | 97  (97.0%) | 3  (3.0%) | 0  (0.0%) |
| Felsypremila, G., et al.[35] | 2015 | Indian | 398 | 398  (100.0%) | 0  (0.0%) | 0  (0.0%) | 398 | 397  (99.7%) | 1  (0.3%) | 0  (0.0%) |
| Kazemipoor, M., et al.[100] | 2015 | Iranian | 914 | 868  (95.0%) | 46  (5.0%) | 0  (0.0%) | 914 | 737  (80.6%) | 177  (19.4%) | 0  (0.0%) |
| Kazemipoor, M., et al.[101] | 2015 | Iranian | 450 | 426  (94.7%) | 24  (5.3%) | 0  (0.0%) | 460 | 360  (78.3%) | 100  (21.7%) | 0  (0.0%) |
| Llena, C., et al.[103] | 2014 | Spanish |  |  |  |  | 53 | 48  (90.6%) | 5  (9.4%) | 0  (0.0%) |
| Ok, E., et al.[79] | 2014 | Turkish |  |  |  |  | 1345 | 1325  (98.5%) | 17  (1.3%) | 3  (0.2%) |
| Yu, X., et al.[105] | 2012 | Chinese | 178 | 178  (100.0%) | 0  (0.0%) | 0  (0.0%) | 177 | 173  (97.7%) | 4  (2.3%) | 0  (0.0%) |
| Total |  |  | 17939 | 17519  (97.7%) | 401  (2.2%) | 19  (0.1%) | 9297 | 8392  (90.3%) | 872  (9.4%) | 19  (0.2%) |

Supplementary Table 4. Summary of the root canal configuration.

| Studies | Year | Population | N3 | Vertucci I | Vertucci II | Vertucci III | Vertucci IV | Vertucci V | Vertucci VI | Vertucci VII | Vertucci VIII | Others | C-shaped |
| --- | --- | --- | --- | --- | --- | --- | --- | --- | --- | --- | --- | --- | --- |
| Maxillary PM1s | | | | | | | | | | | | | |
| Erkan, E., et al.[44] | 2023 | Turkish | 539 | 55  (10.2%) | 36  (6.7%) | 21  (3.9%) | 414  (77.0%) | 2  (0.4%) | 2  (0.4%) | 0  (0.0%) | 8  (1.5%) | 0  (0.0%) |  |
| Aljawhar, A M et al.[45] | 2023 | Iraqi | 572 | 32  (5.6%) | 2  (0.3%) | 79  (13.8%) | 267  (46.7%) | 95  (16.6%) | 0  (0.0%) | 48  (8.4%) | 14  (2.4%) | 35  (6.1%) |  |
| Mirah, M.A., et al.[46] | 2023 | Saudi | 613 | 15  (2.4%) | 78  (12.7%) | 0  (0.0%) | 511  (83.4%) | 3  (0.5%) | 0  (0.0%) | 0  (0.0%) | 6  (1.0%) | 0  (0.0%) |  |
| Khanna, S et al.[48] | 2023 | Gujarati | 137 | 11  (8.0%) | 12  (8.8%) | 11  (8.0%) | 90  (65.7%) | 4  (2.9%) | 6  (4.4%) | 0  (0.0%) | 0  (0.0%) | 3  (2.2%) |  |
| Shah, S.A.[49] | 2023 | Pakistan | 266 | 121  (45.5%) | 9  (3.4%) | 29  (10.9%) | 69  (25.9%) | 32  (12.0%) | 1  (0.4%) | 4  (1.5%) | 1  (0.4%) | 0  (0.0%) |  |
| Aguilera, J., et al.[50] | 2022 | Chilean | 121 | 38  (31.4%) | 20  (16.5%) | 12  (9.9%) | 50  (41.3%) | 1  (0.8%) | 0  (0.0%) | 0  (0.0%) | 0  (0.0%) | 0  (0.0%) |  |
| Iqbal, A., et al.[51] | 2022 | Saudi Arabian | 346 | 201  (58.1%) | 134  (38.7%) | 4  (1.2%) | 4  (1.2%) | 3  (0.9%) | 0  (0.0%) | 0  (0.0%) | 0  (0.0%) | 0  (0.0%) |  |
| Gündüz, H. et al.[23] | 2022 | Turkish | 966 | 73  (7.6%) | 92  (9.5%) | 77  (8.0%) | 655  (67.8%) | 18  (1.9%) | 4  (0.4%) | 5  (0.5%) | 42  (4.3%) | 0  (0.0%) |  |
| Alnaqbi, H.S.Y., et al.[54] | 2022 | UAE | 54 | 1  (1.9%) | 3  (5.6%) | 3  (5.6%) | 14  (25.9%) | 28  (51.9%) | 2  (3.7%) | 1  (1.9%) | 0  (0.0%) | 2  (3.7%) |  |
| Alnaqbi, H.S.Y., et al.[54] | 2022 | South Asian | 53 | 1  (1.9%) | 1  (1.9%) | 0  (0.0%) | 5  (9.4%) | 31  (58.5%) | 4  (7.5%) | 4  (7.5%) | 0  (0.0%) | 7  (13.2%) |  |
| Olczak et al.[55] | 2022 | Poland | 350 | 6  (1.7%) | 30  (8.6%) | 9  (2.6%) | 275  (78.6%) | 18  (5.1%) | 0  (0.0%) | 2  (0.6%) | 10  (2.9%) | 0  (0.0%) |  |
| Lemos, M.C., et al.[106] | 2022 | Brazil | 310 | 11  (3.5%) | 61  (19.7%) | 1  (0.3%) | 229  (73.9%) | 1  (0.3%) | 0  (0.0%) | 0  (0.0%) | 7  (2.3%) | 0  (0.0%) |  |
| Fournier et al.[56] | 2022 | French | 177 | 87  (49.2%) | 24  (13.6%) | 4  (2.3%) | 50  (28.2%) | 9  (5.1%) | 3  (1.7%) | 0  (0.0%) | 0  (0.0%) | 0  (0.0%) |  |
| Al-Zubaidi et al.[39] | 2021 | Saudi | 500 | 26  (5.2%) | 164  (32.8%) | 3  (0.6%) | 289  (57.8%) | 10  (2%) | 0  (0.0%) | 0  (0.0%) | 8  (1.6%) | 0  (0.0%) |  |
| Liu et al.[36] | 2021 | Chinese | 880 | 245  (27.8%) | 181  (20.6%) | 9  (1.0%) | 390  (44.3%) | 29  (3.3%) | 13  (1.5%) | 9  (1.0%) | 4  (0.5%) | 0  (0.0%) |  |
| Mashyakhy[57] | 2021 | Saudi | 351 | 13  (3.7%) | 24  (6.8%) | 27  (7.7%) | 224  (63.8%) | 52  (14.8%) | 1  (0.3%) | 0  (0.0%) | 0  (0.0%) | 10  (2.8%) |  |
| Haider, I., et al.[58] | 2021 | Pakistan (Lahore) | 150 | 108  (72.0%) | 6  (4.0%) | 4  (2.7%) | 8  (5.3%) | 20  (13.3%) | 3  (2.0%) | 1  (0.7%) | 0  (0.0%) | 0  (0.0%) |  |
| Yoza T et al.[59] | 2021 | Japan | 125 | 32  (25.6%) | 34  (27.2%) | 4  (3.2%) | 47  (37.6%) | 6  (4.8%) | 0  (0.0%) | 0  (0.0%) | 1  (0.8%) | 1  (0.8%) |  |
| Nikkerdar, N., et al.[61] | 2020 | Iranian | 125 | 23  (18.4%) | 3  (2.4%) | 25  (20.0%) | 12  (9.6%) | 50  (40.0%) | 12  (9.6%) | 0  (0.0%) | 0  (0.0%) | 0  (0.0%) |  |
| Asheghi, B., et al.[62] | 2020 | Iranian | 462 | 41  (8.9%) | 70  (15.2%) | 4  (0.9%) | 331  (71.6%) | 6  (1.3%) | 1  (0.2%) | 0  (0.0%) | 9  (1.9%) | 0  (0.0%) |  |
| Buchanan, G. D., et al.[37] | 2020 | South African | 316 | 28  (8.9%) | 23  (7.3%) | 15  (4.7%) | 227  (71.8%) | 7  (2.2%) | 7  (2.2%) | 0  (0.0%) | 9  (2.8%) | 0  (0.0%) |  |
| Kfir, A., et al.[63] | 2020 | Israeli | 400 | 7  (1.8%) | 67  (16.8%) | 2  (0.5%) | 295  (73.8%) | 2  (0.5%) | 27  (6.8%) | 0  (0.0%) | 0  (0.0%) | 0  (0.0%) |  |
| Wu, D., et al.[64] | 2020 | Chinese | 1268 | 132  (10.4%) | 307  (24.2%) | 5  (0.4%) | 743  (58.6%) | 62  (4.9%) | 11  (0.9%) | 0  (0.0%) | 5  (0.4%) | 3  (0.2%) |  |
| de Lima, C. O., et al.[65] | 2019 | Brazil | 496 | 32  (6.5%) | 38  (7.7%) | 3  (0.6%) | 408  (82.3%) | 4  (0.8%) | 3  (0.6%) | 0  (0.0%) | 8  (1.6%) | 0  (0.0%) |  |
| Maghfuri, S., et al.[66] | 2019 | Saudi | 100 | 0  (0.0%) | 7  (7.0%) | 0  (0.0%) | 75  (75.0%) | 13  (13.0%) | 2  (2.0%) | 0  (0.0%) | 3  (3.0%) | 0  (0.0%) |  |
| Pan, J. Y. Y., et al.[67] | 2019 | Malaysian | 304 | 133  (43.8%) | 49  (16.1%) | 32  (10.5%) | 46  (15.1%) | 11  (3.6%) | 28  (9.2%) | 5  (1.6%) | 0  (0.0%) | 0  (0.0%) |  |
| Saber, S., et al.[68] | 2019 | Egyptian | 358 | 4  (1.1%) | 56  (15.6%) | 5  (1.4%) | 262  (73.2%) | 5  (1.4%) | 14  (3.9%) | 6  (1.7%) | 5  (1.4%) | 1  (0.3%) |  |
| Alqedairi, A., et al.[69] | 2018 | Saudi | 334 | 36  (10.8%) | 28  (8.4%) | 6  (1.8%) | 236  (70.7%) | 13  (3.9%) | 7  (2.1%) | 1  (0.3%) | 7  (2.1%) | 0  (0.0%) |  |
| Li, Y.H., et al.[41] | 2018 | Chinese | 1387 | 163  (11.8%) | 204  (14.7%) | 223  (16.1%) | 592  (42.7%) | 168  (12.1%) | 9  (0.6%) | 10  (0.7%) | 10  (0.7%) | 8  (0.6%) |  |
| Martins, J. N. R., et al.[70] | 2018 | Asian | 238 | 29  (12.2%) | 54  (22.7%) | 10  (4.2%) | 131  (55.0%) | 14  (5.9%) | 0  (0.0%) | 0  (0.0%) | 0  (0.0%) | 0  (0.0%) |  |
| Martins, J. N. R., et al.[70] | 2018 | White | 714 | 24  (3.4%) | 122  (17.1%) | 2  (0.3%) | 487  (68.2%) | 7  (1.0%) | 33  (4.6%) | 0  (0.0%) | 5  (0.7%) | 34  (4.8%) |  |
| Nazeer, M. R., et al.[71] | 2018 | Pakistani | 187 | 127  (67.9%) | 24  (12.8%) | 14  (7.5%) | 0  (0.0%) | 7  (3.7%) | 6  (3.2%) | 0  (0.0%) | 1  (0.5%) | 8  (4.3%) |  |
| Burklein, S., et al.[27] | 2017 | German | 652 | 25  (3.8%) | 42  (6.4%) | 0  (0.0%) | 441  (67.6%) | 51  (7.8%) | 79  (12.1%) | 1  (0.2%) | 13  (2.0%) | 0  (0.0%) |  |
| Martins, J. N. R., et al.[73] | 2017 | Caucasian | 690 | 22  (3.2%) | 119  (17.2%) | 2  (0.3%) | 469  (68.0%) | 6  (0.9%) | 33  (4.8%) | 0  (0.0%) | 5  (0.7%) | 34  (4.9%) |  |
| Shi, Z.-Y., et al.[74] | 2017 | Chinese | 521 | 21  (4.0%) | 150  (28.8%) | 0  (0.0%) | 271  (52.0%) | 7  (1.3%) | 47  (9.0%) | 1  (0.2%) | 10  (1.9%) | 14  (2.7%) |  |
| Abella, F., et al.[76] | 2015 | Spanish | 430 | 108  (25.1%) | 44  (10.2%) | 19  (4.4%) | 227  (52.8%) | 8  (1.9%) | 7  (1.6%) | 6  (1.4%) | 11  (2.6%) | 0  (0.0%) |  |
| Bulut, D. G., et al.[77] | 2015 | Turkish | 1252 | 784  (62.6%) | 427  (34.1%) | 10  (0.8%) | 24  (1.9%) | 7  (0.6%) | 0  (0.0%) | 0  (0.0%) | 0  (0.0%) | 0  (0.0%) |  |
| Felsypremila, G., et al.[35] | 2015 | Indian | 204 | 33  (15.9%) | 53  (26.1%) | 0  (0.0%) | 118  (58.0%) | 0  (0.0%) | 0  (0.0%) | 0  (0.0%) | 0  (0.0%) | 0  (0.0%) |  |
| Ok, E., et al.[79] | 2014 | Turkish | 1379 | 132  (9.6%) | 89  (6.5%) | 19  (1.4%) | 1061  (76.9%) | 63  (4.6%) | 1  (0.1%) | 0  (0.0%) | 14  (1.0%) | 0  (0.0%) |  |
| Tian, Y. Y., et al.[80] | 2012 | Chinese | 300 | 43  (14.3%) | 70  (23.3%) | 13  (4.3%) | 153  (51.0%) | 10  (3.3%) | 7  (2.3%) | 2  (0.7%) | 2  (0.7%) | 0  (0.0%) |  |
| Total |  |  | 18627 | 3023  (16.2%) | 2957  (15.9%) | 706  (3.8%) | 10200  (54.8%) | 883  (4.7%) | 373  (2.0%) | 106  (0.6%) | 218  (1.2%) | 160  (0.9%) |  |
| Maxillary PM2s | | | | | | | | | | | | | |
| Olczak, et al.[82] | 2023 | Poland | 324 | 193  (59.6%) | 30  (9.3%) | 20  (6.2%) | 51  (15.7%) | 23  (7.1%) | 3  (0.9%) | 3  (0.9%) | 1  (0.3%) | 0  (0.0%) |  |
| Erkan, E., et al.[44] | 2023 | Turkish | 516 | 296  (57.4%) | 30  (5.8%) | 28  (5.4%) | 149  (28.9%) | 10  (1.9%) | 2  (0.4%) | 0  (0.0%) | 1  (0.2%) | 0  (0.0%) |  |
| Aljawhar, A M et al.[45] | 2023 | Iraqi | 544 | 199 (36.6%) | 2  (0.4%) | 119 (21.9%) | 52  (9.6%) | 77 (14.2%) | 0  (0.0%) | 63  (11.6%) | 6  (1.1%) | 26  (4.8%) |  |
| Mirah, M.A., et al.[46] | 2023 | Saudi | 566 | 218  (38.5%) | 92  (16.3%) | 9  (1.6%) | 227  (40.1%) | 17  (3.0%) | 0  (0.0%) | 1  (0.2%) | 2  (0.4%) | 0  (0.0%) |  |
| Khanna, S et al.[48] | 2023 | Gujarati | 125 | 44  (35.2%) | 14  (11.2%) | 15  (12.0%) | 40  (32.0%) | 5  (4.0%) | 7  (5.6%) | 0  (0.0%) | 0  (0.0%) | 0  (0.0%) |  |
| Shah, S.A.[49] | 2023 | Pakistan | 266 | 171  (64.3%) | 11  (4.1%) | 41  (15.4%) | 12  (4.5%) | 23  (8.6%) | 2  (0.8%) | 6  (2.3%) | 0  (0.0%) | 0  (0.0%) |  |
| Iqbal, A., et al.[51] | 2022 | Saudi Arabian | 298 | 211  (70.8%) | 47  (15.8%) | 12  (4.0%) | 18  (6.0%) | 6  (2.0%) | 4  (1.3%) | 0  (0.0%) | 0  (0.0%) | 0  (0.0%) |  |
| Gündüz, H. et al.[23] | 2022 | Turkish | 952 | 480  (50.4%) | 78  (8.2%) | 104  (10.9%) | 214  (22.5%) | 63  (6.6%) | 1  (0.1%) | 9  (0.9%) | 3  (0.3%) | 0  (0.0%) |  |
| Selivany, B.J. et al.[83] | 2022 | Iraqi Kurdistan | 300 | 207  (69.0%) | 33  (11.0%) | 6  (2.0%) | 15  (5.0%) | 37  (12.3%) | 2  (0.7%) | 0  (0.0%) | 0  (0.0%) | 0  (0.0%) |  |
| Alnaqbi, H.S.Y., et al.[54] | 2022 | UAE | 56 | 1  (1.8%) | 18  (32.1%) | 15  (26.8%) | 6  (10.7%) | 11  (19.6%) | 4  (7.1%) | 1  (1.8%) | 0  (0.0%) | 0  (0.0%) |  |
| Alnaqbi, H.S.Y., et al.[54] | 2022 | South Asian | 52 | 0  (0.0%) | 4  (7.7%) | 8  (15.4%) | 3  (5.8%) | 13  (25.0%) | 5  (9.6%) | 7  (13.5%) | 0  (0.0%) | 12  (23.1%) |  |
| Fournier et al.[56] | 2022 | French | 139 | 71  (51.1%) | 22  (15.8%) | 17  (12.2%) | 16  (11.5%) | 7  (5.0%) | 4  (2.9%) | 1  (0.7%) | 1  (0.7%) | 0  (0.0%) |  |
| Lemos, M.C., et al.[106] | 2022 | Brazil | 284 | 134  (47.2%) | 68  (23.9%) | 8  (2.8%) | 70  (24.6%) | 4  (1.4%) | 0  (0.0%) | 0  (0.0%) | 0  (0.0%) | 0  (0.0%) |  |
| Al-Zubaidi et al.[39] | 2021 | Saudi | 500 | 302  (60.4%) | 82  (16.4%) | 32  (6.4%) | 64  (12.8%) | 14  (2.8%) | 0  (0.0%) | 1  (0.2%) | 5  (1.0%) | 0  (0.0%) |  |
| Mashyakhy[57] | 2021 | Saudi | 359 | 137  (38.2%) | 39  (10.9%) | 55  (15.3%) | 69  (19.2%) | 44  (12.3%) | 4  (1.1%) | 8  (2.2%) | 0  (0.0%) | 3  (0.8%) |  |
| Fauzi et al.[107] | 2021 | chennai | 100 | 41  (41.0%) | 9  (9.0%) | 22  (22.0%) | 3  (3.0%) | 22  (22.0%) | 0  (0.0%) | 2  (2.0%) | 1  (1.0%) | 0  (0.0%) |  |
| Yan, Y., et al.[84] | 2021 | western Chinese | 1118 | 616  (55.1%) | 357  (31.9%) | 6  (0.5%) | 114  (10.2%) | 17  (1.5%) | 5  (0.4%) | 1  (0.1%) | 2  (0.2%) | 0  (0.0%) |  |
| Nikkerdar, N., et al.[61] | 2020 | Iranian | 125 | 57  (45.6%) | 12  (9.6%) | 35  (28.0%) | 5  (4.0%) | 16  (12.8%) | 0  (0.0%) | 0  (0.0%) | 0  (0.0%) | 0  (0.0%) |  |
| Asheghi, B., et al.[62] | 2020 | Iranian | 400 | 252  (63.0%) | 78  (19.5%) | 3  (0.8%) | 57  (14.3%) | 4  (1.0%) | 2  (0.5%) | 0  (0.0%) | 4  (1.0%) | 0  (0.0%) |  |
| Buchanan, G. D., et al.[37] | 2020 | South African | 285 | 107  (37.5%) | 34  (11.9%) | 15  (5.3%) | 96  (33.7%) | 21  (7.4%) | 4  (1.4%) | 2  (0.7%) | 6  (2.1%) | 0  (0.0%) |  |
| de Lima, C. O., et al.[65] | 2019 | Brazil | 503 | 251  (49.9%) | 47  (9.3%) | 11  (2.2%) | 164  (32.6%) | 20  (4.0%) | 4  (0.8%) | 4  (0.8%) | 2  (0.4%) | 0  (0.0%) |  |
| Pan, J. Y. Y., et al.[67] | 2019 | Malaysian | 333 | 204  (61.3%) | 60  (18.0%) | 31  (9.3%) | 6  (1.8%) | 21  (6.3%) | 10  (3.0%) | 1  (0.3%) | 0  (0.0%) | 0  (0.0%) |  |
| Saber, S., et al.[68] | 2019 | Egyptian | 342 | 55  (16.1%) | 76  (22.2%) | 6  (1.8%) | 152  (44.4%) | 10  (2.9%) | 14  (4.1%) | 25  (7.3%) | 4  (1.2%) | 0  (0.0%) |  |
| Alqedairi, A., et al.[69] | 2018 | Saudi | 318 | 157  (49.4%) | 82  (25.8%) | 16  (5.0%) | 37  (11.6%) | 18  (5.7%) | 5  (1.6%) | 0  (0.0%) | 3  (0.9%) | 0  (0.0%) |  |
| Li, Y.H., et al.[41] | 2018 | Chinese | 1403 | 706  (50.3%) | 146  (10.4%) | 336  (23.9%) | 83  (5.9%) | 112  (8.0%) | 4  (0.3%) | 6  (0.4%) | 0  (0.0%) | 10  (0.7%) |  |
| Martins, J. N. R., et al.[70] | 2018 | Asian | 239 | 179  (74.9%) | 36  (15.1%) | 2  (0.8%) | 18  (7.5%) | 4  (1.7%) | 0  (0.0%) | 0  (0.0%) | 0  (0.0%) | 0  (0.0%) |  |
| Martins, J. N. R., et al.[70] | 2018 | White | 618 | 246  (39.8%) | 177  (28.6%) | 13  (2.1%) | 106  (17.2%) | 29  (4.7%) | 40  (6.5%) | 0  (0.0%) | 0  (0.0%) | 7  (1.1%) |  |
| Nazeer, M. R., et al.[71] | 2018 | Pakistani | 133 | 71  (53.4%) | 18  (13.5%) | 8  (6.0%) | 4  (3.0%) | 6  (4.5%) | 17  (12.8%) | 0  (0.0%) | 9  (6.8%) | 0  (0.0%) |  |
| Burklein, S., et al.[27] | 2017 | German | 512 | 73  (14.3%) | 57  (11.1%) | 3  (0.6%) | 128  (25.0%) | 147  (28.7%) | 98  (19.1%) | 3  (0.6%) | 3  (0.6%) | 0  (0.0%) |  |
| Martins, J. N. R., et al.[73] | 2017 | Caucasian | 591 | 233  (39.4%) | 174  (29.4%) | 13  (2.2%) | 100  (16.9%) | 28  (4.7%) | 36  (6.1%) | 0  (0.0%) | 0  (0.0%) | 7  (1.2%) |  |
| Shi, Z.-Y., et al.[74] | 2017 | Chinese | 517 | 157  (30.4%) | 207  (40.0%) | 3  (0.6%) | 69  (13.3%) | 16  (3.1%) | 47  (9.1%) | 3  (0.6%) | 1  (0.2%) | 14  (2.7%) |  |
| Abella, F., et al.[76] | 2015 | Spanish | 374 | 147  (39.3%) | 84  (22.5%) | 27  (7.2%) | 74  (19.8%) | 16  (4.3%) | 12  (3.2%) | 8  (2.1%) | 6  (1.6%) | 0  (0.0%) |  |
| Bulut, D. G., et al.[77] | 2015 | Turkish | 565 | 439  (77.7%) | 71  (12.6%) | 6  (1.1%) | 37  (6.5%) | 11  (1.9%) | 1  (0.2%) | 0  (0.0%) | 0  (0.0%) | 0  (0.0%) |  |
| Felsypremila, G., et al.[35] | 2015 | Indian | 356 | 196  (55.1%) | 90  (25.2%) | 7  (1.9%) | 53  (15.0%) | 7  (1.9%) | 3  (0.9%) | 0  (0.0%) | 0  (0.0%) | 0  (0.0%) |  |
| Ok, E., et al.[79] | 2014 | Turkish | 1301 | 709  (54.5%) | 115  (8.8%) | 47  (3.6%) | 285  (21.9%) | 141  (10.8%) | 0  (0.0%) | 0  (0.0%) | 4  (0.3%) | 0  (0.0%) |  |
| Yang, L., et al.[85] | 2014 | Chinese | 392 | 178  (45.4%) | 64  (16.3%) | 45  (11.5%) | 79  (20.2%) | 25  (6.4%) | 0  (0.0%) | 0  (0.0%) | 1  (0.3%) | 0  (0.0%) |  |
| Total |  |  | 15806 | 7738  (49.0%) | 2564  (16.2%) | 1144  (7.2%) | 2676  (16.9%) | 1045  (6.6%) | 340  (2.2%) | 155  (1.0%) | 65  (0.4%) | 79  (0.5%) |  |
| Mandibular PM1s | | | | | | | | | | | | | |
| Erkan, E., et al.[44] | 2023 | Turkish | 814 | 692  (85.0%) | 17  (2.1%) | 1  (0.1%) | 46  (5.7%) | 52  (6.4%) | 2  (0.25) | 0  (0.0%) | 4  (0.5%) | 0  (0.0%) |  |
| Mirah, M.A., et al.[46] | 2023 | Saudi | 663 | 466  (70.3%) | 2  (0.3%) | 15  (2.3%) | 13  (2.0%) | 165  (24.9%) | 0  (0.0%) | 0  (0.0%) | 2  (0.3%) | 0  (0.0%) |  |
| Khanna, S et al.[48] | 2023 | Gujarati | 134 | 102  (76.1%) | 4  (3.0%) | 10  (7.5%) | 4  (3.0%) | 4  (3.0%) | 10  (7.5%) | 0  (0.0%) | 0  (0.0%) | 0  (0.0%) |  |
| Rae O et al.[86] | 2023 | Melbourne | 1576 | 1258  (79.8%) | 4  (0.3%) | 74  (4.7%) | 0  (0.0%) | 182  (11.5%) | 1  (0.1%) | 0  (0.0%) | 0  (0.0%) | 0  (0.0%) | 51  (3.2%) |
| Iqbal, A., et al.[51] | 2022 | Saudi Arabian | 412 | 392  (95.1%) | 3  (0.7%) | 5  (1.2%) | 3  (0.7%) | 9  (2.2%) | 0  (0.0%) | 0  (0.0%) | 0  (0.0%) | 0  (0.0%) |  |
| Gündüz, H. et al.[23] | 2022 | Turkish | 988 | 760  (76.9%) | 1  (0.1%) | 14  (1.4%) | 100  (10.1%) | 108  (10.9%) | 0  (0.0%) | 1  (0.1%) | 4  (0.4%) | 0  (0.0%) |  |
| Mashyakhy, M., et al.[87] | 2022 | Saudi | 397 | 276  (69.5%) | 0  (0.0%) | 25  (6.3%) | 0  (0.0%) | 88  (22.2%) | 0  (0.0%) | 1  (0.3%) | 0  (0.0%) | 7  (1.8%) |  |
| Buchanan, G.D., et al.[24] | 2022 | Black South African | 386 | 187  (48.4%) | 8  (2.1%) | 36  (9.3%) | 12  (3.1%) | 108  (28.0%) | 1  (0.3%) | 2  (0.5%) | 1  (0.3%) | 31  (8.0%) | 0  (0.0%) |
| Choi et al.[108] | 2022 | Korean | 1463 | 1254  (85.7%) | 4  (0.3%) | 65  (4.4%) | 0  (0.0%) | 140  (9.6%) | 0  (0.0%) | 0  (0.0%) | 0  (0.0%) | 0  (0.0%) | 0  (0.0%) |
| Lemos, M.C., et al.[106] | 2022 | Brazil | 407 | 328  (80.6%) | 1  (0.2%) | 10  (2.5%) | 0  (0.0%) | 66  (16.2%) | 0  (0.0%) | 0  (0.0%) | 2  (0.5%) | 0  (0.0%) | 0  (0.0%) |
| Thanaruengrong et al.[88] | 2021 | Thai | 621 | 392  (63.1%) | 9  (1.4%) | 16  (2.6%) | 4  (0.6%) | 177  (28.5%) | 2  (0.3%) | 1  (0.2%) | 0  (0.0%) | 20  (3.2%) | 0  (0.0%) |
| Algarni, Y. A., et al.[89] | 2021 | Saudi | 216 | 148  (68.5%) | 24  (11.1%) | 13  (6.0%) | 3  (1.4%) | 26  (12.0%) | 2  (0.9%) | 0  (0.0%) | 0  (0.0%) | 0  (0.0%) | 0  (0.0%) |
| Hasheminia, S.M., et al.[25] | 2021 | Iranian | 389 | 317  (81.5%) | 24  (6.2%) | 4  (1.0%) | 2  (0.5%) | 34  (8.7%) | 3  (0.8%) | 2  (0.5%) | 0  (0.0%) | 3  (0.8%) |  |
| Arayasantiparb, R. et al.[90] | 2021 | Thai | 349 | 281  (80.5%) | 2  (0.6%) | 1  (0.3%) | 0  (0.0%) | 59  (16.9%) | 0  (0.0%) | 1  (0.3%) | 0  (0.0%) | 5  (1.4%) |  |
| Alfonso-Rodriguez, C.A., et al.[91] | 2021 | Colombian | 100 | 53  (53.0%) | 0  (0.0%) | 4  (4.0%) | 7  (7.0%) | 29  (29.0%) | 5  (5.0%) | 1  (1.0%) | 1  (1.0%) | 0  (0.0%) |  |
| Mishra, S., et al.[92] | 2021 | Delhi-NCR | 216 | 86  (39.8%) | 58  (26.9%) | 23  (10.6%) | 12  (5.6%) | 26  (12.0%) | 9  (4.2%) | 0  (0.0%) | 1  (0.5%) | 1  (0.5%) |  |
| Alenezi, D.J., et al.[94] | 2020 | Kuwaiti | 245 | 37  (15.1%) | 43  (17.6%) | 41  (16.7%) | 20  (8.2%) | 19  (7.8%) | 34  (13.9%) | 5  (2.0%) | 0  (0.0%) | 46  (18.8%) |  |
| Shemesh, A., et al.[109] | 2020 | Israeli | 1835 | 1432  (78.0%) | 12  (0.7%) | 214  (11.7%) | 28  (1.5%) | 106  (5.8%) | 0  (0.0%) | 8  (0.4%) | 11  (0.6%) | 24  (1.3%) | 0  (0.0%) |
| Wu, D., et al.[64] | 2020 | Chinese | 1296 | 1051  (81.1%) | 0  (0.0%) | 39  (3.0%) | 0  (0.0%) | 160  (12.3%) | 0  (0.0%) | 0  (0.0%) | 6  (0.5%) | 0  (0.0%) | 40  (3.1%) |
| Alfawaz, H., et al.[26] | 2019 | Saudi | 391 | 344  (88.0%) | 14  (3.6%) | 12  (3.1%) | 8  (2.0%) | 6  (1.5%) | 1  (0.3%) | 0  (0.0%) | 6  (1.5%) | 0  (0.0%) | 0  (0.0%) |
| Buyukbayram, I. K., et al.[110] | 2019 | Turkish | 327 | 274  (83.8%) | 0  (0.0%) | 10  (3.1%) | 0  (0.0%) | 40  (12.2%) | 0  (0.0%) | 0  (0.0%) | 0  (0.0%) | 3  (0.9%) | 0  (0.0%) |
| Corbella, S., et al.[95] | 2019 | Caucasians | 96 | 76  (79.2%) | 7  (7.3%) | 0  (0.0%) | 11  (11.5%) | 2  (2.1%) | 0  (0.0%) | 0  (0.0%) | 0  (0.0%) | 0  (0.0%) | 0  (0.0%) |
| Jang, Y. E., et al.[96] | 2019 | Korean | 1007 | 765  (76.0%) | 22  (2.2%) | 36  (3.6%) | 4  (0.4%) | 134  (13.3%) | 0  (0.0%) | 1  (0.1%) | 0  (0.0%) | 9  (0.9%) | 36  (3.6%) |
| Pan, J. Y. Y., et al.[67] | 2019 | Malaysian | 365 | 301  (82.5%) | 1  (0.3%) | 5  (1.4%) | 3  (0.8%) | 55  (15.1%) | 0  (0.0%) | 0  (0.0%) | 0  (0.0%) | 0  (0.0%) | 0  (0.0%) |
| Martins, J. N. R., et al.[70] | 2018 | Asian | 238 | 206  (86.6%) | 0  (0.0%) | 0  (0.0%) | 1  (0.4%) | 30  (12.6%) | 0  (0.0%) | 0  (0.0%) | 0  (0.0%) | 1  (0.4%) | 0  (0.0%) |
| Martins, J. N. R., et al.[70] | 2018 | White | 1089 | 846  (77.7%) | 27  (2.5%) | 58  (5.3%) | 16  (1.5%) | 133  (12.2%) | 0  (0.0%) | 2  (0.2%) | 0  (0.0%) | 7  (0.6%) | 0  (0.0%) |
| Pedemonte, E., et al.[97] | 2018 | Belgium | 106 | 84  (79.2%) | 0  (0.0%) | 0  (0.0%) | 0  (0.0%) | 14  (13.2%) | 0  (0.0%) | 0  (0.0%) | 0  (0.0%) | 8  (7.5%) | 0  (0.0%) |
| Pedemonte, E., et al.[97] | 2018 | Chilean | 100 | 69  (69.0%) | 0  (0.0%) | 7  (7.0%) | 0  (0.0%) | 12  (12.0%) | 0  (0.0%) | 0  (0.0%) | 0  (0.0%) | 12  (12.0%) | 0  (0.0%) |
| Vega-Lizama et al.[98] | 2018 | Yucatecan | 146 | 58  (39.7%) | 0  (0.0%) | 24  (16.4%) | 0  (0.0%) | 31  (21.2%) | 0  (0.0%) | 4  (2.7%) | 0  (0.0%) | 29  (19.9%) |  |
| Burklein, S., et al.[27] | 2017 | German | 1054 | 229  (21.7%) | 55  (5.2%) | 2  (0.2%) | 153  (14.5%) | 582  (55.2%) | 27  (2.6%) | 4  (0.4%) | 2  (0.2%) | 0  (0.0%) | 0  (0.0%) |
| Hajihassani, N., et al.[99] | 2017 | Iranian | 119 | 74  (62.2%) | 1  (0.8%) | 13  (10.9%) | 1  (0.8%) | 24  (20.2%) | 5  (4.2%) | 1  (0.8%) | 0  (0.0%) | 0  (0.0%) | 0  (0.0%) |
| Martins, J. N. R., et al.[73] | 2017 | Caucasian | 1054 | 817  (77.5%) | 26  (2.5%) | 58  (5.5%) | 16  (1.5%) | 128  (12.1%) | 0  (0.0%) | 2  (0.2%) | 0  (0.0%) | 7  (0.7%) | 0  (0.0%) |
| Arslan, H., et al.[111] | 2015 | Turkish | 154 | 110  (71.4%) | 2  (1.3%) | 4  (2.6%) | 0  (0.0%) | 31  (20.1%) | 0  (0.0%) | 0  (0.0%) | 0  (0.0%) | 3  (1.9%) | 4  (2.6%) |
| Bulut, D. G., et al.[77] | 2015 | Turkish | 621 | 585  (94.2%) | 4  (0.6%) | 7  (1.1%) | 5  (0.8%) | 20  (3.2%) | 0  (0.0%) | 0  (0.0%) | 0  (0.0%) | 0  (0.0%) | 0  (0.0%) |
| Felsypremila, G., et al.[35] | 2015 | Indian | 438 | 411  (94.0%) | 6  (1.3%) | 0  (0.0%) | 12  (2.7%) | 6  (1.3%) | 0  (0.0%) | 0  (0.0%) | 0  (0.0%) | 0  (0.0%) | 3  (0.7%) |
| Llena, C., et al.[103] | 2014 | Spanish | 73 | 57  (78.1%) | 6  (8.2%) | 0  (0.0%) | 0  (0.0%) | 8  (11.0%) | 1  (1.4%) | 0  (0.0%) | 0  (0.0%) | 1  (1.4%) | 0  (0.0%) |
| Ok, E., et al.[79] | 2014 | Turkish | 1471 | 1366  (92.9%) | 4  (0.3%) | 14  (1.0%) | 21  (1.4%) | 65  (4.4%) | 0  (0.0%) | 0  (0.0%) | 1  (0.1%) | 0  (0.0%) | 0  (0.0%) |
| Shetty, A., et al.[112] | 2014 | Indian | 1186 | 994  (83.8%) | 4  (0.3%) | 25  (2.1%) | 3  (0.3%) | 142  (12.0%) | 2  (0.2%) | 0  (0.0%) | 4  (0.3%) | 1  (0.1%) | 11  (0.9%) |
| Salarpour, M., et al.[113] | 2013 | Iranian | 42 | 30  (71.4%) | 0  (0.0%) | 0  (0.0%) | 0  (0.0%) | 12  (28.6%) | 0  (0.0%) | 0  (0.0%) | 0  (0.0%) | 0  (0.0%) | 0  (0.0%) |
| Yang, H., et al.[104] | 2013 | Chinese | 440 | 335  (76.1%) | 15  (3.4%) | 12  (2.7%) | 29  (6.6%) | 41  (9.3%) | 0  (0.0%) | 0  (0.0%) | 3  (0.7%) | 0  (0.0%) | 5  (1.1%) |
| Yu, X., et al.[105] | 2012 | Chinese | 174 | 151  (86.8%) | 0  (0.0%) | 3  (1.7%) | 0  (0.0%) | 17  (9.8%) | 0  (0.0%) | 0  (0.0%) | 1  (0.6%) | 0  (0.0%) | 2  (1.1%) |
| Total |  |  | 23198 | 17694  (76.3%) | 410  (1.8%) | 900  (3.9%) | 537  (2.3%) | 3091  (13.3%) | 105  (0.5%) | 36  (0.2%) | 49  (0.2%) | 218  (0.9%) | 152  (0.7%) |
| Mandibular PM2s | | | | | | | | | | | | | |
| Erkan, E., et al.[44] | 2023 | Turkish | 701 | 669  (95.4%) | 8  (1.1%) | 3  (0.4%) | 5  (0.7%) | 12  (1.7%) | 1  (0.1%) | 0  (0.0%) | 3  (0.4%) | 1  (0.1%) |  |
| Mirah, M.A., et al.[46] | 2023 | Saudi | 600 | 539  (89.8%) | 6  (1.0%) | 5  (0.8%) | 4  (0.7%) | 46  (7.7%) | 0  (0.0%) | 0  (0.0%) | 0  (0.0%) | 0  (0.0%) |  |
| Khanna, S et al.[48] | 2023 | Gujarati | 134 | 124  (92.5%) | 4  (3.0%) | 3  (2.2%) | 0  (0.0%) | 0  (0.0%) | 3  (2.2%) | 0  (0.0%) | 0  (0.0%) | 0  (0.0%) |  |
| Rae O et al.[86] | 2023 | Melbourne | 1424 | 1362  (95.6%) | 1  (0.1%) | 11  (0.8%) | 0  (0.0%) | 35  (2.5%) | 0  (0.0%) | 0  (0.0%) | 0  (0.0%) | 0  (0.0%) | 14  (1.0%) |
| Iqbal, A., et al.[51] | 2022 | Saudi Arabian | 387 | 383  (99.0%) | 1  (0.3%) | 1  (0.3%) | 0  (0.0%) | 2  (0.5%) | 0  (0.0%) | 0  (0.0%) | 0  (0.0%) | 0  (0.0%) |  |
| Gündüz, H. et al.[23] | 2022 | Turkish | 974 | 934  (95.9%) | 0  (0.0%) | 4  (0.4%) | 11  (1.1%) | 20  (2.1%) | 0  (0.0%) | 0  (0.0%) | 3  (0.3%) | 2  (0.2%) |  |
| Mashyakhy, M., et al.[87] | 2022 | Saudi | 379 | 367  (96.8%) | 0  (0.0%) | 6  (1.6%) | 0  (0.0%) | 3  (0.8%) | 0  (0.0%) | 0  (0.0%) | 0  (0.0%) | 3  (0.8%) |  |
| Buchanan, G.D., et al.[24] | 2022 | Black South African | 386 | 314  (81.3%) | 4  (1.0%) | 23  (6.0%) | 2  (0.5%) | 12  (3.1%) | 1  (0.3%) | 2  (0.5%) | 4  (1.0%) | 24  (6.2%) | 0  (0.0%) |
| Choi et al.[108] | 2022 | Korean | 1448 | 1441  (99.5%) | 0  (0.0%) | 4  (0.3%) | 0  (0.0%) | 3  (0.2%) | 0  (0.0%) | 0  (0.0%) | 0  (0.0%) | 0  (0.0%) | 0  (0.0%) |
| Alghamdi, et al.[40] | 2022 | Saudi | 2400 | 2350  (97.9%) | 28  (1.2%) | 2  (0.1%) | 14  (0.6%) | 4  (0.2%) | 2  (0.1%) | 0  (0.0%) | 0  (0.0%) | 0  (0.0%) | 0  (0.0%) |
| Lemos, M.C., et al.[106] | 2022 | Brazil | 315 | 302  (95.9%) | 2  (0.6%) | 1  (0.3%) | 0  (0.0%) | 9  (2.9%) | 0  (0.0%) | 0  (0.0%) | 1  (0.3%) | 0  (0.0%) | 0  (0.0%) |
| Thanaruengrong et al.[88] | 2021 | Thai | 538 | 527  (98.0%) | 1  (0.2%) | 2  (0.4%) | 0  (0.0%) | 8  (1.5%) | 0  (0.0%) | 0  (0.0%) | 0  (0.0%) | 0  (0.0%) | 0  (0.0%) |
| Hasheminia, S.M., et al.[25] | 2021 | Iranian | 384 | 321  (83.6%) | 17  (4.4%) | 3  (0.8%) | 1  (0.3%) | 30  (7.8%) | 4  (1.0%) | 0  (0.0%) | 0  (0.0%) | 8  (2.1%) |  |
| Arayasantiparb, R. et al.[90] | 2021 | Thai | 416 | 400  (96.2%) | 0  (0.0%) | 4  (1.0%) | 0  (0.0%) | 12  (2.9%) | 0  (0.0%) | 0  (0.0%) | 0  (0.0%) | 0  (0.0%) |  |
| Mishra, S., et al.[92] | 2021 | Delhi-NCR | 216 | 130  (60.2%) | 67  (31.0%) | 6  (2.8%) | 1  (0.5%) | 7  (3.2%) | 4  (1.9%) | 1  (0.5%) | 0  (0.0%) | 0  (0.0%) |  |
| Alenezi, D.J., et al.[94] | 2020 | Kuwaiti | 231 | 30  (13.0%) | 46  (19.9%) | 26  (11.3%) | 20  (8.7%) | 9  (3.9%) | 34  (14.7%) | 4  (1.7%) | 0  (0.0%) | 56  (24.2%) | 6  (2.6%) |
| Shemesh, A., et al.[109] | 2020 | Israeli | 1678 | 1628  (97.0%) | 6  (0.4%) | 23  (1.4%) | 1  (0.1%) | 10  (0.6%) | 0  (0.0%) | 0  (0.0%) | 4  (0.2%) | 6  (0.4%) | 0  (0.0%) |
| Alfawaz, H., et al.[26] | 2019 | Saudi | 343 | 309  (90.1%) | 15  (4.4%) | 1  (0.3%) | 9  (2.6%) | 3  (0.9%) | 0  (0.0%) | 0  (0.0%) | 6  (1.7%) | 0  (0.0%) | 0  (0.0%) |
| Buyukbayram, I. K., et al.[110] | 2019 | Turkish | 264 | 258  (97.7%) | 0  (0.0%) | 3  (1.1%) | 0  (0.0%) | 1  (0.4%) | 0  (0.0%) | 0  (0.0%) | 0  (0.0%) | 2  (0.8%) | 0  (0.0%) |
| Corbella, S., et al.[95] | 2019 | Caucasians | 88 | 84  (95.5%) | 0  (0.0%) | 0  (0.0%) | 4  (4.5%) | 0  (0.0%) | 0  (0.0%) | 0  (0.0%) | 0  (0.0%) | 0  (0.0%) | 0  (0.0%) |
| Jang, Y. E., et al.[96] | 2019 | Korean | 997 | 981  (98.4%) | 14  (1.4%) | 2  (0.2%) | 0  (0.0%) | 0  (0.0%) | 0  (0.0%) | 0  (0.0%) | 0  (0.0%) | 0  (0.0%) | 0  (0.0%) |
| Pan, J. Y. Y., et al.[67] | 2019 | Malaysian | 399 | 397  (99.5%) | 1  (0.3%) | 0  (0.0%) | 1  (0.3%) | 0  (0.0%) | 0  (0.0%) | 0  (0.0%) | 0  (0.0%) | 0  (0.0%) | 0  (0.0%) |
| Martins, J. N. R., et al.[70] | 2018 | Asian | 236 | 235  (99.6%) | 1  (0.4%) | 0  (0.0%) | 0  (0.0%) | 0  (0.0%) | 0  (0.0%) | 0  (0.0%) | 0  (0.0%) | 0  (0.0%) | 0  (0.0%) |
| Martins, J. N. R., et al.[70] | 2018 | White | 858 | 821  (95.7%) | 7  (0.8%) | 11  (1.3%) | 4  (0.5%) | 12  (1.4%) | 0  (0.0%) | 0  (0.0%) | 0  (0.0%) | 3  (0.3%) | 0  (0.0%) |
| Pedemonte, E., et al.[97] | 2018 | Belgium | 101 | 93  (92.1%) | 0  (0.0%) | 3  (3.0%) | 0  (0.0%) | 5  (5.0%) | 0  (0.0%) | 0  (0.0%) | 0  (0.0%) | 0  (0.0%) | 0  (0.0%) |
| Pedemonte, E., et al.[97] | 2018 | Chilean | 100 | 95  (95.0%) | 0  (0.0%) | 2  (2.0%) | 0  (0.0%) | 2  (2.0%) | 0  (0.0%) | 0  (0.0%) | 0  (0.0%) | 1  (1.0%) | 0  (0.0%) |
| Burklein, S., et al.[27] | 2017 | German | 870 | 340  (39.1%) | 10  (1.1%) | 1  (0.1%) | 12  (1.4%) | 497  (57.1%) | 4  (0.5%) | 3  (0.3%) | 3  (0.3%) | 0  (0.0%) | 0  (0.0%) |
| Hajihassani, N., et al.[99] | 2017 | Iranian | 100 | 78  (78.0%) | 3  (3.0%) | 11  (11.0%) | 0  (0.0%) | 7  (7.0%) | 1  (1.0%) | 0  (0.0%) | 0  (0.0%) | 0  (0.0%) | 0  (0.0%) |
| Martins, J. N. R., et al.[73] | 2017 | Caucasian | 833 | 797  (95.7%) | 7  (0.8%) | 11  (1.3%) | 4  (0.5%) | 12  (1.4%) | 0  (0.0%) | 0  (0.0%) | 0  (0.0%) | 2  (0.2%) | 0  (0.0%) |
| Arslan, H., et al.[111] | 2015 | Turkish | 133 | 123  (92.5%) | 3  (2.3%) | 1  (0.8%) | 0  (0.0%) | 2  (1.5%) | 0  (0.0%) | 0  (0.0%) | 0  (0.0%) | 2  (1.5%) | 2  (1.5%) |
| Bulut, D. G., et al.[77] | 2015 | Turkish | 555 | 549  (98.9%) | 1  (0.2%) | 2  (0.4%) | 0  (0.0%) | 3  (0.5%) | 0  (0.0%) | 0  (0.0%) | 0  (0.0%) | 0  (0.0%) | 0  (0.0%) |
| Felsypremila, G., et al.[35] | 2015 | Indian | 398 | 392  (98.4%) | 0  (0.0%) | 0  (0.0%) | 0  (0.0%) | 3  (0.8%) | 0  (0.0%) | 0  (0.0%) | 0  (0.0%) | 0  (0.0%) | 3  (0.8%) |
| Llena, C., et al.[103] | 2014 | Spanish | 53 | 48  (90.6%) | 1  (1.9%) | 0  (0.0%) | 0  (0.0%) | 4  (7.5%) | 0  (0.0%) | 0  (0.0%) | 0  (0.0%) | 0  (0.0%) | 0  (0.0%) |
| Ok, E., et al.[79] | 2014 | Turkish | 1345 | 1325  (98.5%) | 1  (0.1%) | 1  (0.1%) | 8  (0.6%) | 7  (0.5%) | 0  (0.0%) | 0  (0.0%) | 3  (0.2%) | 0  (0.0%) | 0  (0.0%) |
| Shetty, A., et al.[112] | 2014 | Indian | 814 | 761  (93.5%) | 12  (1.5%) | 2  (0.2%) | 0  (0.0%) | 32  (3.9%) | 0  (0.0%) | 0  (0.0%) | 1  (0.1%) | 0  (0.0%) | 6  (0.7%) |
| Salarpour, M., et al.[113] | 2013 | Iranian | 41 | 31  (75.6%) | 0  (0.0%) | 0  (0.0%) | 0  (0.0%) | 9  (22.0%) | 0  (0.0%) | 0  (0.0%) | 0  (0.0%) | 1  (2.4%) | 0  (0.0%) |
| Yu, X., et al.[105] | 2012 | Chinese | 178 | 173  (97.2%) | 1  (0.6%) | 0  (0.0%) | 0  (0.0%) | 3  (1.7%) | 0  (0.0%) | 0  (0.0%) | 0  (0.0%) | 0  (0.0%) | 1  (0.6%) |
| Total |  |  | 21317 | 19711  (92.5%) | 268  (1.3%) | 178  (0.8%) | 101  (0.5%) | 824  (3.9%) | 54  (0.3%) | 10  (<0.1%) | 28  (0.1%) | 111  (0.5%) | 32  (0.2%) |

Supplementary Table 5. The number of roots, canals and Caucasians.

| Studies | Year | Population | N1 | 1 Root | 2 Roots | 3 Roots | N2 | 1 Canal | 2 Canals | 3 Canals |
| --- | --- | --- | --- | --- | --- | --- | --- | --- | --- | --- |
| Maxillary PM1s | | | | | | | | | | |
| Erkan, E., et al.[44] | 2023 | Turkish | 539 | 275  (51.0%) | 264  (49.0%) | 0  (0.0%) | 539 | 111  (20.6%) | 428  (79.4%) | 0  (0.0%) |
| Aljawhar, A M et al.[45] | 2023 | Iraqi | 572 | 273  (47.7%) | 292  (51.1%) | 7  (1.2%) |  |  |  |  |
| Mirah, M.A., et al.[46] | 2023 | Saudi | 613 | 102  (16.6%) | 505  (82.4%) | 6  (1.0%) |  |  |  |  |
| Al, Y.R., et al.[47] | 2023 | Saudi | 65 | 24  (36.9%) | 41  (63.1) | 0  (0.0%) |  |  |  |  |
| Khanna, S et al.[48] | 2023 | Gujarati | 137 | 45  (32.8%) | 92  (67.2%) | 0  (0.0%) | 137 | 34  (24.8%) | 103  (75.2%) | 0  (0.0%) |
| Shah, S.A.[49] | 2023 | Pakistani | 266 | 247  (92.9%) | 19  (7.1%) | 0  (0.0%) | 266 | 46  (17.3%) | 219  (82.3%) | 1  (0.4%) |
| Iqbal, A., et al.[51] | 2022 | Saudi Arabian | 346 | 92  (26.6%) | 252  (72.8%) | 2  (0.6%) |  |  |  |  |
| Gündüz, H. et al.[23] | 2022 | Turkish | 966 | 301  (31.2%) | 623  (64.5%) | 42  (4.3%) | 966 | 77  (8.0%) | 847  (87.7%) | 42  (4.3%) |
| Diab, H., et al.[52] | 2022 | Qatari | 442 | 119  (26.9%) | 312  (70.6%) | 11  (2.5%) | 442 | 60  (13.6%) | 371  (83.9%) | 11  (2.5%) |
| Alnaqbi, H.S.Y., et al.[54] | 2022 | UAE | 54 | 7  (13.0%) | 45  (83.3%) | 2  (3.7%) | 54 | 0  (0.0%) | 52  (96.3%) | 2  (3.7%) |
| Olczak et al.[55] | 2022 | Poland | 350 | 99  (28.3%) | 242  (69.1%) | 9  (2.6%) |  |  |  |  |
| Fournier et al.[56] | 2022 | French | 173 | 93  (53.8%) | 71  (41.0%) | 9  (5.2%) |  |  |  |  |
| Al-Zubaidi et al.[39] | 2021 | Saudi | 500 | 199  (39.8%) | 293  (58.6%) | 8  (1.6%) |  |  |  |  |
| Mashyakhy[57] | 2021 | Saudi | 351 | 143  (40.7%) | 202  (57.5%) | 6  (1.7%) | 350 | 13  (3.7%) | 327  (93.4%) | 9  (2.6%) |
| Haider, I., et al.[58] | 2021 | Pakistani | 150 | 60  (40.0%) | 90  (60.0%) | 0  (0.0%) | 150 | 2  (1.3%) | 141  (94.0%) | 6  (4.0%) |
| Nikkerdar, N., et al.[61] | 2020 | Iranian | 125 | 94  (75.2%) | 31  (24.8%) | 0  (0.0%) |  |  |  |  |
| Asheghi, B., et al.[62] | 2020 | Iranian | 462 | 232  (50.2%) | 222  (48.1%) | 8  (1.7%) |  |  |  |  |
| Kfir, A., et al.[63] | 2020 | Israeli | 400 | 143  (35.8%) | 245  (61.3%) | 12  (3.0%) | 400 | 8  (2.0%) | 380  (95.0%) | 12  (3.0%) |
| de Lima, C. O., et al.[65] | 2019 | Brazil | 496 | 90  (18.1%) | 398  (80.2%) | 8  (1.6%) |  |  |  |  |
| Maghfuri, S., et al.[66] | 2019 | Saudi | 100 | 36  (36.0%) | 61  (61.0%) | 3  (3.0%) |  |  |  |  |
| Saber, S., et al.[68] | 2019 | Egyptian | 358 | 164  (45.8%) | 190  (53.1%) | 4  (1.1%) |  |  |  |  |
| Alqedairi, A., et al.[69] | 2018 | Saudi | 334 | 79  (23.7%) | 251  (75.1%) | 4  (1.2%) |  |  |  |  |
| Martins, J. N. R., et al.[114] | 2018 | Portuguese | 714 | 348  (48.7%) | 351  (49.2%) | 15  (2.1%) |  |  |  |  |
| Nazeer, M. R., et al.[71] | 2018 | Pakistani | 114 | 36  (31.6%) | 78  (68.4%) | 0  (0.0%) | 114 | 6  (5.3%) | 102  (89.5%) | 6  (5.3%) |
| Razumova, S., et al.[72] | 2018 | Moscow | 460 | 40  (8.7%) | 420  (91.3%) | 0  (0.0%) | 460 | 28  (6.1%) | 432  (93.9%) | 0  (0.0%) |
| Burklein, S., et al.[27] | 2017 | German | 644 | 234  (36.3%) | 402  (62.4%) | 8  (1.2%) | 644 | 62  (9.6%) | 569  (88.4%) | 13  (2.0%) |
| Martins, J. N. R., et al.[73] | 2017 | Caucasian | 690 | 336  (48.7%) | 339  (49.1%) | 15  (2.2%) |  |  |  |  |
| Celikten, B., et al.[75] | 2016 | Turkish Cypriot | 437 | 236  (54.0%) | 196  (44.9%) | 4  (0.9%) |  |  |  |  |
| Abella, F., et al.[76] | 2015 | Spanish | 430 | 198  (46.0%) | 221  (51.4%) | 11  (2.6%) |  |  |  |  |
| Bulut, D. G., et al.[77] | 2015 | Turkish | 511 | 144  (28.2%) | 362  (70.8%) | 5  (1.0%) |  |  |  |  |
| Estrela, C., et al.[78] | 2015 | Brazil | 100 | 32  (32.0%) | 66  (66.0%) | 2  (2.0%) | 100 | 6  (6.0%) | 88  (88.0%) | 6  (6.0%) |
| Felsypremila, G., et al.[35] | 2015 | Indian | 418 | 204  (48.8%) | 214  (51.2%) | 0  (0.0%) | 204 | 32  (15.7%) | 172  (84.3%) | 0  (0.0%) |
| Ok, E., et al.[79] | 2014 | Turkish |  |  |  |  | 1379 | 173  (12.5%) | 1189  (86.2%) | 17  (1.2%) |
| Total |  |  | 12317 | 4725  (38.4%) | 7390  (60.0%) | 201  (1.6%) | 6205 | 658  (10.6%) | 5420  (87.3%) | 125  (2.0%) |
| Maxillary PM2s | | | | | | | | | | |
| Erkan, E., et al.[44] | 2023 | Turkish | 516 | 464  (89.9%) | 52  (10.1%) | 0  (0.0%) | 516 | 354  (68.6%) | 162  (31.4%) | 0  (0.0%) |
| Aljawhar, A M et al.[45] | 2023 | Iraqi | 544 | 478  (87.9%) | 62  (11.4%) | 4  (0.7%) |  |  |  |  |
| Mirah, M.A., et al.[46] | 2023 | Saudi | 566 | 378  (66.8%) | 186  (32.9%) | 2  (0.4%) |  |  |  |  |
| Al, Y.R., et al.[47] | 2023 | Saudi | 56 | 53  （94.6%） | 3  (5.4%) | 0  (0.0%) |  |  |  |  |
| Khanna, S et al.[48] | 2023 | Gujarati | 125 | 72  (57.6%) | 53  (42.4%) | 0  (0.0%) | 125 | 73  (58.4%) | 52  (41.6%) | 0  (0.0%) |
| Chourasia, H.R., et al.[81] | 2023 | Saudi Arabian | 602 | 474  (78.7%) | 125  (20.8%) | 3  (0.5%) | 602 | 243  (40.4%) | 356  (59.1%) | 3  (0.5%) |
| Shah, S.A.[49] | 2023 | Pakistani | 266 | 155  (58.3%) | 110  (41.4%) | 1  (0.4%) | 266 | 167  (62.8%) | 99  (37.2%) | 0  (0.0%) |
| Iqbal, A., et al.[51] | 2022 | Saudi Arabian | 298 | 242  (81.2%) | 56  (18.8%) | 0  (0.0%) |  |  |  |  |
| Gündüz, H. et al.[23] | 2022 | Turkish | 952 | 736  (77.3%) | 213  (22.4%) | 3  (0.3%) | 952 | 480  (50.4%) | 469  (49.3%) | 3  (0.3%) |
| Diab, H., et al.[52] | 2022 | Qatari | 408 | 344  (84.3%) | 64  (15.7%) | 0  (0.0%) | 408 | 287  (70.3%) | 121  (29.7%) | 0  (0.0%) |
| Selivany, B.J. et al.[83] | 2022 | Iraqi Kurdistan | 300 | 268  (89.3%) | 32  (10.7%) | 0  (0.0%) | 300 | 201  (67.0%) | 99  (33.0%) | 0  (0.0%) |
| Alnaqbi, H.S.Y., et al.[54] | 2022 | UAE | 56 | 35  (62.5%) | 21  (37.5%) | 0  (0.0%) | 56 | 1  (1.8%) | 55  (98.2%) | 0  (0.0%) |
| Fournier et al.[56] | 2022 | French | 139 | 130  (93.5%) | 7  (5.0%) | 2  (1.4%) |  |  |  |  |
| Mashyakhy[57] | 2021 | Saudi | 359 | 316  (88.0%) | 43  (12.0%) | 0  (0.0%) | 359 | 137  (38.2%) | 219  (61.0%) | 3  (0.8%) |
| Al-Zubaidi et al.[39] | 2021 | Saudi | 500 | 416  (83.2%) | 79  (15.8%) | 5  (1.0%) |  |  |  |  |
| Nikkerdar, N., et al.[61] | 2020 | Iranian | 125 | 119  (95.2%) | 6  (4.8%) | 0  (0.0%) |  |  |  |  |
| Asheghi, B., et al.[62] | 2020 | Iranian | 400 | 364  (91.0%) | 34  (8.5%) | 2  (0.5%) |  |  |  |  |
| de Lima, C. O., et al.[65] | 2019 | Brazil | 503 | 358  (71.2%) | 143  (28.4%) | 2  (0.4%) |  |  |  |  |
| Saber, S., et al.[68] | 2019 | Egyptian | 342 | 249  (72.8%) | 89  (26.0%) | 4  (1.2%) |  |  |  |  |
| Alqedairi, A., et al.[69] | 2018 | Saudi | 318 | 271  (85.2%) | 46  (14.5%) | 1  (0.3%) |  |  |  |  |
| Martins, J. N. R., et al.[114] | 2018 | Portuguese | 618 | 585  (94.7%) | 33  (5.3%) | 0  (0.0%) | 618 | 246  (39.8%) | 369  (59.7%) | 3  (0.5%) |
| Nazeer, M. R., et al.[71] | 2018 | Pakistani | 115 | 97  (84.3%) | 18  (15.7%) | 0  (0.0%) | 115 | 57  (49.6%) | 56  (48.7%) | 2  (1.7%) |
| Razumova, S., et al.[72] | 2018 | Moscow | 423 | 112  (26.5%) | 311  (73.5%) | 0  (0.0%) | 423 | 75  (17.7%) | 348  (82.3%) | 0  (0.0%) |
| Burklein, S., et al.[27] | 2017 | German | 512 | 423  (82.6%) | 87  (17.0%) | 2  (0.4%) | 512 | 221  (43.2%) | 288  (56.3%) | 3  (0.6%) |
| Martins, J. N. R., et al.[73] | 2017 | Caucasian | 591 | 558  (94.4%) | 33  (5.6%) | 0  (0.0%) |  |  |  |  |
| Celikten, B., et al.[75] | 2016 | Turkish Cypriot | 445 | 409  (91.9%) | 34  (7.6%) | 2  (0.4%) |  |  |  |  |
| Abella, F., et al.[76] | 2015 | Spanish | 374 | 310  (82.9%) | 58  (15.5%) | 6  (1.6%) |  |  |  |  |
| Bulut, D. G., et al.[77] | 2015 | Turkish | 476 | 391  (82.1%) | 85  (17.9%) | 0  (0.0%) |  |  |  |  |
| Estrela, C., et al.[78] | 2015 | Brazil | 100 | 83  (83.0%) | 17  (17.0%) | 0  (0.0%) | 100 | 25  (25.0%) | 73  (73.0%) | 2  (2.0%) |
| Felsypremila, G., et al.[35] | 2015 | Indian | 393 | 356  (90.6%) | 37  (9.4%) | 0  (0.0%) | 356 | 196  (55.1%) | 160  (44.9%) | 0  (0.0%) |
| Ok, E., et al.[79] | 2014 | Turkish |  |  |  |  | 1301 | 776  (59.6%) | 521  (40.0%) | 4  (0.3%) |
| Total |  |  | 11422 | 9246  (80.9%) | 2137  (18.7%) | 39  (0.3%) | 7009 | 3539  (50.5%) | 3447  (49.2%) | 23  (0.3%) |
| Mandibular PM1s | | | | | | | | | | |
| Erkan, E., et al.[44] | 2023 | Turkish | 814 | 778  (95.6%) | 36  (4.4%) | 0  (0.0%) | 814 | 708  (87.0%) | 106  (13.0%) | 0  (0.0%) |
| Mirah, M.A., et al.[46] | 2023 | Saudi | 663 | 562  (84.8%) | 99  (14.9%) | 2  (0.3%) |  |  |  |  |
| Al, Y.R., et al.[47] | 2023 | Saudi | 32 | 32  (100%) | 0  (0.0%) | 0  (0.0%) |  |  |  |  |
| Khanna, S et al.[48] | 2023 | Gujarati | 134 | 128  (95.5%) | 6  (4.5%) | 0  (0.0%) | 134 | 116  (86.6%) | 18  (13.4%) | 0  (0.0%) |
| Iqbal, A., et al.[51] | 2022 | Saudi Arabian | 412 | 398  (96.6%) | 14  (3.4%) | 0  (0.0%) |  |  |  |  |
| Gündüz, H. et al.[23] | 2022 | Turkish | 988 | 888  (89.9%) | 96  (9.7%) | 4  (0.4%) | 988 | 760  (76.9%) | 224  (22.7%) | 4  (0.4%) |
| Mashyakhy, M., et al.[87] | 2022 | Saudi | 397 | 395  (99.5%) | 2  (0.5%) | 0  (0.0%) | 397 | 276  (69.5%) | 117  (29.5%) | 4  (1.0%) |
| Hasheminia, S.M., et al.[25] | 2021 | Iranian | 389 | 345  (88.7%) | 41  (10.5%) | 3  (0.8%) |  |  |  |  |
| Mishra, S., et al.[92] | 2021 | Delhi-NCR | 216 | 205  (94.9%) | 11  (5.1%) | 0  (0.0%) | 216 | 86  (39.8%) | 129  (59.7%) | 1  (0.5%) |
| Algarni, Y. A., et al.[89] | 2021 | Saudi Arabian | 219 | 198  (90.4%) | 18  (8.2%) | 0  (0.0%) | 216 | 147  (68.1%) | 51  (23.6%) | 18  (8.3%) |
| Alam, F., et al.[93] | 2020 | Saudi | 752 | 608  (80.9%) | 144  (19.1%) | 0  (0.0%) | 752 | 523  (69.5%) | 229  (30.5%) | 0  (0.0%) |
| Alenezi, D.J., et al.[94] | 2020 | Kuwaiti | 245 | 181  (73.9%) | 61  (24.9%) | 3  (1.2%) |  |  |  |  |
| Alfawaz, H., et al.[26] | 2019 | Saudi | 391 | 377  (96.4%) | 12  (3.1%) | 2  (0.5%) |  |  |  |  |
| Corbella, S., et al.[95] | 2019 | Caucasians | 97 | 92  (94.8%) | 5  (5.2%) | 0  (0.0%) |  |  |  |  |
| Martins, J. N. R., et al.[114] | 2018 | Portuguese | 1089 | 1087  (99.8%) | 2  (0.2%) | 0  (0.0%) | 1089 | 846  (77.7%) | 237  (21.8%) | 6  (0.6%) |
| Pedemonte, E., et al.[97] | 2018 | Belgium | 101 | 101  (100.0%) | 0  (0.0%) | 0  (0.0%) | 101 | 84  (83.2%) | 17  (16.8%) | 0  (0.0%) |
| Razumova, S., et al.[72] | 2018 | Moscow | 490 | 490  (100.0%) | 0  (0.0%) | 0  (0.0%) | 490 | 437  (89.2%) | 53  (10.8%) | 0  (0.0%) |
| Burklein, S., et al.[27] | 2017 | German | 1044 | 954  (91.4%) | 90  (8.6%) | 0  (0.0%) | 1044 | 813  (77.9%) | 229  (21.9%) | 2  (0.2%) |
| Hajihassani, N., et al.[99] | 2017 | Iranian | 124 | 119  (96.0%) | 5  (4.0%) | 0  (0.0%) |  |  |  |  |
| Martins, J. N. R., et al.[73] | 2017 | Caucasian | 1054 | 1052  (99.8%) | 2  (0.2%) | 0  (0.0%) |  |  |  |  |
| Bulut, D. G., et al.[77] | 2015 | Turkish | 604 | 581  (96.2%) | 23  (3.8%) | 0  (0.0%) |  |  |  |  |
| Estrela, C., et al.[78] | 2015 | Brazil | 100 | 99  (99.0%) | 1  (1.0%) | 0  (0.0%) | 100 | 70  (70.0%) | 29  (29.0%) | 1  (1.0%) |
| Felsypremila, G., et al.[35] | 2015 | Indian | 447 | 438  (98.0%) | 9  (2.0%) | 0  (0.0%) | 438 | 414  (94.5%) | 24  (5.5%) | 0  (0.0%) |
| Kazemipoor, M., et al.[100] | 2015 | Iranian | 914 | 790  (86.4%) | 124  (13.6%) | 0  (0.0%) | 914 | 622  (68.1%) | 292  (31.9%) | 0  (0.0%) |
| Kazemipoor, M., et al.[101] | 2015 | Iranian | 460 | 394  (85.7%) | 66  (14.3%) | 0  (0.0%) | 460 | 294  (63.9%) | 166  (36.1%) | 0  (0.0%) |
| Llena, C., et al.[103] | 2014 | Spanish |  |  |  |  | 73 | 57  (78.1%) | 15  (20.5%) | 1  (1.4%) |
| Ok, E., et al.[79] | 2014 | Turkish |  |  |  |  | 1471 | 1375  (93.5%) | 95  (6.5%) | 1  (0.1%) |
| Total |  |  | 12176 | 11292  (92.7%) | 867  (7.1%) | 14  (0.1%) | 9697 | 7628  (78.7%) | 2031  (20.9%) | 38  (0.4%) |
| Mandibular PM2s | | | | | | | | | | |
| Erkan, E., et al.[44] | 2023 | Turkish | 701 | 696  (99.3%) | 5  (0.7%) | 0  (0.0%) | 701 | 679  (96.9%) | 22  (3.1%) | 0  (0.0%) |
| Mirah, M.A., et al.[46] | 2023 | Saudi | 600 | 576  (96.0%) | 24  (4.0%) | 0  (0.0%) |  |  |  |  |
| Al, Y.R., et al.[47] | 2023 | Saudi | 59 | 59  (100%) | 0  (0.0%) | 0  (0.0%) |  |  |  |  |
| Khanna, S et al.[48] | 2023 | Gujarati | 134 | 134  (100%) | 0  (0.0%) | 0  (0.0%) | 134 | 131  (97.8%) | 3  (2.2%) | 0  (0.0%) |
| Iqbal, A., et al.[51] | 2022 | Saudi Arabian | 387 | 379  (97.9%) | 8  (2.1%) | 0  (0.0%) |  |  |  |  |
| Gündüz, H. et al.[23] | 2022 | Turkish | 974 | 958  (98.4%) | 13  (1.3%) | 3  (0.3%) | 988 | 934  (94.5%) | 35  (3.5%) | 5  (0.5%) |
| Mashyakhy, M., et al.[87] | 2022 | Saudi | 379 | 379  (100.0%) | 0  (0.0%) | 0  (0.0%) | 379 | 367  (96.8%) | 8  (2.1%) | 4  (1.1%) |
| Alghamdi, et al.[40] | 2022 | Saudi | 2400 | 2360  (98.3%) | 40  (1.7%) | 0  (0.0%) |  |  |  |  |
| Hasheminia, S.M., et al.[25] | 2021 | Iranian | 384 | 341  (88.8%) | 35  (9.1%) | 8  (2.1%) |  |  |  |  |
| Mishra, S., et al.[92] | 2021 | Delhi-NCR | 216 | 212  (98.1%) | 4  (1.9%) | 0  (0.0%) | 216 | 126  (58.3%) | 90  (41.7%) | 0  (0.0%) |
| Alam, F., et al.[93] | 2020 | Saudi | 752 | 664  (88.3%) | 88  (11.7%) | 0  (0.0%) | 752 | 540  (71.8%) | 212  (28.2%) | 0  (0.0%) |
| Alenezi, D.J., et al.[94] | 2020 | Kuwaiti | 231 | 183  (79.2%) | 48  (20.8%) | 0  (0.0%) |  |  |  |  |
| Alfawaz, H., et al.[26] | 2019 | Saudi | 343 | 328  (95.6%) | 13  (3.8%) | 2  (0.6%) |  |  |  |  |
| Corbella, S., et al.[95] | 2019 | Caucasians | 88 | 85  (96.6%) | 3  (3.4%) | 0  (0.0%) |  |  |  |  |
| Martins, J. N. R., et al.[114] | 2018 | Portuguese | 858 | 857  (99.9%) | 1  (0.1%) | 0  (0.0%) |  |  |  |  |
| Pedemonte, E., et al.[97] | 2018 | Belgium | 101 | 99  (98.0%) | 2  (2.0%) | 0  (0.0%) | 173 | 92  (53.2%) | 81  (46.8%) | 0  (0.0%) |
| Razumova, S., et al.[72] | 2018 | Moscow | 443 | 442  (99.8%) | 1  (0.2%) | 0  (0.0%) | 443 | 399  (90.1%) | 44  (9.9%) | 0  (0.0%) |
| Burklein, S., et al.[27] | 2017 | German | 871 | 859  (98.6%) | 11  (1.3%) | 1  (0.1%) | 870 | 836  (96.1%) | 31  (3.6%) | 3  (0.3%) |
| Hajihassani, N., et al.[99] | 2017 | Iranian | 100 | 100  (100.0%) | 0  (0.0%) | 0  (0.0%) |  |  |  |  |
| Martins, J. N. R., et al.[73] | 2017 | Caucasian | 833 | 832  (99.9%) | 1  (0.1%) | 0  (0.0%) |  |  |  |  |
| Bulut, D. G., et al.[77] | 2015 | Turkish | 549 | 543  (98.9%) | 6  (1.1%) | 0  (0.0%) |  |  |  |  |
| Estrela, C., et al.[78] | 2015 | Brazil | 100 | 100  (100.0%) | 0  (0.0%) | 0  (0.0%) | 100 | 97  (97.0%) | 3  (3.0%) | 0  (0.0%) |
| Felsypremila, G., et al.[35] | 2015 | Indian | 398 | 398  (100.0%) | 0  (0.0%) | 0  (0.0%) | 398 | 397  (99.7%) | 1  (0.3%) | 0  (0.0%) |
| Kazemipoor, M., et al.[100] | 2015 | Iranian | 914 | 868  (95.0%) | 46  (5.0%) | 0  (0.0%) | 914 | 737  (80.6%) | 177  (19.4%) | 0  (0.0%) |
| Kazemipoor, M., et al.[101] | 2015 | Iranian | 450 | 426  (94.7%) | 24  (5.3%) | 0  (0.0%) | 460 | 360  (78.3%) | 100  (21.7%) | 0  (0.0%) |
| Llena, C., et al.[103] | 2014 | Spanish |  |  |  |  | 53 | 48  (90.6%) | 5  (9.4%) | 0  (0.0%) |
| Ok, E., et al.[79] | 2014 | Turkish |  |  |  |  | 1345 | 1325  (98.5%) | 17  (1.3%) | 3  (0.2%) |
| Total |  |  | 13265 | 12878  (97.1%) | 373  (2.8%) | 14  (0.1%) | 7926 | 7068  (89.2%) | 829  (10.5%) | 15  (0.2%) |

Supplementary Table 6. The number of roots, canals and Asians.

| Studies | Year | Population | N1 | 1 Root | 2 Roots | 3 Roots | N2 | 1 Canal | 2 Canals | 3 Canals |
| --- | --- | --- | --- | --- | --- | --- | --- | --- | --- | --- |
| Maxillary PM1s | | | | | | | | | | |
| Alnaqbi, H.S.Y., et al.[54] | 2022 | South Asian | 53 | 2  (3.8%) | 51  (96.2%) | 0  (0.0%) | 53 | 0  (0.0%) | 53  (100.0%) | 0  (0.0%) |
| Yoza T et al.[59] | 2021 | Japan | 125 | 101  (80.8%) | 22  (17.6%) | 2  (1.6%) | 125 | 42  (33.6%) | 82  (65.6%) | 1  (0.8%) |
| Liu et al.[36] | 2021 | Chinese | 880 | 618  (70.2%) | 258  (29.3%) | 4  (0.45%) |  |  |  |  |
| Wu, D., et al.[64] | 2020 | Chinese | 1268 | 855  (67.4%) | 406  (32.0%) | 7  (0.6%) |  |  |  |  |
| Li, Y.H., et al.[41] | 2018 | Chinese | 1387 | 967  (69.7%) | 413  (29.8%) | 7  (0.5%) | 1387 | 163  (11.8%) | 1214  (87.5%) | 10  (0.7%) |
| Martins, J. N. R., et al.[70] | 2018 | Asian | 238 | 198  (83.2%) | 40  (16.8%) | 0  (0.0%) | 238 | 29  (12.2%) | 209  (87.8%) | 0  (0.0%) |
| Shi, Z.-Y., et al.[74] | 2017 | Chinese | 521 | 317  (60.8%) | 197  (37.8%) | 7  (1.3%) |  |  |  |  |
| Tian, Y. Y., et al.[80] | 2012 | Chinese | 300 | 198  (66.0%) | 100  (33.3%) | 2  (0.7%) |  |  |  |  |
| Pan, J. Y. Y., et al.[67] | 2019 | Malaysian | 304 | 206  (67.8%) | 97  (31.9%) | 1  (0.3%) |  |  |  |  |
| Total |  |  | 5076 | 3462  (68.2%) | 1584  (31.2%) | 30  (0.6%) | 1803 | 234  (13.0%) | 1558  (86.4%) | 11  (0.6%) |
| Maxillary PM2s | | | | | | | | | | |
| Alnaqbi, H.S.Y., et al.[54] | 2022 | South Asian | 52 | 17  (32.7%) | 35  (67.3%) | 0  (0.0%) | 52 | 0  (0.0%) | 52  (100.0%) | 0  (0.0%) |
| Yan, Y., et al.[84] | 2021 | western Chinese | 1118 | 1053  (94.2%) | 65  (5.8%) | 0  (0.0%) | 1118 | 616  (55.1%) | 500  (44.7%) | 2  (0.2%) |
| Pan, J. Y. Y., et al.[67] | 2019 | Malaysian | 333 | 306  (91.9%) | 27  (8.1%) | 0  (0.0%) |  |  |  |  |
| Li, Y.H., et al.[41] | 2018 | Chinese | 1403 | 1350  (96.2%) | 53  (3.8%) | 0  (0.0%) | 1403 | 706  (50.3%) | 697  (49.7%) | 0  (0.0%) |
| Martins, J. N. R., et al.[70] | 2018 | Asian | 239 | 237  (99.2%) | 2  (0.8%) | 0  (0.0%) | 239 | 179  (74.9%) | 60  (25.1%) | 0  (0.0%) |
| Shi, Z.-Y., et al.[74] | 2017 | Chinese | 517 | 478  (92.5%) | 39  (7.5%) | 0  (0.0%) |  |  |  |  |
| Yang, L., et al.[85] | 2014 | Chinese | 392 | 339  (86.5%) | 53  (13.5%) | 0  (0.0%) | 392 | 178  (45.4%) | 213  (54.3%) | 1  (0.3%) |
| Total |  |  | 4054 | 3780  (93.2%) | 274  (6.8%) | 0  (0.0%) | 3204 | 1679  (52.4%) | 1522  (47.5%) | 3  (0.1%) |
| Mandibular PM1s | | | | | | | | | | |
| Thanaruengrong et al.[88] | 2021 | Thai | 621 | 609  (98.1%) | 10  (1.6%) | 2  (0.3%) |  |  |  |  |
| Arayasantiparb, R. et al.[90] | 2021 | Thai | 349 | 329  (94.3%) | 17  (4.9%) | 3  (0.9%) |  |  |  |  |
| Wu, D., et al.[64] | 2020 | Chinese | 1296 | 1280  (98.8%) | 16  (1.2%) | 0  (0.0%) |  |  |  |  |
| Pan, J. Y. Y., et al.[67] | 2019 | Malaysian | 359 | 353  (98.3%) | 6  (1.7%) | 0  (0.0%) |  |  |  |  |
| Jang, Y. E., et al.[96] | 2019 | Korean | 971 | 951  (97.9%) | 20  (2.1%) | 0  (0.0%) |  |  |  |  |
| Martins, J. N. R., et al.[70] | 2018 | Asian | 238 | 238  (100.0%) | 0  (0.0%) | 0  (0.0%) | 238 | 206  (86.6%) | 31  (13.0%) | 1  (0.4%) |
| Vega-Lizama et al.[98] | 2018 | Yucatecan | 105 | 102  (97.1%) | 3  (2.9%) | 0  (0.0%) | 102 | 54  (52.9%) | 35  (34.3%) | 10  (9.8%) |
| Huang, Y. D., et al.[102] | 2014 | Taiwanese | 300 | 246  (82.0%) | 51  (17.0%) | 3  (1.0%) | 300 | 197  (65.7%) | 100  (33.3%) | 3  (1.0%) |
| Yang, H., et al.[104] | 2013 | Chinese |  |  |  |  | 435 | 335  (77.0%) | 97  (22.3%) | 3  (0.7%) |
| Yu, X., et al.[105] | 2012 | Chinese | 178 | 174  (97.8%) | 4  (2.2%) | 0  (0.0%) | 176 | 155  (88.1%) | 20  (11.4%) | 1  (0.6%) |
| Total |  |  | 4417 | 4282  (96.9%) | 127  (2.9%) | 8  (0.2%) | 1251 | 947  (75.7%) | 283  (22.6%) | 18  (1.4%) |
| Mandibular PM2s | | | | | | | | | | |
| Thanaruengrong et al.[88] | 2021 | Thai | 538 | 537  (99.8%) | 1  (0.2%) | 0  (0.0%) |  |  |  |  |
| Arayasantiparb, R. et al.[90] | 2021 | Thai | 416 | 416  (100.0%) | 0  (0.0%) | 0  (0.0%) |  |  |  |  |
| Pan, J. Y. Y., et al.[67] | 2019 | Malaysian | 399 | 399  (100.0%) | 0  (0.0%) | 0  (0.0%) |  |  |  |  |
| Jang, Y. E., et al.[96] | 2019 | Korean | 997 | 997  (100.0%) | 0  (0.0%) | 0  (0.0%) |  |  |  |  |
| Martins, J. N. R., et al.[70] | 2018 | Asian | 236 | 236  (100.0%) | 0  (0.0%) | 0  (0.0%) | 236 | 235  (99.6%) | 1  (0.4%) | 0  (0.0%) |
| Yu, X., et al.[105] | 2012 | Chinese | 178 | 178  (100.0%) | 0  (0.0%) | 0  (0.0%) | 177 | 173  (97.7%) | 4  (2.3%) | 0  (0.0%) |
| Total |  |  | 2764 | 2763  (100.0%) | 1  (<0.1%) | 0  (0.0%) | 413 | 408  (98.8%) | 5  (1.2%) | 0  (0.0%) |

Supplementary Table 7. Root canal configuration and Caucasians.

| Studies | Year | Population | N3 | Vertucci I | Vertucci II | Vertucci III | Vertucci IV | Vertucci V | Vertucci VI | Vertucci VII | Vertucci VIII | Others |  |
| --- | --- | --- | --- | --- | --- | --- | --- | --- | --- | --- | --- | --- | --- |
| Maxillary PM1s | | | | | | | | | | | | |  |
| Erkan, E., et al.[44] | 2023 | Turkish | 539 | 55  (10.2%) | 36  (6.7%) | 21  (3.9%) | 414  (77.0%) | 2  (0.4%) | 2  (0.4%) | 0  (0.0%) | 8  (1.5%) | 0  (0.0%) |  |
| Aljawhar, A M et al.[45] | 2023 | Iraqi | 572 | 32  (5.6%) | 2  (0.3%) | 79  (13.8%) | 267  (46.7%) | 95  (16.6%) | 0  (0.0%) | 48  (8.4%) | 14  (2.4%) | 35  (6.1%) |  |
| Mirah, M.A., et al.[46] | 2023 | Saudi | 613 | 15  (2.4%) | 78  (12.7%) | 0  (0.0%) | 511  (83.4%) | 3  (0.5%) | 0  (0.0%) | 0  (0.0%) | 6  (1.0%) | 0  (0.0%) |  |
| Khanna, S et al.[48] | 2023 | Gujarati | 137 | 11  (8.0%) | 12  (8.8%) | 11  (8.0%) | 90  (65.7%) | 4  (2.9%) | 6  (4.4%) | 0  (0.0%) | 0  (0.0%) | 3  (2.2%) |  |
| Shah, S.A.[49] | 2023 | Pakistan | 266 | 121  (45.5%) | 9  (3.4%) | 29  (10.9%) | 69  (25.9%) | 32  (12.0%) | 1  (0.4%) | 4  (1.5%) | 1  (0.4%) | 0  (0.0%) |  |
| Iqbal, A., et al.[51] | 2022 | Saudi Arabian | 346 | 201  (58.1%) | 134  (38.7%) | 4  (1.2%) | 4  (1.2%) | 3  (0.9%) | 0  (0.0%) | 0  (0.0%) | 0  (0.0%) | 0  (0.0%) |  |
| Gündüz, H. et al.[23] | 2022 | Turkish | 966 | 73  (7.6%) | 92  (9.5%) | 77  (8.0%) | 655  (67.8%) | 18  (1.9%) | 4  (0.4%) | 5  (0.5%) | 42  (4.3%) | 0  (0.0%) |  |
| Alnaqbi, H.S.Y., et al.[54] | 2022 | UAE | 54 | 1  (1.9%) | 3  (5.6%) | 3  (5.6%) | 14  (25.9%) | 28  (51.9%) | 2  (3.7%) | 1  (1.9%) | 0  (0.0%) | 2  (3.7%) |  |
| Lemos, M.C., et al.[106] | 2022 | Brazil | 310 | 11  (3.5%) | 61  (19.7%) | 1  (0.3%) | 229  (73.9%) | 1  (0.3%) | 0  (0.0%) | 0  (0.0%) | 7  (2.3%) | 0  (0.0%) |  |
| Olczak et al.[55] | 2022 | Poland | 350 | 6  (1.7%) | 30  (8.6%) | 9  (2.6%) | 275  (78.6%) | 18  (5.1%) | 0  (0.0%) | 2  (0.6%) | 10  (2.9%) | 0  (0.0%) |  |
| Fournier et al.[56] | 2022 | French | 177 | 87  (49.2%) | 24  (13.6%) | 4  (2.3%) | 50  (28.2%) | 9  (5.1%) | 3  (1.7%) | 0  (0.0%) | 0  (0.0%) | 0  (0.0%) |  |
| Al-Zubaidi et al.[39] | 2021 | Saudi | 500 | 26  (5.2%) | 164  (32.8%) | 3  (0.6%) | 289  (57.8%) | 10  (2%) | 0  (0.0%) | 0  (0.0%) | 8  (1.6%) | 0  (0.0%) |  |
| Mashyakhy[57] | 2021 | Saudi | 351 | 13  (3.7%) | 24  (6.8%) | 27  (7.7%) | 224  (63.8%) | 52  (14.8%) | 1  (0.3%) | 0  (0.0%) | 0  (0.0%) | 10  (2.8%) |  |
| Haider, I., et al.[58] | 2021 | Pakistan (Lahore) | 150 | 108  (72.0%) | 6  (4.0%) | 4  (2.7%) | 8  (5.3%) | 20  (13.3%) | 3  (2.0%) | 1  (0.7%) | 0  (0.0%) | 0  (0.0%) |  |
| Kfir, A., et al.[63] | 2020 | Israeli | 400 | 7  (1.8%) | 67  (16.8%) | 2  (0.5%) | 295  (73.8%) | 2  (0.5%) | 27  (6.8%) | 0  (0.0%) | 0  (0.0%) | 0  (0.0%) |  |
| Nikkerdar, N., et al.[61] | 2020 | Iranian | 125 | 23  (18.4%) | 3  (2.4%) | 25  (20.0%) | 12  (9.6%) | 50  (40.0%) | 12  (9.6%) | 0  (0.0%) | 0  (0.0%) | 0  (0.0%) |  |
| Asheghi, B., et al.[62] | 2020 | Iranian | 462 | 41  (8.9%) | 70  (15.2%) | 4  (0.9%) | 331  (71.6%) | 6  (1.3%) | 1  (0.2%) | 0  (0.0%) | 9  (1.9%) | 0  (0.0%) |  |
| de Lima, C. O., et al.[65] | 2019 | Brazil | 496 | 32  (6.5%) | 38  (7.7%) | 3  (0.6%) | 408  (82.3%) | 4  (0.8%) | 3  (0.6%) | 0  (0.0%) | 8  (1.6%) | 0  (0.0%) |  |
| Maghfuri, S., et al.[66] | 2019 | Saudi | 100 | 0  (0.0%) | 7  (7.0%) | 0  (0.0%) | 75  (75.0%) | 13  (13.0%) | 2  (2.0%) | 0  (0.0%) | 3  (3.0%) | 0  (0.0%) |  |
| Saber, S., et al.[68] | 2019 | Egyptian | 358 | 4  (1.1%) | 56  (15.6%) | 5  (1.4%) | 262  (73.2%) | 5  (1.4%) | 14  (3.9%) | 6  (1.7%) | 5  (1.4%) | 1  (0.3%) |  |
| Alqedairi, A., et al.[69] | 2018 | Saudi | 334 | 36  (10.8%) | 28  (8.4%) | 6  (1.8%) | 236  (70.7%) | 13  (3.9%) | 7  (2.1%) | 1  (0.3%) | 7  (2.1%) | 0  (0.0%) |  |
| Martins, J. N. R., et al.[70] | 2018 | White | 714 | 24  (3.4%) | 122  (17.1%) | 2  (0.3%) | 487  (68.2%) | 7  (1.0%) | 33  (4.6%) | 0  (0.0%) | 5  (0.7%) | 34  (4.8%) |  |
| Nazeer, M. R., et al.[71] | 2018 | Pakistani | 187 | 127  (67.9%) | 24  (12.8%) | 14  (7.5%) | 0  (0.0%) | 7  (3.7%) | 6  (3.2%) | 0  (0.0%) | 1  (0.5%) | 8  (4.3%) |  |
| Burklein, S., et al.[27] | 2017 | German | 652 | 25  (3.8%) | 42  (6.4%) | 0  (0.0%) | 441  (67.6%) | 51  (7.8%) | 79  (12.1%) | 1  (0.2%) | 13  (2.0%) | 0  (0.0%) |  |
| Martins, J. N. R., et al.[73] | 2017 | Caucasian | 690 | 22  (3.2%) | 119  (17.2%) | 2  (0.3%) | 469  (68.0%) | 6  (0.9%) | 33  (4.8%) | 0  (0.0%) | 5  (0.7%) | 34  (4.9%) |  |
| Abella, F., et al.[76] | 2015 | Spanish | 430 | 108  (25.1%) | 44  (10.2%) | 19  (4.4%) | 227  (52.8%) | 8  (1.9%) | 7  (1.6%) | 6  (1.4%) | 11  (2.6%) | 0  (0.0%) |  |
| Bulut, D. G., et al.[77] | 2015 | Turkish | 1252 | 784  (62.6%) | 427  (34.1%) | 10  (0.8%) | 24  (1.9%) | 7  (0.6%) | 0  (0.0%) | 0  (0.0%) | 0  (0.0%) | 0  (0.0%) |  |
| Felsypremila, G., et al.[35] | 2015 | Indian | 204 | 33  (15.9%) | 53  (26.1%) | 0  (0.0%) | 118  (58.0%) | 0  (0.0%) | 0  (0.0%) | 0  (0.0%) | 0  (0.0%) | 0  (0.0%) |  |
| Ok, E., et al.[79] | 2014 | Turkish | 1379 | 132  (9.6%) | 89  (6.5%) | 19  (1.4%) | 1061  (76.9%) | 63  (4.6%) | 1  (0.1%) | 0  (0.0%) | 14  (1.0%) | 0  (0.0%) |  |
| Total |  |  | 13114 | 2158  (16.5%) | 1864  (14.2%) | 383  (2.9%) | 7545  (57.5%) | 537  (4.1%) | 247  (1.9%) | 75  (0.6%) | 177  (1.3%) | 127  (1.0%) |  |
| Maxillary PM2s | | | | | | | | | | | | |  |
| Olczak, et al.[82] | 2023 | Poland | 324 | 193  (59.6%) | 30  (9.3%) | 20  (6.2%) | 51  (15.7%) | 23  (7.1%) | 3  (0.9%) | 3  (0.9%) | 1  (0.3%) | 0  (0.0%) |  |
| Erkan, E., et al.[44] | 2023 | Turkish | 516 | 296  (57.4%) | 30  (5.8%) | 28  (5.4%) | 149  (28.9%) | 10  (1.9%) | 2  (0.4%) | 0  (0.0%) | 1  (0.2%) | 0  (0.0%) |  |
| Aljawhar, A M et al.[45] | 2023 | Iraqi | 544 | 199 (36.6%) | 2  (0.4%) | 119 (21.9%) | 52  (9.6%) | 77 (14.2%) | 0  (0.0%) | 63  (11.6%) | 6  (1.1%) | 26  (4.8%) |  |
| Mirah, M.A., et al.[46] | 2023 | Saudi | 566 | 218  (38.5%) | 92  (16.3%) | 9  (1.6%) | 227  (40.1%) | 17  (3.0%) | 0  (0.0%) | 1  (0.2%) | 2  (0.4%) | 0  (0.0%) |  |
| Khanna, S et al.[48] | 2023 | Gujarati | 125 | 44  (35.2%) | 14  (11.2%) | 15  (12.0%) | 40  (32.0%) | 5  (4.0%) | 7  (5.6%) | 0  (0.0%) | 0  (0.0%) | 0  (0.0%) |  |
| Shah, S.A.[49] | 2023 | Pakistan | 266 | 171  (64.3%) | 11  (4.1%) | 41  (15.4%) | 12  (4.5%) | 23  (8.6%) | 2  (0.8%) | 6  (2.3%) | 0  (0.0%) | 0  (0.0%) |  |
| Iqbal, A., et al.[51] | 2022 | Saudi Arabian | 298 | 211  (70.8%) | 47  (15.8%) | 12  (4.0%) | 18  (6.0%) | 6  (2.0%) | 4  (1.3%) | 0  (0.0%) | 0  (0.0%) | 0  (0.0%) |  |
| Gündüz, H. et al.[23] | 2022 | Turkish | 952 | 480  (50.4%) | 78  (8.2%) | 104  (10.9%) | 214  (22.5%) | 63  (6.6%) | 1  (0.1%) | 9  (0.9%) | 3  (0.3%) | 0  (0.0%) |  |
| Selivany, B.J. et al.[83] | 2022 | Iraqi Kurdistan | 300 | 207  (69.0%) | 33  (11.0%) | 6  (2.0%) | 15  (5.0%) | 37  (12.3%) | 2  (0.7%) | 0  (0.0%) | 0  (0.0%) | 0  (0.0%) |  |
| Alnaqbi, H.S.Y., et al.[54] | 2022 | UAE | 56 | 1  (1.8%) | 18  (32.1%) | 15  (26.8%) | 6  (10.7%) | 11  (19.6%) | 4  (7.1%) | 1  (1.8%) | 0  (0.0%) | 0  (0.0%) |  |
| Lemos, M.C., et al.[106] | 2022 | Brazil | 284 | 134  (47.2%) | 68  (23.9%) | 8  (2.8%) | 70  (24.6%) | 4  (1.4%) | 0  (0.0%) | 0  (0.0%) | 0  (0.0%) | 0  (0.0%) |  |
| Fournier et al.[56] | 2022 | French | 139 | 71  (51.1%) | 22  (15.8%) | 17  (12.2%) | 16  (11.5%) | 7  (5.0%) | 4  (2.9%) | 1  (0.7%) | 1  (0.7%) | 0  (0.0%) |  |
| Al-Zubaidi et al.[39] | 2021 | Saudi | 500 | 302  (60.4%) | 82  (16.4%) | 32  (6.4%) | 64  (12.8%) | 14  (2.8%) | 0  (0.0%) | 1  (0.2%) | 5  (1.0%) | 0  (0.0%) |  |
| Mashyakhy[57] | 2021 | Saudi | 359 | 137  (38.2%) | 39  (10.9%) | 55  (15.3%) | 69  (19.2%) | 44  (12.3%) | 4  (1.1%) | 8  (2.2%) | 0  (0.0%) | 3  (0.8%) |  |
| Fauzi et al.[107] | 2021 | chennai | 100 | 41  (41.0%) | 9  (9.0%) | 22  (22.0%) | 3  (3.0%) | 22  (22.0%) | 0  (0.0%) | 2  (2.0%) | 1  (1.0%) | 0  (0.0%) |  |
| Nikkerdar, N., et al.[61] | 2020 | Iranian | 125 | 57  (45.6%) | 12  (9.6%) | 35  (28.0%) | 5  (4.0%) | 16  (12.8%) | 0  (0.0%) | 0  (0.0%) | 0  (0.0%) | 0  (0.0%) |  |
| Asheghi, B., et al.[62] | 2020 | Iranian | 400 | 252  (63.0%) | 78  (19.5%) | 3  (0.8%) | 57  (14.3%) | 4  (1.0%) | 2  (0.5%) | 0  (0.0%) | 4  (1.0%) | 0  (0.0%) |  |
| de Lima, C. O., et al.[65] | 2019 | Brazil | 503 | 251  (49.9%) | 47  (9.3%) | 11  (2.2%) | 164  (32.6%) | 20  (4.0%) | 4  (0.8%) | 4  (0.8%) | 2  (0.4%) | 0  (0.0%) |  |
| Saber, S., et al.[68] | 2019 | Egyptian | 342 | 55  (16.1%) | 76  (22.2%) | 6  (1.8%) | 152  (44.4%) | 10  (2.9%) | 14  (4.1%) | 25  (7.3%) | 4  (1.2%) | 0  (0.0%) |  |
| Alqedairi, A., et al.[69] | 2018 | Saudi | 318 | 157  (49.4%) | 82  (25.8%) | 16  (5.0%) | 37  (11.6%) | 18  (5.7%) | 5  (1.6%) | 0  (0.0%) | 3  (0.9%) | 0  (0.0%) |  |
| Martins, J. N. R., et al.[114] | 2018 | Portuguese | 618 | 246  (39.8%) | 177  (28.6%) | 13  (2.1%) | 106  (17.2%) | 29  (4.7%) | 40  (6.5%) | 0  (0.0%) | 0  (0.0%) | 7  (1.1%) |  |
| Nazeer, M. R., et al.[71] | 2018 | Pakistani | 133 | 71  (53.4%) | 18  (13.5%) | 8  (6.0%) | 4  (3.0%) | 6  (4.5%) | 17  (12.8%) | 0  (0.0%) | 9  (6.8%) | 0  (0.0%) |  |
| Burklein, S., et al.[27] | 2017 | German | 512 | 73  (14.3%) | 57  (11.1%) | 3  (0.6%) | 128  (25.0%) | 147  (28.7%) | 98  (19.1%) | 3  (0.6%) | 3  (0.6%) | 0  (0.0%) |  |
| Martins, J. N. R., et al.[73] | 2017 | Caucasian | 591 | 233  (39.4%) | 174  (29.4%) | 13  (2.2%) | 100  (16.9%) | 28  (4.7%) | 36  (6.1%) | 0  (0.0%) | 0  (0.0%) | 7  (1.2%) |  |
| Abella, F., et al.[76] | 2015 | Spanish | 374 | 147  (39.3%) | 84  (22.5%) | 27  (7.2%) | 74  (19.8%) | 16  (4.3%) | 12  (3.2%) | 8  (2.1%) | 6  (1.6%) | 0  (0.0%) |  |
| Bulut, D. G., et al.[77] | 2015 | Turkish | 565 | 439  (77.7%) | 71  (12.6%) | 6  (1.1%) | 37  (6.5%) | 11  (1.9%) | 1  (0.2%) | 0  (0.0%) | 0  (0.0%) | 0  (0.0%) |  |
| Felsypremila, G., et al.[35] | 2015 | Indian | 356 | 196  (55.1%) | 90  (25.2%) | 7  (1.9%) | 53  (15.0%) | 7  (1.9%) | 3  (0.9%) | 0  (0.0%) | 0  (0.0%) | 0  (0.0%) |  |
| Ok, E., et al.[79] | 2014 | Turkish | 1301 | 709  (54.5%) | 115  (8.8%) | 47  (3.6%) | 285  (21.9%) | 141  (10.8%) | 0  (0.0%) | 0  (0.0%) | 4  (0.3%) | 0  (0.0%) |  |
| Total |  |  | 11467 | 5591  (48.8%) | 1656  (14.4%) | 698  (6.1%) | 2208  (19.3%) | 816  (7.1%) | 265  (2.3%) | 135  (1.2%) | 55  (0.5%) | 43  (0.4%) |  |
| Mandibular PM1s | | | | | | | | | | | | |  |
| Erkan, E., et al.[44] | 2023 | Turkish | 814 | 692  (85.0%) | 17  (2.1%) | 1  (0.1%) | 46  (5.7%) | 52  (6.4%) | 2  (0.25) | 0  (0.0%) | 4  (0.5%) | 0  (0.0%) |  |
| Mirah, M.A., et al.[46] | 2023 | Saudi | 663 | 466  (70.3%) | 2  (0.3%) | 15  (2.3%) | 13  (2.0%) | 165  (24.9%) | 0  (0.0%) | 0  (0.0%) | 2  (0.3%) | 0  (0.0%) |  |
| Khanna, S et al.[48] | 2023 | Gujarati | 134 | 102  (76.1%) | 4  (3.0%) | 10  (7.5%) | 4  (3.0%) | 4  (3.0%) | 10  (7.5%) | 0  (0.0%) | 0  (0.0%) | 0  (0.0%) |  |
| Iqbal, A., et al.[51] | 2022 | Saudi Arabian | 412 | 392  (95.1%) | 3  (0.7%) | 5  (1.2%) | 3  (0.7%) | 9  (2.2%) | 0  (0.0%) | 0  (0.0%) | 0  (0.0%) | 0  (0.0%) |  |
| Gündüz, H. et al.[23] | 2022 | Turkish | 988 | 760  (76.9%) | 1  (0.1%) | 14  (1.4%) | 100  (10.1%) | 108  (10.9%) | 0  (0.0%) | 1  (0.1%) | 4  (0.4%) | 0  (0.0%) |  |
| Mashyakhy, M., et al.[87] | 2022 | Saudi | 397 | 276  (69.5%) | 0  (0.0%) | 25  (6.3%) | 0  (0.0%) | 88  (22.2%) | 0  (0.0%) | 1  (0.3%) | 0  (0.0%) | 7  (1.8%) |  |
| Lemos, M.C., et al.[106] | 2022 | Brazil | 407 | 328  (80.6%) | 1  (0.2%) | 10  (2.5%) | 0  (0.0%) | 66  (16.2%) | 0  (0.0%) | 0  (0.0%) | 2  (0.5%) | 0  (0.0%) |  |
| Algarni, Y. A., et al.[89] | 2021 | Saudi Arabian | 216 | 148  (68.5%) | 24  (11.1%) | 13  (6.0%) | 3  (1.4%) | 26  (12.0%) | 2  (0.9%) | 0  (0.0%) | 0  (0.0%) | 0  (0.0%) |  |
| Hasheminia, S.M., et al.[25] | 2021 | Iranian | 389 | 317  (81.5%) | 24  (6.2%) | 4  (1.0%) | 2  (0.5%) | 34  (8.7%) | 3  (0.8%) | 2  (0.5%) | 0  (0.0%) | 3  (0.8%) |  |
| Mishra, S., et al.[92] | 2021 | Delhi-NCR | 216 | 86  (39.8%) | 58  (26.9%) | 23  (10.6%) | 12  (5.6%) | 26  (12.0%) | 9  (4.2%) | 0  (0.0%) | 1  (0.5%) | 1  (0.5%) |  |
| Alenezi, D.J., et al.[94] | 2020 | Kuwaiti | 245 | 37  (15.1%) | 43  (17.6%) | 41  (16.7%) | 20  (8.2%) | 19  (7.8%) | 34  (13.9%) | 5  (2.0%) | 0  (0.0%) | 46  (18.8%) |  |
| Shemesh, A., et al.[109] | 2020 | Israeli | 1835 | 1432  (78.0%) | 12  (0.7%) | 214  (11.7%) | 28  (1.5%) | 106  (5.8%) | 0  (0.0%) | 8  (0.4%) | 11  (0.6%) | 24  (1.3%) |  |
| Alfawaz, H., et al.[26] | 2019 | Saudi | 391 | 344  (88.0%) | 14  (3.6%) | 12  (3.1%) | 8  (2.0%) | 6  (1.5%) | 1  (0.3%) | 0  (0.0%) | 6  (1.5%) | 0  (0.0%) |  |
| Buyukbayram, I. K., et al.[110] | 2019 | Turkish | 327 | 274  (83.8%) | 0  (0.0%) | 10  (3.1%) | 0  (0.0%) | 40  (12.2%) | 0  (0.0%) | 0  (0.0%) | 0  (0.0%) | 3  (0.9%) |  |
| Corbella, S., et al.[95] | 2019 | Caucasians | 96 | 76  (79.2%) | 7  (7.3%) | 0  (0.0%) | 11  (11.5%) | 2  (2.1%) | 0  (0.0%) | 0  (0.0%) | 0  (0.0%) | 0  (0.0%) |  |
| Martins, J. N. R., et al.[70] | 2018 | White | 1089 | 846  (77.7%) | 27  (2.5%) | 58  (5.3%) | 16  (1.5%) | 133  (12.2%) | 0  (0.0%) | 2  (0.2%) | 0  (0.0%) | 7  (0.6%) |  |
| Pedemonte, E., et al.[97] | 2018 | Belgium | 106 | 84  (79.2%) | 0  (0.0%) | 0  (0.0%) | 0  (0.0%) | 14  (13.2%) | 0  (0.0%) | 0  (0.0%) | 0  (0.0%) | 8  (7.5%) |  |
| Burklein, S., et al.[27] | 2017 | German | 1054 | 229  (21.7%) | 55  (5.2%) | 2  (0.2%) | 153  (14.5%) | 582  (55.2%) | 27  (2.6%) | 4  (0.4%) | 2  (0.2%) | 0  (0.0%) |  |
| Hajihassani, N., et al.[99] | 2017 | Iranian | 119 | 74  (62.2%) | 1  (0.8%) | 13  (10.9%) | 1  (0.8%) | 24  (20.2%) | 5  (4.2%) | 1  (0.8%) | 0  (0.0%) | 0  (0.0%) |  |
| Martins, J. N. R., et al.[73] | 2017 | Caucasian | 1054 | 817  (77.5%) | 26  (2.5%) | 58  (5.5%) | 16  (1.5%) | 128  (12.1%) | 0  (0.0%) | 2  (0.2%) | 0  (0.0%) | 7  (0.7%) |  |
| Arslan, H., et al.[111] | 2015 | Turkish | 154 | 110  (71.4%) | 2  (1.3%) | 4  (2.6%) | 0  (0.0%) | 31  (20.1%) | 0  (0.0%) | 0  (0.0%) | 0  (0.0%) | 3  (1.9%) |  |
| Bulut, D. G., et al.[77] | 2015 | Turkish | 621 | 585  (94.2%) | 4  (0.6%) | 7  (1.1%) | 5  (0.8%) | 20  (3.2%) | 0  (0.0%) | 0  (0.0%) | 0  (0.0%) | 0  (0.0%) |  |
| Felsypremila, G., et al.[35] | 2015 | Indian | 438 | 411  (94.0%) | 6  (1.3%) | 0  (0.0%) | 12  (2.7%) | 6  (1.3%) | 0  (0.0%) | 0  (0.0%) | 0  (0.0%) | 0  (0.0%) |  |
| Llena, C., et al.[103] | 2014 | Spanish | 73 | 57  (78.1%) | 6  (8.2%) | 0  (0.0%) | 0  (0.0%) | 8  (11.0%) | 1  (1.4%) | 0  (0.0%) | 0  (0.0%) | 1  (1.4%) |  |
| Ok, E., et al.[79] | 2014 | Turkish | 1471 | 1366  (92.9%) | 4  (0.3%) | 14  (1.0%) | 21  (1.4%) | 65  (4.4%) | 0  (0.0%) | 0  (0.0%) | 1  (0.1%) | 0  (0.0%) |  |
| Shetty, A., et al.[112] | 2014 | Indian | 1186 | 994  (83.8%) | 4  (0.3%) | 25  (2.1%) | 3  (0.3%) | 142  (12.0%) | 2  (0.2%) | 0  (0.0%) | 4  (0.3%) | 1  (0.1%) |  |
| Salarpour, M., et al.[113] | 2013 | Iranian | 42 | 30  (71.4%) | 0  (0.0%) | 0  (0.0%) | 0  (0.0%) | 12  (28.6%) | 0  (0.0%) | 0  (0.0%) | 0  (0.0%) | 0  (0.0%) |  |
| Total |  |  | 14937 | 11333  (75.9%) | 345  (2.3%) | 578  (3.9%) | 477  (3.2%) | 1916  (12.8%) | 96  (0.6%) | 26  (0.2%) | 37  (0.2%) | 111  (0.7%) |  |
| Mandibular PM2s | | | | | | | | | | | | |  |
| Erkan, E., et al.[44] | 2023 | Turkish | 701 | 669  (95.4%) | 8  (1.1%) | 3  (0.4%) | 5  (0.7%) | 12  (1.7%) | 1  (0.1%) | 0  (0.0%) | 3  (0.4%) | 1  (0.1%) |  |
| Mirah, M.A., et al.[46] | 2023 | Saudi | 600 | 539  (89.8%) | 6  (1.0%) | 5  (0.8%) | 4  (0.7%) | 46  (7.7%) | 0  (0.0%) | 0  (0.0%) | 0  (0.0%) | 0  (0.0%) |  |
| Khanna, S et al.[48] | 2023 | Gujarati | 134 | 124  (92.5%) | 4  (3.0%) | 3  (2.2%) | 0  (0.0%) | 0  (0.0%) | 3  (2.2%) | 0  (0.0%) | 0  (0.0%) | 0  (0.0%) |  |
| Iqbal, A., et al.[51] | 2022 | Saudi Arabian | 387 | 383  (99.0%) | 1  (0.3%) | 1  (0.3%) | 0  (0.0%) | 2  (0.5%) | 0  (0.0%) | 0  (0.0%) | 0  (0.0%) | 0  (0.0%) |  |
| Gündüz, H. et al.[23] | 2022 | Turkish | 974 | 934  (95.9%) | 0  (0.0%) | 4  (0.4%) | 11  (1.1%) | 20  (2.1%) | 0  (0.0%) | 0  (0.0%) | 3  (0.3%) | 2  (0.2%) |  |
| Mashyakhy, M., et al.[87] | 2022 | Saudi | 379 | 367  (96.8%) | 0  (0.0%) | 6  (1.6%) | 0  (0.0%) | 3  (0.8%) | 0  (0.0%) | 0  (0.0%) | 0  (0.0%) | 3  (0.8%) |  |
| Lemos, M.C., et al.[106] | 2022 | Brazil | 315 | 302  (95.9%) | 2  (0.6%) | 1  (0.3%) | 0  (0.0%) | 9  (2.9%) | 0  (0.0%) | 0  (0.0%) | 1  (0.3%) | 0  (0.0%) |  |
| Alghamdi, et al.[40] | 2022 | Saudi | 2400 | 2350  (97.9%) | 28  (1.2%) | 2  (0.1%) | 14  (0.6%) | 4  (0.2%) | 2  (0.1%) | 0  (0.0%) | 0  (0.0%) | 0  (0.0%) |  |
| Hasheminia, S.M., et al.[25] | 2021 | Iranian | 384 | 321  (83.6%) | 17  (4.4%) | 3  (0.8%) | 1  (0.3%) | 30  (7.8%) | 4  (1.0%) | 0  (0.0%) | 0  (0.0%) | 8  (2.1%) |  |
| Mishra, S., et al.[92] | 2021 | Delhi-NCR | 216 | 130  (60.2%) | 67  (31.0%) | 6  (2.8%) | 1  (0.5%) | 7  (3.2%) | 4  (1.9%) | 1  (0.5%) | 0  (0.0%) | 0  (0.0%) |  |
| Alenezi, D.J., et al.[94] | 2020 | Kuwaiti | 231 | 30  (13.0%) | 46  (19.9%) | 26  (11.3%) | 20  (8.7%) | 9  (3.9%) | 34  (14.7%) | 4  (1.7%) | 0  (0.0%) | 56  (24.2%) |  |
| Shemesh, A., et al.[109] | 2020 | Israeli | 1678 | 1628  (97.0%) | 6  (0.4%) | 23  (1.4%) | 1  (0.1%) | 10  (0.6%) | 0  (0.0%) | 0  (0.0%) | 4  (0.2%) | 6  (0.4%) |  |
| Alfawaz, H., et al.[26] | 2019 | Saudi | 343 | 309  (90.1%) | 15  (4.4%) | 1  (0.3%) | 9  (2.6%) | 3  (0.9%) | 0  (0.0%) | 0  (0.0%) | 6  (1.7%) | 0  (0.0%) |  |
| Buyukbayram, I. K., et al.[110] | 2019 | Turkish | 264 | 258  (97.7%) | 0  (0.0%) | 3  (1.1%) | 0  (0.0%) | 1  (0.4%) | 0  (0.0%) | 0  (0.0%) | 0  (0.0%) | 2  (0.8%) |  |
| Corbella, S., et al.[95] | 2019 | Caucasians | 88 | 84  (95.5%) | 0  (0.0%) | 0  (0.0%) | 4  (4.5%) | 0  (0.0%) | 0  (0.0%) | 0  (0.0%) | 0  (0.0%) | 0  (0.0%) |  |
| Martins, J. N. R., et al.[114] | 2018 | Portuguese | 858 | 821  (95.7%) | 7  (0.8%) | 11  (1.3%) | 4  (0.5%) | 12  (1.4%) | 0  (0.0%) | 0  (0.0%) | 0  (0.0%) | 3  (0.3%) |  |
| Pedemonte, E., et al.[97] | 2018 | Belgium | 101 | 93  (92.1%) | 0  (0.0%) | 3  (3.0%) | 0  (0.0%) | 5  (5.0%) | 0  (0.0%) | 0  (0.0%) | 0  (0.0%) | 0  (0.0%) |  |
| Burklein, S., et al.[27] | 2017 | German | 870 | 340  (39.1%) | 10  (1.1%) | 1  (0.1%) | 12  (1.4%) | 497  (57.1%) | 4  (0.5%) | 3  (0.3%) | 3  (0.3%) | 0  (0.0%) |  |
| Hajihassani, N., et al.[99] | 2017 | Iranian | 100 | 78  (78.0%) | 3  (3.0%) | 11  (11.0%) | 0  (0.0%) | 7  (7.0%) | 1  (1.0%) | 0  (0.0%) | 0  (0.0%) | 0  (0.0%) |  |
| Martins, J. N. R., et al.[73] | 2017 | Caucasian | 833 | 797  (95.7%) | 7  (0.8%) | 11  (1.3%) | 4  (0.5%) | 12  (1.4%) | 0  (0.0%) | 0  (0.0%) | 0  (0.0%) | 2  (0.2%) |  |
| Arslan, H., et al.[111] | 2015 | Turkish | 133 | 123  (92.5%) | 3  (2.3%) | 1  (0.8%) | 0  (0.0%) | 2  (1.5%) | 0  (0.0%) | 0  (0.0%) | 0  (0.0%) | 2  (1.5%) |  |
| Bulut, D. G., et al.[77] | 2015 | Turkish | 555 | 549  (98.9%) | 1  (0.2%) | 2  (0.4%) | 0  (0.0%) | 3  (0.5%) | 0  (0.0%) | 0  (0.0%) | 0  (0.0%) | 0  (0.0%) |  |
| Felsypremila, G., et al.[35] | 2015 | Indian | 398 | 392  (98.4%) | 0  (0.0%) | 0  (0.0%) | 0  (0.0%) | 3  (0.8%) | 0  (0.0%) | 0  (0.0%) | 0  (0.0%) | 0  (0.0%) |  |
| Llena, C., et al.[103] | 2014 | Spanish | 53 | 48  (90.6%) | 1  (1.9%) | 0  (0.0%) | 0  (0.0%) | 4  (7.5%) | 0  (0.0%) | 0  (0.0%) | 0  (0.0%) | 0  (0.0%) |  |
| Ok, E., et al.[79] | 2014 | Turkish | 1345 | 1325  (98.5%) | 1  (0.1%) | 1  (0.1%) | 8  (0.6%) | 7  (0.5%) | 0  (0.0%) | 0  (0.0%) | 3  (0.2%) | 0  (0.0%) |  |
| Shetty, A., et al.[112] | 2014 | Indian | 814 | 761  (93.5%) | 12  (1.5%) | 2  (0.2%) | 0  (0.0%) | 32  (3.9%) | 0  (0.0%) | 0  (0.0%) | 1  (0.1%) | 0  (0.0%) |  |
| Salarpour, M., et al.[113] | 2013 | Iranian | 41 | 31  (75.6%) | 0  (0.0%) | 0  (0.0%) | 0  (0.0%) | 9  (22.0%) | 0  (0.0%) | 0  (0.0%) | 0  (0.0%) | 1  (2.4%) |  |
| Total |  |  | 15195 | 13786  (90.7%) | 245  (1.6%) | 130  (0.9%) | 98  (0.6%) | 749  (4.9%) | 53  (0.3%) | 8  (0.1%) | 24  (0.2%) | 86  (0.6%) |  |

Supplementary Table 8. Root canal configuration and Asians.

| Studies | Year | Population | N3 | Vertucci I | Vertucci II | Vertucci III | Vertucci IV | Vertucci V | Vertucci VI | Vertucci VII | Vertucci VIII | Others |
| --- | --- | --- | --- | --- | --- | --- | --- | --- | --- | --- | --- | --- |
| Maxillary PM1s | | | | | | | | | | | | |
| Alnaqbi, H.S.Y., et al.[54] | 2022 | South Asian | 53 | 1  (1.9%) | 1  (1.9%) | 0  (0.0%) | 5  (9.4%) | 31  (58.5%) | 4  (7.5%) | 4  (7.5%) | 0  (0.0%) | 7  (13.2%) |
| Yoza T et al.[59] | 2021 | Japan | 125 | 32  (25.6%) | 34  (27.2%) | 4  (3.2%) | 47  (37.6%) | 6  (4.8%) | 0  (0.0%) | 0  (0.0%) | 1  (0.8%) | 1  (0.8%) |
| Liu et al.[36] | 2021 | Chinese | 880 | 245  (27.8%) | 181  (20.6%) | 9  (1.0%) | 390  (44.3%) | 29  (3.3%) | 13  (1.5%) | 9  (1.0%) | 4  (0.5%) | 0  (0.0%) |
| Wu, D., et al.[64] | 2020 | Chinese | 1268 | 132  (10.4%) | 307  (24.2%) | 5  (0.4%) | 743  (58.6%) | 62  (4.9%) | 11  (0.9%) | 0  (0.0%) | 5  (0.4%) | 3  (0.2%) |
| Martins, J. N. R., et al.[70] | 2018 | Asian | 238 | 29  (12.2%) | 54  (22.7%) | 10  (4.2%) | 131  (55.0%) | 14  (5.9%) | 0  (0.0%) | 0  (0.0%) | 0  (0.0%) | 0  (0.0%) |
| Li, Y.H., et al.[41] | 2018 | Chinese | 1387 | 163  (11.8%) | 204  (14.7%) | 223  (16.1%) | 592  (42.7%) | 168  (12.1%) | 9  (0.6%) | 10  (0.7%) | 10  (0.7%) | 8  (0.6%) |
| Tian, Y. Y., et al.[80] | 2012 | Chinese | 300 | 43  (14.3%) | 70  (23.3%) | 13  (4.3%) | 153  (51.0%) | 10  (3.3%) | 7  (2.3%) | 2  (0.7%) | 2  (0.7%) | 0  (0.0%) |
| Shi, Z.-Y., et al.[74] | 2017 | Chinese | 521 | 21  (4.0%) | 150  (28.8%) | 0  (0.0%) | 271  (52.0%) | 7  (1.3%) | 47  (9.0%) | 1  (0.2%) | 10  (1.9%) | 14  (2.7%) |
| Pan, J. Y. Y., et al.[67] | 2019 | Malaysian | 304 | 133  (43.8%) | 49  (16.1%) | 32  (10.5%) | 46  (15.1%) | 11  (3.6%) | 28  (9.2%) | 5  (1.6%) | 0  (0.0%) | 0  (0.0%) |
| Total |  |  | 5076 | 799  (15.7%) | 1050  (20.7%) | 296  (5.8%) | 2378  (46.8%) | 338  (6.7%) | 119  (2.3%) | 31  (0.6%) | 32  (0.6%) | 33  (0.7%) |
| Maxillary PM2s | | | | | | | | | | | | |
| Alnaqbi, H.S.Y., et al.[54] | 2022 | South Asian | 52 | 0  (0.0%) | 4  (7.7%) | 8  (15.4%) | 3  (5.8%) | 13  (25.0%) | 5  (9.6%) | 7  (13.5%) | 0  (0.0%) | 12  (23.1%) |
| Yan, Y., et al.[84] | 2021 | western Chinese | 1118 | 616  (55.1%) | 357  (31.9%) | 6  (0.5%) | 114  (10.2%) | 17  (1.5%) | 5  (0.4%) | 1  (0.1%) | 2  (0.2%) | 0  (0.0%) |
| Pan, J. Y. Y., et al.[67] | 2019 | Malaysian | 333 | 204  (61.3%) | 60  (18.0%) | 31  (9.3%) | 6  (1.8%) | 21  (6.3%) | 10  (3.0%) | 1  (0.3%) | 0  (0.0%) | 0  (0.0%) |
| Li, Y.H., et al.[41] | 2018 | Chinese | 1403 | 706  (50.3%) | 146  (10.4%) | 336  (23.9%) | 83  (5.9%) | 112  (8.0%) | 4  (0.3%) | 6  (0.4%) | 0  (0.0%) | 10  (0.7%) |
| Martins, J. N. R., et al.[70] | 2018 | Asian | 239 | 179  (74.9%) | 36  (15.1%) | 2  (0.8%) | 18  (7.5%) | 4  (1.7%) | 0  (0.0%) | 0  (0.0%) | 0  (0.0%) | 0  (0.0%) |
| Shi, Z.-Y., et al.[74] | 2017 | Chinese | 517 | 157  (30.4%) | 207  (40.0%) | 3  (0.6%) | 69  (13.3%) | 16  (3.1%) | 47  (9.1%) | 3  (0.6%) | 1  (0.2%) | 14  (2.7%) |
| Yang, L., et al.[85] | 2014 | Chinese | 392 | 178  (45.4%) | 64  (16.3%) | 45  (11.5%) | 79  (20.2%) | 25  (6.4%) | 0  (0.0%) | 0  (0.0%) | 1  (0.3%) | 0  (0.0%) |
| Total |  |  | 4054 | 2040  (50.3%) | 874  (21.6%) | 431  (10.6%) | 372  (9.2%) | 208  (5.1%) | 71  (1.8%) | 18  (0.4%) | 4  (0.1%) | 36  (0.9%) |
| Mandibular PM1s | | | | | | | | | | | | |
| Choi et al.[108] | 2022 | Korean | 1463 | 1254  (85.7%) | 4  (0.3%) | 65  (4.4%) | 0  (0.0%) | 140  (9.6%) | 0  (0.0%) | 0  (0.0%) | 0  (0.0%) | 0  (0.0%) |
| Thanaruengrong et al.[88] | 2021 | Thai | 621 | 392  (63.1%) | 9  (1.4%) | 16  (2.6%) | 4  (0.6%) | 177  (28.5%) | 2  (0.3%) | 1  (0.2%) | 0  (0.0%) | 20  (3.2%) |
| Arayasantiparb, R. et al.[90] | 2021 | Thai | 349 | 281  (80.5%) | 2  (0.6%) | 1  (0.3%) | 0  (0.0%) | 59  (16.9%) | 0  (0.0%) | 1  (0.3%) | 0  (0.0%) | 5  (1.4%) |
| Wu, D., et al.[64] | 2020 | Chinese | 1296 | 1051  (81.1%) | 0  (0.0%) | 39  (3.0%) | 0  (0.0%) | 160  (12.3%) | 0  (0.0%) | 0  (0.0%) | 6  (0.5%) | 0  (0.0%) |
| Pan, J. Y. Y., et al.[67] | 2019 | Malaysian | 365 | 301  (82.5%) | 1  (0.3%) | 5  (1.4%) | 3  (0.8%) | 55  (15.1%) | 0  (0.0%) | 0  (0.0%) | 0  (0.0%) | 0  (0.0%) |
| Jang, Y. E., et al.[96] | 2019 | Korean | 1007 | 765  (76.0%) | 22  (2.2%) | 36  (3.6%) | 4  (0.4%) | 134  (13.3%) | 0  (0.0%) | 1  (0.1%) | 0  (0.0%) | 9  (0.9%) |
| Martins, J. N. R., et al.[70] | 2018 | Asian | 238 | 206  (86.6%) | 0  (0.0%) | 0  (0.0%) | 1  (0.4%) | 30  (12.6%) | 0  (0.0%) | 0  (0.0%) | 0  (0.0%) | 1  (0.4%) |
| Vega-Lizama et al.[98] | 2018 | Yucatecan | 146 | 58  (39.7%) | 0  (0.0%) | 24  (16.4%) | 0  (0.0%) | 31  (21.2%) | 0  (0.0%) | 4  (2.7%) | 0  (0.0%) | 29  (19.9%) |
| Yang, H., et al.[104] | 2013 | Chinese | 440 | 335  (76.1%) | 15  (3.4%) | 12  (2.7%) | 29  (6.6%) | 41  (9.3%) | 0  (0.0%) | 0  (0.0%) | 3  (0.7%) | 0  (0.0%) |
| Yu, X., et al.[105] | 2012 | Chinese | 174 | 151  (86.8%) | 0  (0.0%) | 3  (1.7%) | 0  (0.0%) | 17  (9.8%) | 0  (0.0%) | 0  (0.0%) | 1  (0.6%) | 0  (0.0%) |
| Total |  |  | 6099 | 4794  (78.6%) | 53  (0.9%) | 201  (3.3%) | 41  (0.7%) | 844  (13.8%) | 2  (<0.1%) | 7  (0.1%) | 10  (0.2%) | 64  (1.0%) |
| Mandibular PM2s | | | | | | | | | | | | |
| Choi et al.[108] | 2022 | Korean | 1448 | 1441  (99.5%) | 0  (0.0%) | 4  (0.3%) | 0  (0.0%) | 3  (0.2%) | 0  (0.0%) | 0  (0.0%) | 0  (0.0%) | 0  (0.0%) |
| Thanaruengrong et al.[88] | 2021 | Thai | 538 | 527  (98.0%) | 1  (0.2%) | 2  (0.4%) | 0  (0.0%) | 8  (1.5%) | 0  (0.0%) | 0  (0.0%) | 0  (0.0%) | 0  (0.0%) |
| Arayasantiparb, R. et al.[90] | 2021 | Thai | 416 | 400  (96.2%) | 0  (0.0%) | 4  (1.0%) | 0  (0.0%) | 12  (2.9%) | 0  (0.0%) | 0  (0.0%) | 0  (0.0%) | 0  (0.0%) |
| Pan, J. Y. Y., et al.[67] | 2019 | Malaysian | 399 | 397  (99.5%) | 1  (0.3%) | 0  (0.0%) | 1  (0.3%) | 0  (0.0%) | 0  (0.0%) | 0  (0.0%) | 0  (0.0%) | 0  (0.0%) |
| Jang, Y. E., et al.[96] | 2019 | Korean | 997 | 981  (98.4%) | 14  (1.4%) | 2  (0.2%) | 0  (0.0%) | 0  (0.0%) | 0  (0.0%) | 0  (0.0%) | 0  (0.0%) | 0  (0.0%) |
| Martins, J. N. R., et al.[70] | 2018 | Asian | 236 | 235  (99.6%) | 1  (0.4%) | 0  (0.0%) | 0  (0.0%) | 0  (0.0%) | 0  (0.0%) | 0  (0.0%) | 0  (0.0%) | 0  (0.0%) |
| Yu, X., et al.[105] | 2012 | Chinese | 178 | 173  (97.2%) | 1  (0.6%) | 0  (0.0%) | 0  (0.0%) | 3  (1.7%) | 0  (0.0%) | 0  (0.0%) | 0  (0.0%) | 0  (0.0%) |
| Total |  |  | 4212 | 4154  (98.6%) | 18  (0.4%) | 12  (0.3%) | 1  (<0.1%) | 26  (0.6%) | 0  (0.0%) | 0  (0.0%) | 0  (0.0%) | 0  (0.0%) |
